# Chemical Upcycling of Polyphenylene Sulfide at Room Temperature

**DOI:** 10.1002/advs.202519090

**Published:** 2025-12-14

**Authors:** Boning Gu, Chengliang Li, Xuefeng Jiang

**Affiliations:** ^1^ Hainan Institute of East China Normal University State Key Laboratory of Petroleum Molecular & Process Engineering Shanghai Key Laboratory of Green Chemistry and Chemical Processes School of Chemistry and Molecular Engineering East China Normal University Shanghai 200062 China; ^2^ State Key Laboratory of Organometallic Chemistry Shanghai Institute of Organic Chemistry Chinese Academy of Sciences Shanghai 200032 China

**Keywords:** C–S bond cleavage, chemical upcycling, photocatalysis, plastic waste, polyphenylene sulfide

## Abstract

Polyphenylene sulfide (PPS) is a semi‐crystalline super engineering plastic with exceptional chemical resistance, thermal stability, and mechanical integrity. The rapidly growing consumption of PPS underscores an urgent demand for sustainable recycling strategies to promote circularity in the PPS industry. Chemical upcycling offers a compelling and sustainable strategy for plastic waste valorization. Herein, an efficient iron‐photocatalyzed chemical upcycling of PPS plastics with 1,2‐dichloroethane at room temperature is reported, affording recuperative chlorinated arylene monomer and valuable chloroacetic acid. Mechanistic investigation demonstrates chlorinated phenylthiyl species as the key intermediates generated from chlorine radical to mediate aryl C─S bond cleavage. The protocol establishes broad compatibility of eighteen commercial PPS resins and composite waste with gram‐scaled efficiency and effective degradation for mixed‐plastic matrix, offering a transformative paradigm for super engineering plastic upcycling.

## Graphical abstract

Poly(phenylene sulfide) (PPS) is a semi‐crystalline super engineering plastic with exceptional properties. An iron‐photocatalyzed strategy is reported for chemical upcycling of PPS plastics at room temperature using 1,2‐dichloroethane. The method demonstrates remarkable efficiency across diverse PPS resins and post‐consumer composites, even within mixed‐plastic waste streams, offering a practical and sustainable pathway for super engineering plastic upcycling.

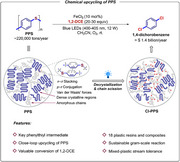

## 1 Introduction

**Figure 1:**
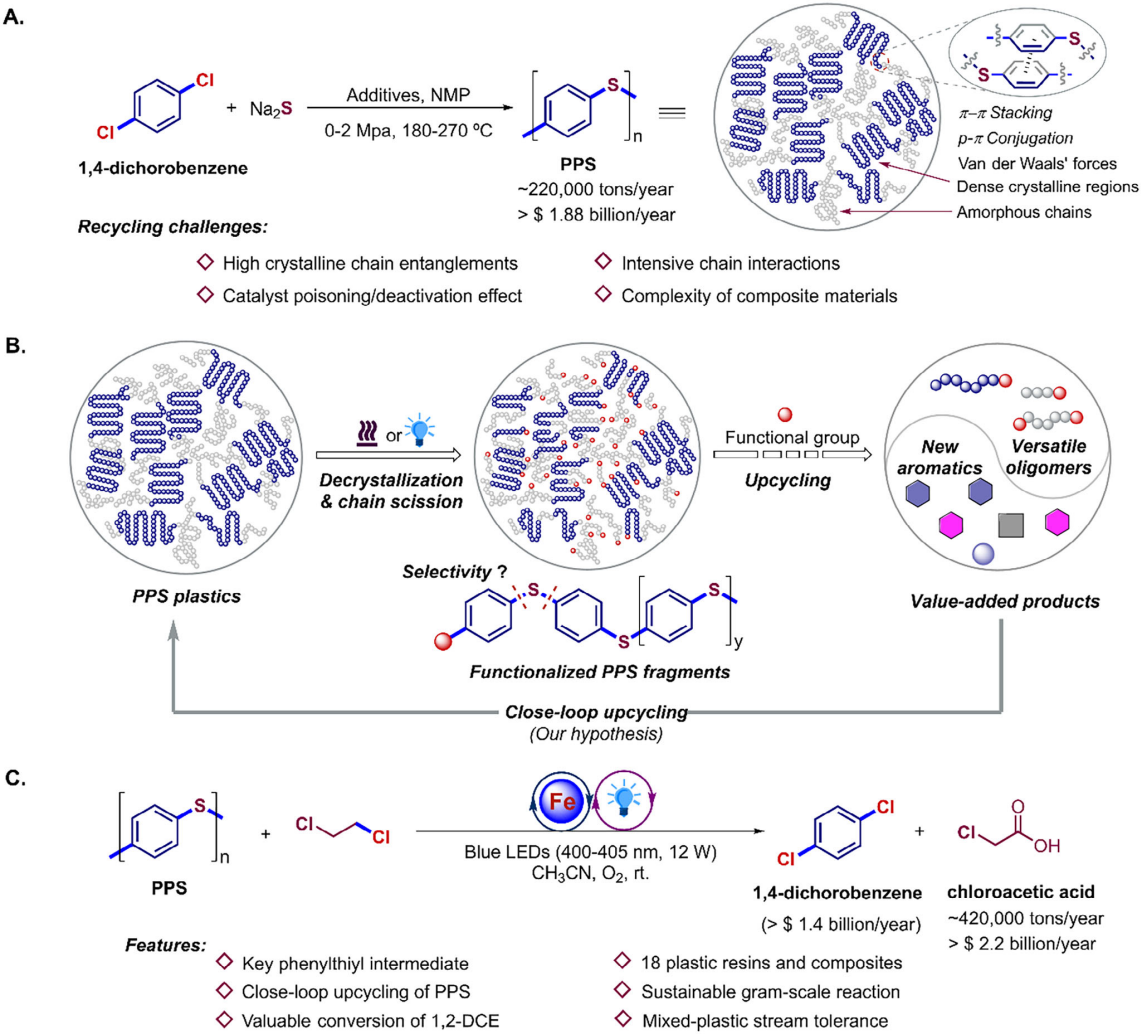
Chemical upcycling of PPS. A) Commonly, industrial production of PPS and present upcycling challenges. B) Conceptual pathways for sustainable PPS upcycling. C) This work: Chemical upcycling of PPS via iron‐photocatalysis at room temperature.

Sulfur‐based polyarylene plastics represent a critical class of advanced functional polymers engineered via strategically incorporating sulfur within the rigid aromatic architectures, exemplified by commercial polyarylene sulfide (PAS).^[^ ^1^, ^2^, ^3^ ^]^ Particularly, polyphenylene sulfide (PPS) stands as a semi‐crystalline super engineering thermo‐plastic (**Figure** **1**A),^[^ ^4^, ^5^, ^6^ ^]^ possessing superior chemical resistance with virtual insolubility below 170 °C, exceptional thermal performance with melting temperature over 280 °C and continuous service temperature exceeding 200 °C, as well as inherent mechanical property such as flexural strength up to 200 MPa and tensile strength reaching 100 MPa^[^ ^7^ ^]^ Surging demand for PPS plastic and its composites, as lightweight and cost‐effective alternatives for metallic components in high‐end materials, has driven the global consumption beyond 220 000 tonnes annually with a projected compound annual growth rate (CAGR) of 7.8% by 2035.^[^ ^8^ ^]^ Meanwhile, the rapid accumulation of end‐of‐life PPS materials has raised serious environmental crises and resource waste. Inadequate management, such as landfill and incineration, gives rise to the release of persistent toxic sulfur species^[^ ^9^ ^]^ and substantial greenhouse gases,^[^ ^10^ ^]^ while the feasible mechanical recycling manner acquires diminished material properties,^[^ ^11^ ^]^ thereby underscoring an urgent demand for sustainable upcycling strategies to promote the circularity in the PPS industry.^[^ ^12^ ^,^ ^13^ ^]^

Chemical recycling provides innovative routes for plastic waste reutilization back to value‐added monomers and upcycled feedstocks.^[^ ^14^, ^15^, ^16^, ^17^, ^18^, ^19^, ^20^, ^21^ ^]^ Nevertheless, PPS upcycling has remained exceptionally challenging,^[^ ^22^, ^23^ ^]^ primarily due to the inherent features including the high crystalline (up to 80%) with polymer entanglements and intensive inter‐/intra‐chain interactions reinforced by *π–π* stacking, *p‐π* conjugation and Van der Waals' forces among rigid aryl C─S moieties, as well as the extensively documented catalyst poisoning leading to deactivation imparted by sulfur species (Figure 1A). Simultaneously, real‐life PPS composites, blended with up to 70 wt% of heterogeneous ingredients, posed their upcycling much more intractable, including other plastics, glass fibers, anti‐ultraviolet stabilizers, adhesives, masterbatches, and antioxidants.^[^ ^4^, ^24^ ^]^ A landmark advancement in palladium‐catalyzed carbon‐sulfur metathesis was achieved by Morandi and colleagues, including one notable example of pristine PPS resin upcycling,^[^ ^25^ ^]^ yet approaches to conceptual closed‐loop upcycling of end‐of‐life PPS resins and composites remain elusive (Figure 1B). Continuously with our research on plastic recycling and upcycling,^[^ ^26^, ^27^, ^28^ ^]^ we report an efficient chemical upcycling of PPS plastics with 1,2‐dichloroethane via an iron‐photocatalysis strategy at room temperature (Figure 1C), affording 1,4‐dichlorobenzene monomer (market value > $ 1.4 billion)^[^ ^29^ ^]^ with valuable chloroacetic acid synchronously (annual production: ≈420 000 tons, market valued > $ 2.2 billion).^[^ ^30^ ^]^ Mechanistic studies indicate the chlorinated phenylthiyl as the key intermediate formed via chlorine radical‐mediated cleavage of the aryl C─S bond in both PPS and functionalized‐PPS fragments.

## 2 Results and Discussion

### 2.1 Desulfurizing Chlorination of Thioethers

**Figure 2:**
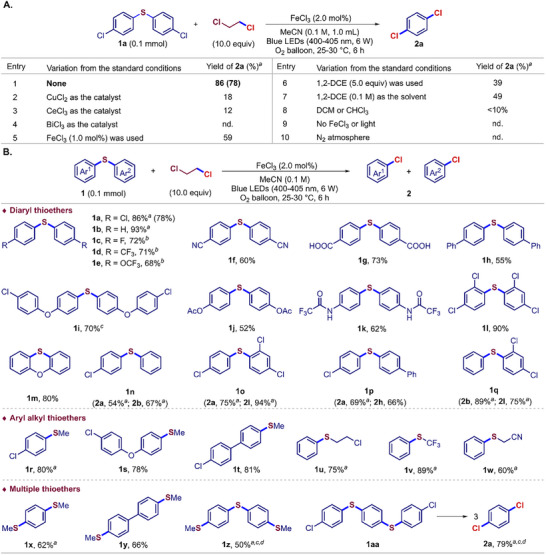
Desulfurizing chlorination of thioethers. A) Optimizing the reaction conditions. B) The scope of functionalized thioethers. Thioethers (0.1 mmol) were tested for three trials. Isolated yields of **2** were calculated on a molar basis of aryl rings. *^a^* GC yields of volatile products were given. *^b^* ^19^F NMR yields. *^c^* Reaction for 24 h. *^d^* FeCl_3_ (4.0 mol%) and 1,2‐DCE (2.0 mol) were used.

We commenced our studies on PPS upcycling with a metal‐photocatalyzed desulfurizing chlorination of bis(4‐chlorophenyl)sulfane (**1a**). Systematically screening the reaction conditions revealed that the dual desulfurizing chlorination of **1a** afforded 1,4‐dichlorobenzene (**2a**) in 86% GC yield (**Figure** **2**A; Table S1, Supporting Information), utilizing 2 mol% of iron chloride and ten equivalents of 1,2‐dichloroethane in acetonitrile (0.1 m) under blue light irradiation (400‐405 nm, 6 W)^[^ ^31^ ^]^ with an oxygen balloon at 25–30 °C for 6 h (Figure 2A, entry 1). The formation of chloroacetic acid (**3**) as the sole product from the dechlorination of 1,2‐dichloroethanewas confirmed by nuclear magnetic resonance (NMR) spectroscopy and gas chromatography‐mass spectrometry (GC‐MS). (Figures S2 and S3, Supporting Information). Alternative metal catalysts, including copper chloride and cerium chloride, resulted in dramatically reduced yields; even bismuth chloride quenched the reaction completely (entries 2–4). Reducing the iron chloride loading to 1 mol% decreased the production of **2a** to 59% yield (entry 5). Five equivalents and neat 1,2‐dichloroethane afforded **2a** in 39% and 49% yields, respectively (entries 6 and 7). Other chlorinated alkanes, such as dichloromethane and chloroform, exhibited much lower efficiency (entry 8). Iron chloride, light irradiation, and oxygen atmosphere were essential for this reaction (entries 9 and 10).

The compatibility of functionalized thioethers (**1a**‐**1aa**) was investigated under the optimized conditions, affording the corresponding aryl chlorides in up to 94% yields (Figure 2B). A variety of functional groups were well tolerated, including halides (**1a**, **1c**, **1l**, and **1n**‐**1q**), ether (**1e**, **1i**, and **1m**), cyano (**1f**), carboxy (**1g**), ester (**1j**), amide (**1k**), and *π*‐extended aromatic rings (**1h** and **1m**). Aryl alkyl thioethers (**1r**‐**1y**) were efficiently converted into the corresponding aryl chlorides and alkyl derivatives. Aryl thioethers bearing electron‐withdrawing trifluoromethyl (**1v**) and cyanomethyl (**1w**) groups were compatible as well, despite their tendency toward C(*sp^3^*)–S bond cleavage. Bis(4‐(methylthio)phenyl)sulfane (**1z**) and bis(4‐((4‐chlorophenyl)thio)phenyl)sulfane (**1aa**) with multiple aryl C(*sp^2^*)–S bonds (**1x**‐**1aa**) enabled consecutively desulfurizing chlorination with high efficiency.

### 2.2 Chemical Upcycling of PPS Plastics

**Figure 3:**
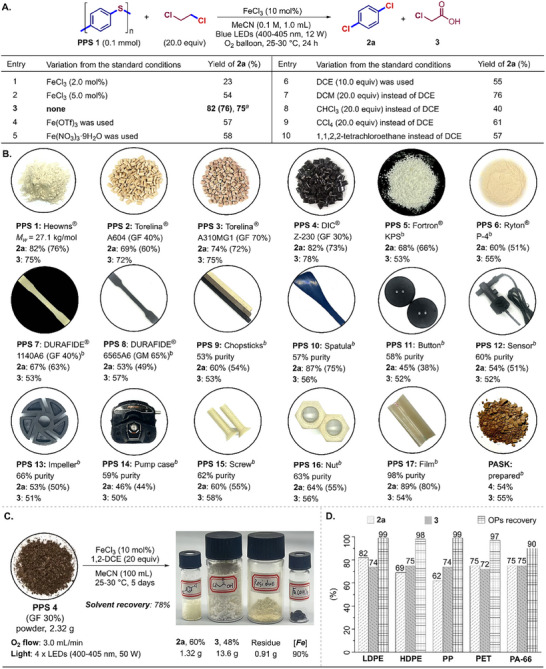
Chemical upcycling of PPS materials. A) Optimizing the conditions. B) The compatibility of PPS plastics. PPS (0.1 mmol per repeated unit) for three trials. GC yields and isolated yields in parentheses of **2a** were calculated on a molar basis. *^a^* NMR yields of **3** were calculated on the molar basis of 1,2‐DCE. *^b^* 1,2‐DCE (30.0 equiv). C) Gram‐scaled chemical upcycling reaction. D) Treatment of **PPS 4** blending with other plastics (OPs). GF = glass fiber. GM = glass mineral.

This iron‐photocatalytic strategy was applied to the feasibility of PPS plastic upcycling (**Figures** **3**; S20, Supporting Information). A commercial PPS resin (**PPS 1**) with a weight‐average molecular weight (*M_w_*) of 27.1 kg mol^−1^ afforded **2a** in only 23% yield in a solid–liquid heterogeneous system, much lower than homogeneous thioethers (Figure 3A, entry 1). Increasing iron chloride loading from 2 to 10 mol% improved the yield of **2a** to 82% with **3** in 75% yield (entries 2 and 3), whereas other iron salts exhibited inferior efficiency (entries 4 and 5). Other chlorinated alkanes were also feasible chlorine sources, albeit with lower efficiencies (entries 6–10). Diverse PPS resins and composite materials were subsequently subjected to this iron‐photocatalyzed upcycling protocol (Figure 3B). PPS powders and granules containing pigments and reinforced glass fibers (**PPS 2**–**6**) proceeded dual desulfurizing chlorination reaction with 1,2‐dichloroethane under the optimized conditions, affording **2a** in 60–82% yields and **3** in 53–78% yields, respectively. Composite dog‐bone‐shaped samples (**PPS 7** and **8**) could be degraded to **2a** in moderate yields. Furthermore, various end‐of‐life PPS materials were evaluated. Chemical upcycling of kitchen utensils such as chopsticks (**PPS 9**) and spatulas (**PPS 10**), and dark buttons from the clothing industry (**PPS 11**), furnished **2a** in up to 87% yields. Precision instruments, fabricated from composite PPS materials with ≈60% purities, were converted to the corresponding monomers, including the dark sensor probe (**PPS 12**), impeller (**PPS 13**), pump case (**PPS 14**), as well as screw bolts and nuts (**PPS 15** and **16**). Noteworthily, PPS thin films (**PPS 17**) were smoothly upcycled to afford **2a** in 89% yield, which has been an emerging alternative for traditional liquid crystal polymers (LCP) films derived from polyesters and polyamides.^[^ ^32^ ^]^ Polyarylene sulfide ketone (**PASK**), a newly‐developed PAS with enhanced properties (*T_m_* = 362.3 °C),^[^ ^33^ ^]^ underwent the iron‐photocatalyzed dual desulfurizing chlorination with 1,2‐dichloroethane to afford the corresponding 4,4′‐dichlorobenzophenone monomer (**4**) in 54% yield.

To demonstrate the practicability of this protocol, a liquid‐solid reaction mixture containing 2.32 g of the black resin (**PPS 4**) was irradiated under blue light for five days with a continuous oxygen flow at a rate of 3.0 mL min^−1^, affording 1.32 g of **2a**, 13.6 g of **3**, and 0.91 g of oligomeric residues/impurities (Figures 3C; S40, Supporting Information). Meanwhile, the iron species was recovered in 90% yield, and 78% of mixed solvents was recycled by volume. Simulated treatment of real composite PPS materials was performed by blending **PPS 4** with equal masses of various common plastics, including low‐density polyethylene (LDPE) gloves, high‐density polyethylene (HDPE) reagent bottles, polypropylene (PP) centrifuge tubes, polyethylene terephthalate (PET) beverage bottles, and nylon‐66 cable ties (Figures 3D; S41, Supporting Information). Remarkably, specific chemical upcycling of **PPS 4** was achieved efficiently to furnish **2a** and **3** in 62–82% yields and 72–75% yields, respectively. Post‐reaction filtration confirmed negligible mass loss for most non‐PPS plastics, underscoring the potential of such iron‐photocatalysis strategy for PPS valorization in mixed plastic waste streams.

### 2.3 Studies on Chemical Upcycling of PPS

**Figure 4:**
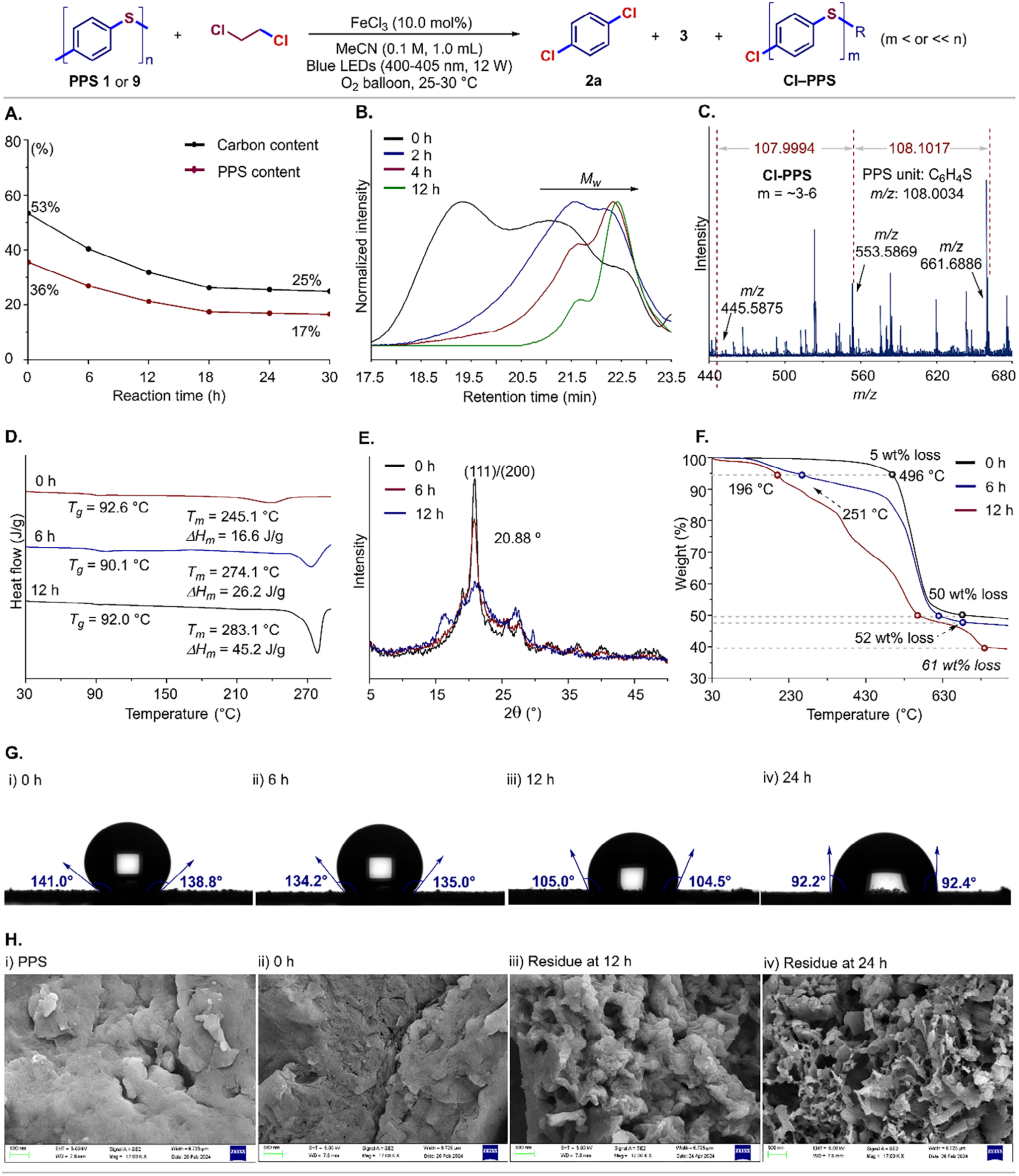
Studies on chemical upcycling of PPS. A) Element analysis tracking experiments of **PPS 9**. B) High‐temperature GPC profiles of **PPS 1**/Cl‐PPS. C) MALDI‐TOF‐MS analysis. D) DSC curves at 0, 6, and 12 h. E) XRD spectra at 0, 6, and 12 h. F) TGA curves at 0, 6, and 12 h. G) Water contact angles at 0, 6, 12, and 24 h. H) SEM images of **PPS 1** and residues at 0, 12, and 24 h.

To investigate the mechanism of PPS upcycling, a series of tracking experiments was performed (**Figure** **4**). Element analysis of the residues from **PPS 9** indicated a decreasing trend of the carbon content, confirming a diminishing proportion of PPS oligomers during the desulfurizing chlorination process (Figure 4A). High‐temperature gel permeation chromatography (GPC) profiles revealed a notable reduction of *M_w_* from 27.1 to 2.6 kg mol^−1^ within 12 h, indicating extensive polymer chain scission (Figure 4B). FTIR spectrum of the oligomeric residue exhibited a newly emerged peak at 1049 cm^−1^, associated with aryl C─Cl stretching vibration (Figure S44, Supporting Information). Further analysis for 24 h residue by MALDI‐TOF‐MS identified peaks matching with the expected Cl‐PPS oligomers (Cl–PPS) in *m/z* values ranking from 240 to 800 (Figures 4C; S45, Supporting Information). Sharp declines were observed in both the melting endotherm (*∆H_m_*) and melting temperature (*T_m_*) via differential scanning calorimetry (DSC) study (Figure 4D), and X‐ray diffraction (XRD) analysis revealed a pronounced attenuation of the characteristic crystalline peak (2θ = 20.88°) (Figure 4E), confirming the destruction of long‐range crystalline order in PPS chains. Cl‐PPS residues exhibited diminished thermal stability compared to original PPS polymers evidenced by thermogravimetric analysis (TGA), decomposing at notably reduced temperature with up to 61 wt% loss (Figure 4F). Under optimal conditions, the water contact angle dropped from 139.9° (**PPS 1**) to 92.3° for the residual oligomers, revealing the enhanced surface wettability (Figure 4G), which was attributed to distinct morphological changes observed via scanning electron microscopy (SEM), including the formation of micropores, fissures, and lamellar structures (Figure 4H). Overall, these observations unequivocally demonstrated that the iron‐photocatalytic strategy both promotes desulfurizing chlorination and drives hierarchical structural disintegration by disrupting the crystalline domains in PPS plastics.

**Figure 5:**
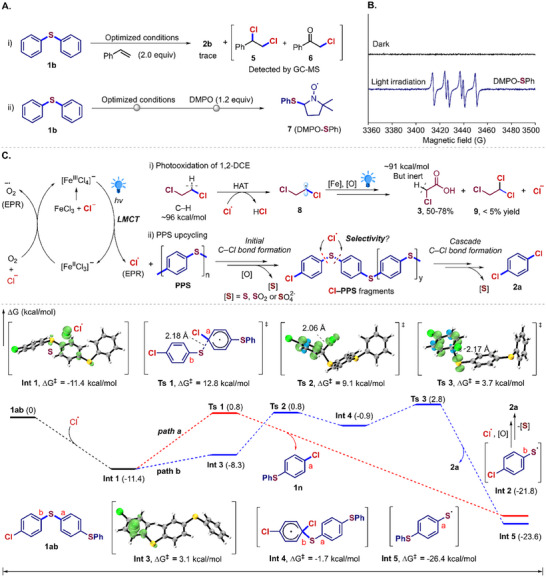
Studies on desulfurizing chlorination of C─S bond. A) Control experiments to verify the possible radical species. B) The EPR experiment to capture the benzenethiyl radical. C) The proposed catalytic pathway and DFT calculations.

To elucidate the mechanism of the aryl C─S bond chlorination, control experiments were conducted comprehensively (**Figure** **5**). PPS upcycling was dramatically suppressed by 2,2,6,6‐tetramethylpiperidine‐1‐oxyl and 2,6‐di‐*tert*‐butyl‐4‐methylphenol, indicating a radical‐mediated pathway (Figure S51, Supporting Information). Ultraviolet‐visible spectroscopy demonstrated the iron chloride as the photocatalyst (Figure S52, Supporting Information). The necessity of the continuous irradiation was revealed via light on/off experiment, excluding the chain propagation pathway (Figure S53, Supporting Information). Additionally, the reaction of **1b** and two equivalents of styrene yielded chlorinated adducts **5** and **6**, verifying the formation of chlorine radicals (Figures 5A; S54, Supporting Information). The pivotal phenylthiyl radical intermediate (**7**) was captured by 5,5‐dimethyl‐1‐pyrroline *N*‐oxide (DMPO) via electron paramagnetic resonance (EPR) experiment (Figure 5B).^[^ ^34^ ^]^ EPR signals, corresponding to chlorine radicals, hydroxyl radicals, and superoxide radicals, were confirmed across the board (Figures S55 and S56, Supporting Information).

Based on the above experimental results, a catalytic route was proposed in Figure 5C. Initially, the iron chloride was coordinated with a chloride anion to form the active [Fe^III^Cl_4_]¯ species, followed by a ligand‐to‐metal charge transfer (LMCT) process under light irradiation to afford the chlorine radical and [Fe^II^Cl_3_]¯ species.^[^ ^35^, ^36^ ^]^ The [Fe^II^Cl_3_]¯ species was reoxidized by oxygen in the presence of a chloride ion to regenerate the [Fe^III^Cl_4_]¯ with the formation of a superoxide radical. Hydrogen‐atom transfer (HAT) of 1,2‐dichloroethane (C─H bond dissociation energy (*BDE*): ≈96 kcal mol^−1^) by the chloride radical generated the 1,2‐dichloroethyl radical (**8**), further oxidized to form the chloroacetic acid (**3**, *BDE* of C─H bond: ≈91 kcal mol^−1^) and chlorides, as well as trapped by a chloride radical to afford 1,1,2‐trichloroethane (**9**) (Figures S58 and S59, Supporting Information).^[^ ^37^ ^]^ The mismatched polarity and steric hindrance effects of chloroacetic acid likely suppressed further HAT/oxidation of the electron‐poor C─H bond.^[^ ^38^, ^39^ ^]^ Theoretically, 1,2‐dichloroethane photooxidation provided up to fifteen equivalents of chlorides to fuel the desulfurizing chlorination reaction. Subsequently, PPS chains reacted with chlorine radicals via a homolytic aromatic substitution (HAS) to afford Cl–PPS fragments and PPS‐thiyl radicals.^[^ ^40^ ^]^ Finally, the PPS‐thiyl radicals converted rapidly into corresponding Cl–PPS fragments with sulfur‐containing species released, including sulfur (0) captured by triphenylphosphine, sulfur dioxide detected by GC‐MS, and sulfate ions precipitated by barium ions (Figures S60–S62, Supporting Information). Control experiments demonstrated that both diaryldisulfanes^[^ ^41^ ^]^ and arylsulfonyl chlorides^[^ ^26^ ^]^ served as the intermediates in this transformation, generated from dimerization and chlorine radical‐mediated oxidation of phenylenethiyl radicals, respectively (Figures S63–S65, Supporting Information).

However, the regioselectivity remained elusive for chain scissions of unsymmetric functionalized‐PPS oligomers during the chemical upcycling of PPS plastics. We carried out density functional theory (DFT) calculations using a representative unsymmetric Cl–PPS fragment (**1ab**) as the model (Figure 5C). Reaction of thioether **1ab** with a chlorine radical afforded **Int 1**, in which the radical is localized on the central phenyl ring. A homolytic aromatic substitution at C_a_─S bond generates the chlorinated benzenethiyl radical **Int 2** and **1n** via a transition state **Ts 1**, overcoming an energy barrier of 12.2 kcal mol^−1^ (*path a*). **Int 1** isomerizes into **Int 3** with the chlorine radical bonded to the chlorinated phenyl ring, which undergoes an intramolecular radical addition followed by homolytic C_b_─S bond cleavage of **Int 4** to afford the phenylthiyl radical **Int 5** and **2a**. The whole step traverses **Int 3**, transition states **Ts 2** and **Ts 3** with an activation barrier of 14.2 kcal mol^−1^ (*path b*). These results indicated that cleavages of more electron‐rich aryl C‒S bonds occurred predominantly within the unsymmetric Cl–PPS fragments (*path a*), resulting in the formation of **Int 2** in a systematic pathway. Further cascade dimerization/oxidation and desulfurizing chlorination of **Int 2** mediated by the chlorine radicals afforded **2a**.

## 3 Conclusion

In summary, we have established the chemical upcycling of semi‐crystalline PPS plastics with 1,2‐dichloroethane via an iron‐photocatalyzed dual desulfurizing chlorination strategy at room temperature. The protocol exhibits broad compatibility with both PPS resins and composite wastes incorporated with diverse unidentified additives, achieving dichloroarylene monomers and commercially valuable chloroacetic acid in up to 89% and 78% yields, respectively. The practical viability is further corroborated through gram‐scaled transformations and mixed‐plastic matrices treatment. The chlorinated phenylthiyl species, as the key intermediates, are demonstrated for the systematic cleavage of aryl carbon‐sulfur bonds in PPS backbones, establishing a sustainable, energy‐efficient, and scalable platform for the closed‐loop upcycling of polyphenylene sulfide plastics.

## Conflict of Interest

The authors declare no conflict of interest.

## Supporting information



Supporting Information

## Data Availability

The data that support the findings of this study are openly available in 0 at https://doi.org/[doi], reference number 0.
