# Supplementary material for: Chemical Upcycling of Polyphenylene Sulfide at Room Temperature
Source: Adv Sci (Weinh). 2025 Dec 14;13(22):e19090. doi: 10.1002/advs.202519090 (PMC13088349; doi:10.1002/advs.202519090)
Supplement: Supplementary file 1 — Supporting Information [file ADVS-13-e19090-s001.pdf]

Supporting Information

Chemical upcycling of polyphenylene sulfides at room temperature

*Boning Gu, Chengliang Li, Xuefeng Jiang**

Tables of Contents

General remarks **2**

The desulfurizing chlorination of aryl thioether models **3**

(i) General procedures for the synthesis of aryl thioether models 3
(ii) Optimization for the desulfurizing chlorination of aryl thioether 11
(iii) General procedure for the desulfurizing chlorination of aryl thioethers 14

The chemical upcycling of PPS resins and plastics **24**

(i) Optimization of the conditions for chemical upcycling of PPS 24
(ii) General procedure for chemical upcycling of PPS resins and plastic 25
(iii) The chemical upcycling of PASK 33
(iv) The gram-scaled chemical upcycling of PPS 34
(v) Mixed-plastic experiments 35

Mechanistic studies on the chemical upcycling of PPS **36**

(i) Reaction tracking by elemental analysis 36
(ii) High-temperature gel permeation chromatography 37
(iii) Fourier transform infrared (FTIR) spectra 38
(iv) MALDI-TOF-MS analysis 39
(v) Differential scanning calorimetry (DSC) measurement 40
(vi) X-ray diffraction (XRD) measurement 40
(vii) Thermogravimetric analysis (TGA) measurement 41
(viii) Water contact angle test 42
(ix) Scanning electron microscopy (SEM) 42

Mechanistic studies on iron-photocatalyzed C–S bond cleavage **43**

(i) Radical quenching experiments 43
(ii) UV-Vis spectroscopy 44
(iii) Light-on/off experiment 44
(iv) The detections of active radical species. 45
(v) The detection of the sulfur species 49
(vi) Proposed catalytic cycle 53

DFT calculations **53**

References **65**

NMR spectra **67**

General remarks

All chemicals were obtained from commercial suppliers and used without further purification unless otherwise noted. ^1H NMR spectra were recorded on Bruker AVANCE spectrometers (400 MHz) in $CDCl_3$ and DMSO-d_6 solution and ^{13}C NMR were recorded on Bruker AVANCE spectrometers (100 MHz) in $CDCl_3$ and DMSO-d_6 solution. Data for ^1H NMR spectra were reported as follows: chemical shift (δ ppm), multiplicity (br = broad, s = singlet, d = double, t = triplet, q = quartet, m = multiplet), coupling constant in Hertz (Hz) and integration were referenced to the residual solvent peak 7.26 ppm for $CDCl_3$ and 2.50 ppm for DMSO-d_6. ^{13}C NMR spectra were referenced to the residual solvent peak 77.16 ppm for $CDCl_3$ and 39.52 ppm for DMSO-d_6. Mass spectra were recorded on a Shimadzu GCMS-QP2010 Ultra and an HP 5989A mass selective detector with helium (He) as the carrier gas. High resolution mass spectrometry (HR-MS) was performed on a Bruker solariX XR instrument equipped with ParaCell ICR cell using positive electrospray ionization (ESI^+). The photoreactor and light source are designed by hsly-Green Lab (Shanghai, China).[1] Elemental analysis was conducted by Vario Macro CNS to detect the carbon content of samples. High-temperature gel permeation chromatography (HGPC) measurements were performed with Agilent PL-GPC220 with a PLgel 10 μm MIXED-B 50 x 7.5 mm and three PL gel 10 μm MIXED-B 300 x 7.5 mm, using 1-chloronaphthalene as eluent with a flow rate of 1.0 mL/min at 210 °C. The matrix-assisted laser desorption/ionization time-of-flight mass spectrometry (MALDI-TOF-MS) data were obtained using an AB SCIEX 4800 PLUS MALDI TOF/TOF instrument, with DCTB as the matrix, and acquired in reflection mode with a mass range of 200-2000 Da. Differential scanning calorimetry (DSC) and thermogravimetry analysis (TGA) were performed with a Discovery SDT 650. The samples were heated from room temperature to 300 °C under nitrogen atmosphere at a rate of 10 °C/min, then cooled down to room temperature at the same rate, held at room temperature for 1 min, and then heated again to 300 °C at the same rate to give the DSC data. The samples were heated from room temperature to 800 °C under nitrogen atmosphere at a rate of

20 °C/min to obtain the TGA curves. X-ray powder diffraction patterns (XRD) were collected on an Ultima IV diffractometer using monochromatic Cu Kα radiation (λ = 1.5418 Å), operating at 40 kV and 40 mA over a 2θ range from 2° to 50°. The scanning electron microscope (SEM) measurements were conducted on Zeiss Gemini SEM450. The water contact angles were characterized using a JC2000C instrument at room temperature. Infrared (IR) spectra were obtained using a Thermo Nicolet Nexus 670 spectrometer. The spectra were recorded from an accumulation of 16 scans in the range of 4000-400 cm^{-1} and were collected at room temperature. Electron paramagnetic resonance (EPR) spectroscopy data were collected on a Bruker A300. UV-vis absorption experiments were performed using a Shimadzu UV2700 UV-visible spectrophotometer.

The desulfurizing chlorination of aryl thioether models

(i) General procedures for the synthesis of aryl thioether models

Aryl thioethers **1a**, **1b**, **1f**, **1m**, **1r**, **1u**, **1v** and **1w** were purchased from Energy Chemical, and other sulfone models were prepared according to the reported literatures.[2-8]

The synthesis of thioethers **1c-1e**, **1h**, **1j**, **1l**, **1n-1q** and **1aa**[2]

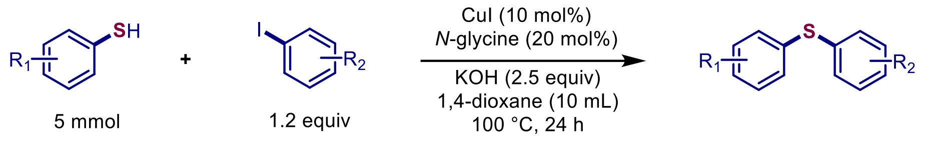

General procedure 1 (GP 1): A 50 mL Schlenk flask was charged with phenylthiophenol (5.0 mmol), iodobenzene (6.0 mmol), cuprous iodide (0.5 mmol, 10 mol%), *N*-methylglycine (1.0 mmol, 20 mol%), potassium hydroxide (12.5 mmol, 2.5 equiv) and 1,4-dioxane (10 mL) under N_2 atmosphere. The reaction mixture was then stirred at 100 °C for 24 h. After cooling to room temperature, the solvent was evaporated under reduced pressure. The residue was diluted with ethyl acetate (30 mL), washed with saturated aqueous NaCl solution (3 x 20 mL), dried over anhydrous Na_2SO_4, and concentrated in vacuum. Purification by column chromatography on

silica gel using petroleum ether (PE)/ethyl acetate (EA) as the eluent afforded the corresponding product.

Selected characterization data

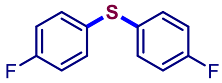

Bis(4-fluorophenyl)sulfane (1c): The title compound was isolated (766.0 mg, 69%) according to GP 1 as a colorless oil after chromatography on silica gel (PE/EA = 50:1). ^{1}H NMR (400 MHz, $CDCl_3$): δ 7.33-7.30 (m, 4H), 7.04-7.00 (m, 4H). ^{13}C NMR (100 MHz, $CDCl_3$): δ 162.3 (d, J_{CF} = 246.0 Hz), 133.1 (d, J_{CF} = 8.0 Hz), 131.2 (d, J_{CF} = 3.3 Hz), 116.5 (d, J_{CF} = 21.9 Hz). ^{19}F NMR (376 MHz, $CDCl_3$): δ -114.3. GC-MS (EI$^+$): calcd for $C_{12}H_8F_2S$ $[M]^+$ 222.03, found 222.05.

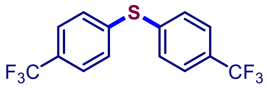

Bis(4-(trifluoromethyl)phenyl)sulfane (1d): The title compound was isolated (1207.8 mg, 75%) according to GP 1 as a colorless oil after chromatography on silica gel (PE/EA = 50:1). ^{1}H NMR (400 MHz, $CDCl_3$): δ 7.59 (d, J = 8.4 Hz, 4H), 7.45 (d, J = 8.4 Hz, 4H). ^{13}C NMR (100 MHz, $CDCl_3$): δ 139.6, 131.2, 129.8 (q, J_{CF} = 32.7 Hz), 126.4 (q, J_{CF} = 3.7 Hz), 124.0 (q, J_{CF} = 270.2 Hz). ^{19}F NMR (376 MHz, $CDCl_3$): δ -62.7. GC-MS (EI$^+$): calcd for $C_{14}H_8F_6S$ $[M]^+$ 322.03, found 322.05.

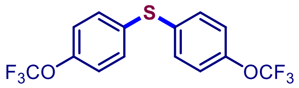

Bis(4-(trifluoromethoxy)phenyl)sulfane (1e): The title compound was isolated (1102.7 mg, 62%) according to GP 1 as a colorless oil after chromatography on silica gel (PE/EA = 50:1). ^{1}H NMR (400 MHz, $CDCl_3$): δ 7.36 (d, J = 8.8 Hz, 4H), 7.17 (d, J = 8.0 Hz, 4H). ^{13}C NMR (100 MHz, $CDCl_3$): δ 148.7 (d, J_{CF} = 1.9 Hz), 134.1, 132.6, 122.0, 120.5 (q, J_{CF} = 256.0 Hz). ^{19}F NMR (376 MHz, $CDCl_3$): δ -57.9. GC-MS (EI$^+$):

calcd for $C_{14}H_8F_6O_2S$ $[M]^+$ 354.01, found 354.00.

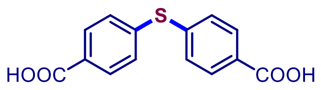

4,4'-Thiodibenzoic acid (1g)[3]: A 50 ml Schlenk bottle was charged with 4-iodobenzoic acid (10.0 mmol), sodium sulfide nonahydrate (5.5 mmol), cuprous iodide (1 mmol) and potassium carbonate (5 mmol), which was then evacuated/backfilled with nitrogen for three times followed by the addition of *N*,*N*-dimethylformamide (10 mL) *via* syringe. The reaction mixture was stirring at 120 °C for 24 hours. After cooling to room temperature, water (100 mL) was then added, and the mixture was filtered into HCl solution (36.0~38.0%, 50 mL). The precipitate formed on cooling to room temperature is filtered, washed with water (20 mL) for three times, and dried in an oven. The title compound was gained (1208.3 mg, 88%) as a white solid. ^{1}H NMR (400 MHz, DMSO-d_6): δ 13.13 (s, 2H), 7.87 (d, J = 8.0 Hz, 4H), 7.69 (d, J = 8.0 Hz, 4H). ^{13}C NMR (100 MHz, DMSO-d_6): δ 166.9, 137.6, 131.1, 130.3, 101.2. GC-MS (EI$^+$): calcd for $C_{14}H_{10}O_4S$ $[M]^+$ 274.03, found 274.05.

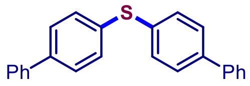

Di([1,1'-biphenyl]-4-yl)sulfane (1h): The title compound was isolated (1102.7 mg, 48%) according to GP1 as a white solid after chromatography on silica gel (PE/EA = 50:1). ^{1}H NMR (400 MHz, $CDCl_3$): δ 7.61-7.56 (m, 8H), 7.48-7.44 (m, 8H), 7.39-7.35 (m, 2H). ^{13}C NMR (100 MHz, $CDCl_3$): δ 140.4, 140.2, 134.9, 131.5, 129.0, 128.0, 127.6, 127.1. GC-MS (EI$^+$): calcd for $C_{24}H_{18}S$ $[M]^+$ 338.11, found 338.10.

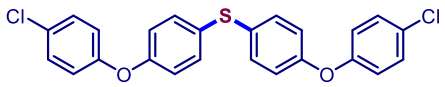

Bis(4-(4-chlorophenoxy)phenyl)sulfane (1i)[4]: A 50 ml Schlenk bottle was charged with 4,4'-thiodiphenol (5.0 mmol), 1-bromo-4-chlorobenzene (12.0 mmol), copper iodide (0.5 mmol, 10 mol%), 2-pyridinecarboxylic acid (0.5 mmol, 10 mol%), and

K_3PO_4 (10.0 mmol), which was then evacuated/backfilled with nitrogen for three times followed by the addition of dimethyl sulfoxide (20 mL) *via* syringe. The reaction mixture was stirred at 120 °C for 48 h. After cooling to room temperature, the mixture was diluted with ethyl acetate (100 mL), washed with saturated NaCl (3 x 100 mL) for three times, dried with anhydrous Na_2SO_4, and then concentrated in vacuum. The title compound was isolated (1842.0 mg, 84%) as a white solid after chromatography on silica gel (PE/EA = 50:1 to 20:1). ^{1}H NMR (400 MHz, $CDCl_3$): δ 7.33-7.29 (m, 8H), 6.96-6.92 (m, 8H). ^{13}C NMR (100 MHz, $CDCl_3$): δ 156.6, 155.5, 132.9, 130.4, 130.0, 128.9, 120.5, 119.6. GC-MS (EI$^+$): calcd for $C_{24}H_{16}Cl_2O_4S$ $[M]^+$ 438.02, found 438.05.

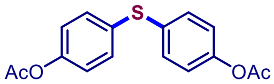

Thiobis(4,1-phenylene) diacetate (1j): The title compound was isolated (890.9 mg, 60%) according to GP1 as a white solid after chromatography on silica gel (PE/EA = 10:1). ^{1}H NMR (400 MHz, $CDCl_3$): δ 7.35 (d, J = 8.8 Hz, 4H), 7.04 (d, J = 8.8 Hz, 4H), 2.30 (s, 6H). ^{13}C NMR (100 MHz, $CDCl_3$): δ 169.4, 150.0, 133.0, 132.3, 122.6, 21.2. GC-MS (EI$^+$): calcd for $C_{16}H_{14}O_4S$ $[M]^+$ 302.06, found 302.05.

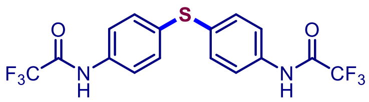

***N*,*N*'-(Thiobis(4,1-phenylene))bis(2,2,2-trifluoroacetamide) (1k)[5]:** A 50 mL Schlenk bottle was charged with 4,4'-thiodianiline (5.0 mmol), trifluoroacetic anhydride (12.0 mmol), and dichloromethane (15 mL). The reaction mixture was stirred at room temperature for 12 hours, monitored by TLC until the reaction was complete. The mixture was diluted with dichloromethane (20 mL), washed with saturated NaCl (3*20 mL) for three times, dried with anhydrous Na_2SO_4, and then concentrated in vacuum. The title compound was isolated (890.9 mg, 88%) as a yellow solid after chromatography on silica gel (PE/EA = 50:1 to 5:1). ^{1}H NMR (400 MHz, DMSO-d_6): δ 11.37 (s, 2H), 7.69 (d, J = 8.8 Hz, 4H), 7.36 (d, J = 8.8 Hz, 4H).

^{13}C NMR (100 MHz, DMSO-d_6): δ 155.0 (q, J_{CF} = 36.8 Hz), 136.2, 132.0, 131.9, 122.4, 116.2 (q, J_{CF} = 286.9 Hz). ^{19}F NMR (376 MHz, $CDCl_3$): δ -73.9. GC-MS (EI^+): calcd for $C_{16}H_{10}F_6N_2O_2S$ $[M]^+$ 408.04, found 408.05.

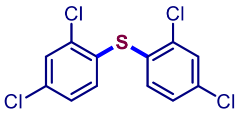

Bis(2,4-dichlorophenyl)sulfane (1i): The title compound was isolated (777.2 mg, 48%) according to GP1 as a yellow thick oil after chromatography on silica gel (PE/EA = 50:1). ^1H NMR (400 MHz, $CDCl_3$): δ 7.48 (d, J = 2.0 Hz, 2H), 7.19 (dd, J = 8.8 Hz, 2.4 Hz, 2H), 7.07 (d, J = 8.4 Hz, 2H). ^{13}C NMR (100 MHz, $CDCl_3$): δ 136.4, 134.5, 133.3, 131.6, 130.3, 128.1. HR-MS (ESI^+): calcd for $C_{12}H_7Cl_4S$ $[M+H]^+$ 322.9017, found 322.9011.

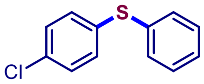

(4-Chlorophenyl)(phenyl)sulfane (1n): The title compound was isolated (714.8 mg, 65%) according to GP1 as a colorless oil after chromatography on silica gel (PE/EA = 50:1). ^1H NMR (400 MHz, $CDCl_3$): δ 7.36-7.30 (m, 4H), 7.29-7.24 (m, 5H). ^{13}C NMR (100 MHz, $CDCl_3$): δ 135.3, 134.8, 133.2, 132.2, 131.5, 129.5, 129.5, 127.6. GC-MS (EI^+): calcd for $C_{12}H_9ClS$ $[M]^+$ 220.01, found 220.00.

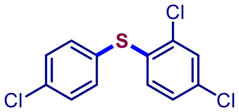

(4-Chlorophenyl)(2,4-dichlorophenyl)sulfane (1o): The title compound was isolated (806.0 mg, 56%) according to GP1 as a white solid after chromatography on silica gel (PE/EA = 50:1). ^1H NMR (400 MHz, $CDCl_3$): δ 7.42 (d, J = 2.0 Hz, 1H), 7.37-7.32 (m, 4H), 7.12 (dd, J = 8.4 Hz, 2.0 Hz, 1H), 6.94 (d, J = 8.4 Hz, 1H). ^{13}C NMR (100 MHz, $CDCl_3$): δ 134.9, 134.6, 134.4, 134.3, 134.1, 131.4, 131.2, 130.0, 129.8, 127.8. HR-MS (ESI^+): calcd for $C_{12}H_8Cl_3S$ $[M+H]^+$ 288.9407, found 288.9407.

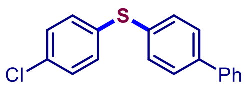

[1,1'-Biphenyl]-4-yl(4-chlorophenyl)sulfane (1p): The title compound was isolated (561.8 mg, 38%) according to GP1 as a white solid after chromatography on silica gel (PE/EA = 50:1 to 10:1). ^{1}H NMR (400 MHz, $CDCl_3$): δ 7.59 (d, J = 7.6 Hz, 2H), 7.56 (d, J = 8.4 Hz, 2H), 7.46 (t, J = 7.6 Hz, 2H), 7.41 (d, J = 8.0 Hz, 2H), 7.37 (t, J = 7.2 Hz, 1H), 7.30 (s, 4H). ^{13}C NMR (100 MHz, $CDCl_3$): δ 140.5, 140.3, 134.7, 134.3, 133.2, 132.2, 131.7, 129.5, 129.0, 128.1, 127.7, 127.1. GC-MS (EI$^+$): calcd for $C_{18}H_{13}ClS$ [M]$^+$ 296.04, found 296.00.

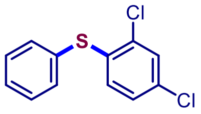

(2,4-Dichlorophenyl)(phenyl)sulfane (1q): The title compound was isolated (905.5 mg, 70%) according to GP1 as a colorless oil after chromatography on silica gel (PE/EA = 50:1). ^{1}H NMR (400 MHz, $CDCl_3$): δ 7.45-7.38 (m, 6H), 7.08 (dd, J = 8.4 Hz, 2.0 Hz, 1H), 6.88 (d, J = 8.4 Hz, 1H). ^{13}C NMR (100 MHz, $CDCl_3$): δ 135.5, 133.7, 133.4, 132.4, 132.2, 130.7, 129.8, 129.5, 128.7, 127.5. GC-MS (EI$^+$): calcd for $C_{12}H_8Cl_2S$ [M]$^+$ 253.97, found 254.00.

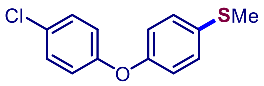

(4-(4-Chlorophenoxy)phenyl)(methyl)sulfane (1s)[4]: A 50 ml Schlenk bottle was charged with 4-chlorophenol (5.0 mmol), 4-methylthiobromobenzene (6.0 mmol), copper iodide (0.5 mmol, 10 mol%), 2-pyridinecarboxylic acid (0.5 mmol, 10 mol%), K_3PO_4 (10.0 mmol) and DMSO (20 mL) under the nitrogen atmosphere. The reaction mixture was stirred at 120 °C for 48 h. After cooling to room temperature, the mixture was diluted with ethyl acetate (100 mL), washed with saturated NaCl (3*100 mL) for three times, dried with anhydrous Na_2SO_4, and then concentrated in vacuum. The title compound was isolated (662.5 mg, 53%) as a white solid after chromatography on silica gel (PE/EA = 50:1 to 10:1). ^{1}H NMR (400 MHz, $CDCl_3$): δ 7.35-7.32 (m, 4H),

7.01-6.97 (m, 4H), 2.54 (s, 3H). ^{13}C NMR (100 MHz, $CDCl_3$): δ 156.1, 154.9, 133.1, 129.9, 129.2, 128.3, 119.9, 119.8, 17.1. GC-MS (EI^+): calcd for $C_{13}H_{11}ClOS$ $[M]^+$ 250.02, found 250.00.

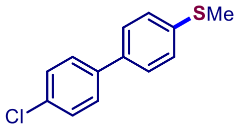

(4'-Chloro-[1,1'-biphenyl]-4-yl)(methyl)sulfane (1t)[6]: A 50 ml Schlenk bottle was charged with 1-bromo-4-chlorobenzene (5.0 mmol), (4-(methylthio)phenyl)boronic acid (6.0 mmol), Pd(dppf)Cl_2 (0.2 mmol, 4 mol%), and K_3PO_4 (15.0 mmol). After evacuated/backfilled with nitrogen for three times, H_2O (25.0 mmol) and 1,4-dioxane (20 mL) was added *via* syringe. The reaction mixture was stirred at 80 °C for 12 hours, and monitored by TLC until the reaction was complete. After cooling to room temperature, the solvent with saturated NaCl (3*20 mL) for three times, dried with anhydrous Na_2SO_4, and concentrated in vacuum. The title compound was isolated (830.7 mg, 71%) as a white solid after chromatography on silica gel (PE/EA = 50:1 to 10:1). ^{1}H NMR (400 MHz, $CDCl_3$): δ 7.50-7.47 (m, 4H), 7.40 (d, J = 8.4 Hz, 2H), 7.32 (d, J = 8.4 Hz, 2H), 2.53 (s, 3H). ^{13}C NMR (100 MHz, $CDCl_3$): δ 139.1, 138.3, 136.8, 133.4, 129.1, 128.2, 127.4, 127.1, 15.9. GC-MS (EI^+): calcd for $C_{13}H_{11}ClS$ $[M]^+$ 234.03, found 233.95.

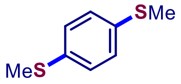

1,4-bis(methylthio)benzene (1x)[7]: A 50 mL Schlenk bottle was charged with (4-iodophenyl)(methyl)sulfane (5.0 mmol), cupric acetate (10.0 mmol), cuprous iodide (1.25 mmol), and dimethyl sulfoxide (20 mL). The reaction mixture was stirred at 135 °C for 36 hours, monitored by TLC until the reaction was complete. the mixture was diluted with ethyl acetate (100 mL), washed with saturated NaCl (3*100 mL) for

three times, dried with anhydrous Na_2SO_4, and then concentrated in vacuum. The title compound was isolated (742.8 mg, 42%) as a yellow solid after chromatography on silica gel (PE/EA = 50:1 to 10:1). ^1H NMR (400 MHz, $CDCl_3$): δ 7.20 (s, 4H), 2.46 (s, 6H). ^{13}C NMR (100 MHz, $CDCl_3$): δ 135.2, 127.6, 16.4. GC-MS (EI^+): calcd for $C_8H_{10}S_2$ $[M]^+$ 170.02, found 169.95.

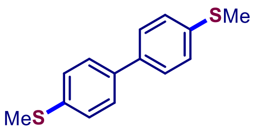

4,4'-Bis(methylthio)-1,1'-biphenyl (1y)[6]: A 50 ml Schlenk bottle was charged with (4-bromophenyl)(methyl)sulfane (5.0 mmol), (4-(methylthio)phenyl)boronic acid (6.0 mmol), Pd(dppf)Cl_2 (0.2 mmol, 4 mol%), and K_3PO_4 (15.0 mmol). After evacuated/backfilled with nitrogen for three times, H_2O (25.0 mmol) and 1,4-dioxane (20 mL) was added *via* syringe. The reaction mixture was stirred at 80 °C for 12 hours, and monitored by TLC until the reaction was complete. After cooling to room temperature, the solvent with saturated NaCl (3*20 mL) for three times, dried with anhydrous Na_2SO_4, and concentrated in vacuum. The title compound was isolated (787.2 mg, 64%) as a white solid after chromatography on silica gel (PE/EA = 50:1 to 10:1). ^1H NMR (400 MHz, $CDCl_3$): δ 7.50 (d, J = 8.8 Hz, 4H), 7.32 (d, J = 8.8 Hz, 4H), 2.52 (s, 6H). ^{13}C NMR (100 MHz, $CDCl_3$): δ 137.7, 137.5, 127.3, 127.1, 16.1. GC-MS (EI^+): calcd for $C_{14}H_{14}S_2$ $[M]^+$ 246.05, found 246.00.

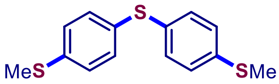

Bis(4-(methylthio)phenyl)sulfane (1z)[8]: A 50 mL Schlenk bottle was charged with 4,4'-thiodibenzenethiol (5.0 mmol), tetrabutylammonium iodide (10.0 mmol), potassium carbonate (10.0 mmol), and *N*,*N*-dimethylformamide (20 mL), followed by the addition of methyl iodide (12.0 mmol) *via* syringe. The reaction mixture was stirred at room temperature for 12 hours, monitored by TLC until the reaction was complete. the mixture was diluted with ethyl acetate (100 mL), washed with saturated

NaCl (3*100 mL) for three times, dried with anhydrous Na_2SO_4, and then concentrated in vacuum. The title compound was isolated (890.9 mg, 55%) as a yellow solid after chromatography on silica gel (PE/EA = 50:1 to 5:1). ^{1}H NMR (400 MHz, $CDCl_3$): δ 7.26 (s, 8H), 2.47 (s, 6H). ^{13}C NMR (100 MHz, $CDCl_3$): δ 138.0, 132.4, 131.6, 127.4, 15.97. GC-MS (EI$^+$): calcd for $C_{14}H_{14}S_3$ $[M]^+$ 278.03, found 278.05.

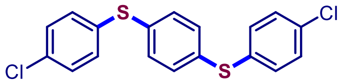

1,4-Bis((4-chlorophenyl)thio)benzene (1aa): The title compound was isolated (561.8 mg, 44%) according to GP1 (with 10.0 mmol 4-chlorobenzenethiol and 6.0 mmol 1,4-diiodobenzene) as a yellow solid after chromatography on silica gel (PE/EA = 50:1 to 5:1). ^{1}H NMR (400 MHz, $CDCl_3$): δ 7.28 (s, 8H), 7.22 (s, 4H). ^{13}C NMR (100 MHz, $CDCl_3$): δ 134.9, 133.7, 133.7, 132.8, 131.4, 129.6. GC-MS (EI$^+$): calcd for $C_{18}H_{12}Cl_2S_2$ $[M]^+$ 361.98, found 362.00.

(ii) Optimization for the desulfurizing chlorination of aryl thioether

A 4 mL screw-capped vial with a stirring bar were added **1a** (0.1 mmol), catalyst (10 mol%), solvent, and a chlorinated alkane (2-10 equiv). The vial was then sealed with a cap containing a PTFE septum and linked to an oxygen or nitrogen balloon (spherical diameter: ~25 cm). The reaction mixture was stirred under the irradiation of blue LEDs (400-405 nm, 12 W) at 25-30 ºC for 6-24 h (**Figure S1**). After reaction, internal standard **2b** (0.2 mmol) was added to the reaction mixture, and then 5 μL of the solution was drawn, diluted immediately with ethyl acetate, and measured by GC-MS analysis to determine the yields, which were calculated according to the equation (A = Amount of substance, P = peak area): (i) $A_{\mathbf{2a/2b}} = 0.5580*P_{\mathbf{2a/2b}}+0.0023$, $R^2 = 0.9989$. The selected results were summarized in **Table S1**.

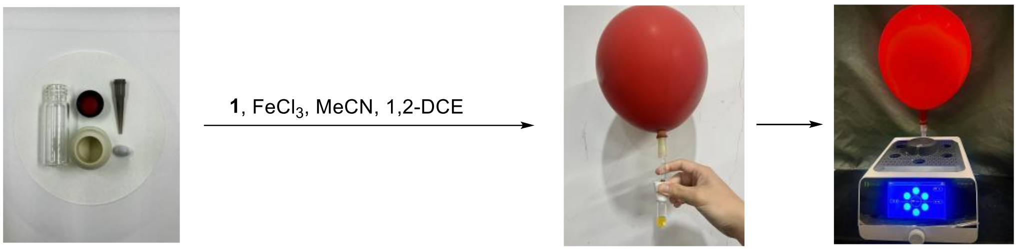

Figure S1. Schematic diagram of the reaction

Table S1. The optimization for the desulfurizing chlorination of **1a**

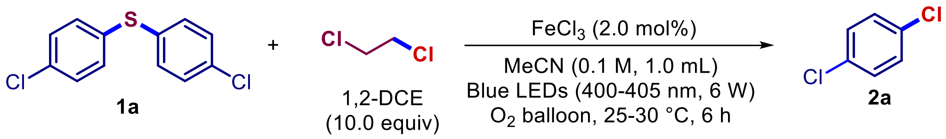

Entry	Variation from the standard conditions	Yield of **2a** (%)
1	**None**	**86% (78%)**
2	Dark	nd.
3	No $FeCl_3$	nd.
4	N_2 atmosphere	nd.
5	$CuCl_2$ instead of $FeCl_3$	18
6	$CeCl_3$ instead of $FeCl_3$	12
7	$BiCl_3$ instead of $FeCl_3$	nd.
8	5 equiv 1,2-DCE	39
9	2 equiv 1,2-DCE	20
10	1,2-DCE as the solvent	49
11	DCM or $CHCl_3$ instead of 1,2-DCE	<10%
12	430-435 nm instead of 400-405 nm	53
13	455-460 nm instead of 400-405 nm	trace

GC yields and isolated yields in parentheses of **2a** were calculated on molar basis of aryl rings

For the reaction in Entry 1, 5 μL of the solution was drawn, diluted immediately with toluene, and measured by GC-MS analysis. The formation of chloroacetic acid (**3**) was detected, which was further confirmed by ^{1}H NMR analysis of the reaction mixture (**Figure S2-3**).

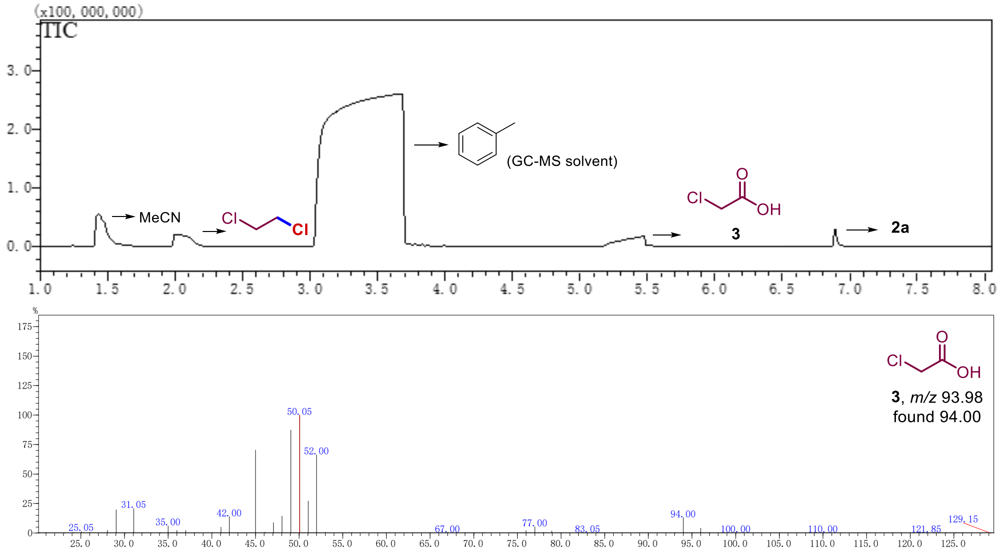

Figure S2. GC-MS detection of **3** and **9** after reaction of **1a** for 24 h

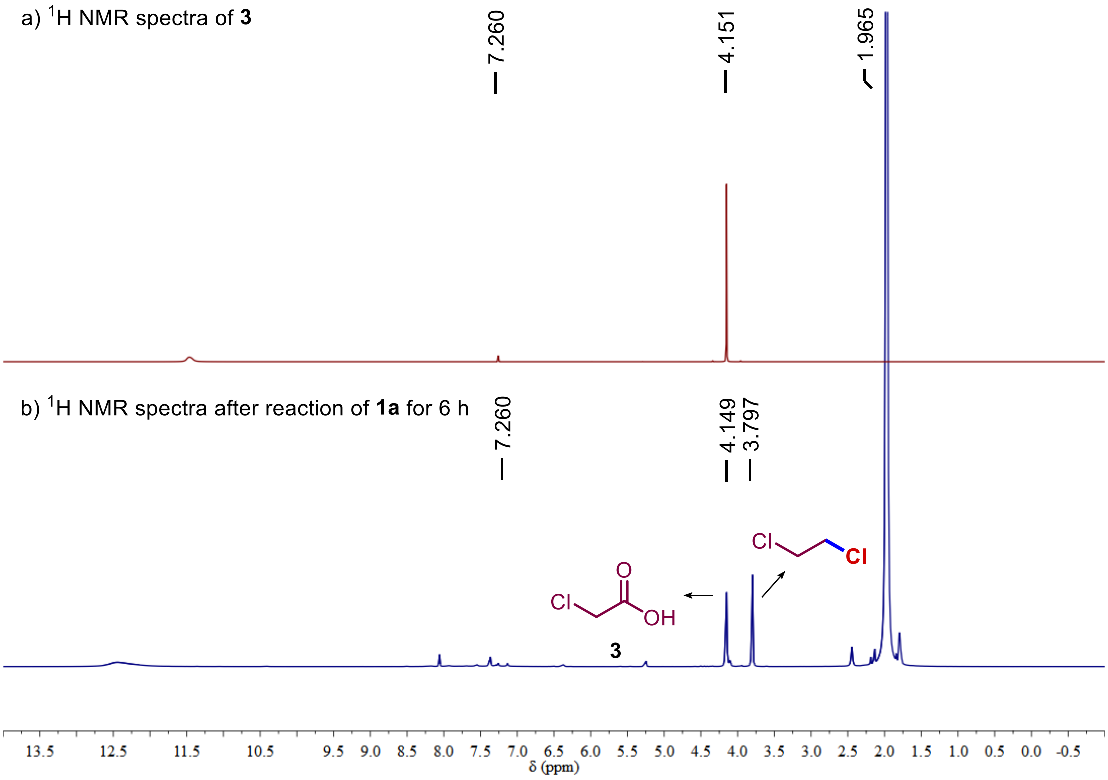

Figure S3. ^{1}H NMR detection of **3** after reaction of **1a** for 24 h

(iii) General procedure for the desulfurizing chlorination of aryl thioethers (GP2)

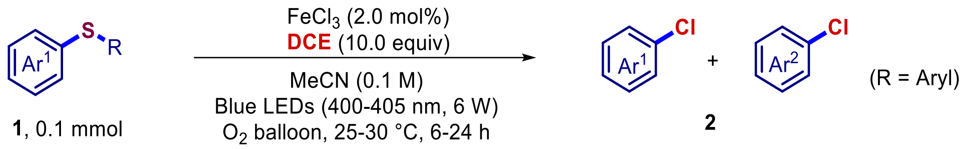

A 4 mL screw-capped vial with a stirring bar were added **1** (0.1 mmol), $FeCl_3$ (2 mol%, 0.02 M in CH_3CN solution, 1.0 mL), and 1,2-DCE (1 mmol, 10 equiv, 0.08 mL) *[Note: For multiple thioethers, 4 mol% of $FeCl_3$ and 2 mmol of 1,2-DCE were added]*. The vial was then sealed with a cap containing a PTFE septum and linked to an oxygen balloon (spherical diameter: ~25 cm). The reaction mixture was stirred under the irradiation of blue LEDs (400-405 nm, 12 W) at 25-30 ºC for 6-24 h (**Figure S1**). The resulting reaction mixture was concentrated in vacuum and the residue was purified by flash chromatography on silica gel column to provide the product **2**. For cases yielding volatile aryl chlorides, an internal standard (0.1 mmol or 0.2 mmol) was added to the reaction mixture, and then 5 μL of the solution was drawn, diluted immediately with ethyl acetate, and measured by GC-MS analysis to determine the yields. For cases affording volatile fluorinated aryl chlorides, an internal standard (0.1 mmol or 0.2 mmol) was added to the reaction mixture and 100 μL was taken, diluted with $CDCl_3$ and measured by ^{19}F NMR (376 MHz). See **Figure S4-S16** and **Figure S17-S19** for details.

Selected characterization data

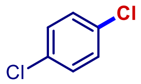

1,4-Dichlorobenzene (2a): The title compound was isolated (22.6 mg, 78%) from reaction of **1a** reacted for 6 h according to GP2 as a white solid after chromatography on silica gel (pure PE). ^1H NMR (400 MHz, $CDCl_3$): δ 7.27 (s, 4H). ^{13}C NMR (100 MHz, $CDCl_3$): δ 132.7, 130.0. GC-MS (EI^+): calcd for $C_6H_4Cl_2$ $[M]^+$ 145.97, found 146.00.

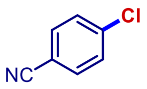

4-Chlorobenzonitrile (2f): The title compound was isolated (16.5 mg, 60%) from reaction of **1f** reacted for 6 h according to GP2 as a white solid after chromatography on silica gel (PE/EA = 100:1). ^{1}H NMR (400 MHz, $CDCl_3$): δ 7.60 (d, J = 8.8 Hz, 2H), 7.47 (d, J = 8.8 Hz, 2H). ^{13}C NMR (100 MHz, $CDCl_3$): δ 139.7, 133.5, 129.8, 118.1, 110.9. GC-MS (EI$^+$): calcd for C_7H_4ClN $[M]^+$ 137.00, found 137.00.

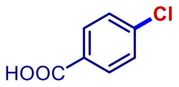

4-**Chlorobenzoic acid (2g):** The title compound was isolated (22.8 mg, 73%) from reaction of **1g** reacted for 6 h according to GP2 as a white solid after chromatography on silica gel (PE/EA = 5:1). ^{1}H NMR (400 MHz, DMSO-d_6): δ 13.18 (br, s, 1H), 7.93 (d, J = 8.4 Hz, 2H), 7.55 (d, J = 8.4 Hz, 2H). ^{13}C NMR (100 MHz, DMSO-d_6): δ 166.5, 137.9, 131.2, 129.7, 128.8. GC-MS (EI$^+$): calcd for $C_7H_5ClO_2$ $[M]^+$ 156.00, found 156.00.

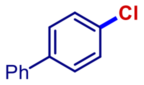

4-Chloro-1,1'-biphenyl (2h): The title compound was isolated in 55% yield (20.7 mg) from reaction of **1h** reacted for 6 h, and 66% yield (12.4 mg) from reaction of **1p** reacted for 6 h according to GP2 as a white solid after chromatography on silica gel (pure PE). ^{1}H NMR (400 MHz, $CDCl_3$): δ 7.58-7.52 (m, 4H), 7.48-7.36 (m, 5H). ^{13}C NMR (100 MHz, $CDCl_3$): δ 140.1, 139.8, 133.5, 129.1, 129.0, 128.5, 127.7, 127.1. GC-MS (EI$^+$): calcd for $C_{12}H_9Cl$ $[M]^+$ 188.04, found 188.00.

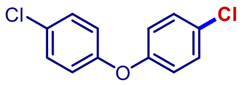

4,4'-Oxybis(chlorobenzene) (2i): The title compound was isolated in 70% yield (33.3 mg) from reaction of **1i** reacted for 24 h, and 78% yield (18.5 mg) from reaction of **1s** reacted for 6 h according to GP2 as a colorless oil after chromatography on silica gel (PE/EA = 50:1). ^{1}H NMR (400 MHz, $CDCl_3$): δ 7.30 (d, J = 8.8 Hz, 4H), 6.93 (d, J = 8.8 Hz, 4H). ^{13}C NMR (100 MHz, $CDCl_3$): δ 155.7, 130.0, 128.8, 120.2. GC-MS (EI$^+$): calcd for $C_{12}H_8Cl_2O$ $[M]^+$ 238.00, found 238.00.

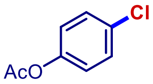

4-Chlorophenyl acetate (2j): The title compound was isolated (17.7 mg, 52%) from reaction of **1j** reacted for 6 h according to GP2 as a white solid after chromatography on silica gel (PE/EA = 15:1). ^{1}H NMR (400 MHz, $CDCl_3$): δ 7.34 (d, J = 8.8 Hz, 2H), 7.03 (d, J = 8.8 Hz, 2H), 2.29 (s, 3H). ^{13}C NMR (100 MHz, $CDCl_3$): δ 169.3, 149.2, 131.3, 129.6, 123.1, 21.2. GC-MS (EI$^+$): calcd for $C_8H_7ClO_2$ $[M]^+$ 170.01, found 170.00.

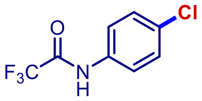

***N*-(4-chlorophenyl)-2,2,2-trifluoroacetamide (2k):** The title compound was isolated (27.7 mg, 62%) after reaction of **1k** for 6 h according to GP2 as a white solid after chromatography on silica gel (PE/EA = 10:1). ^{1}H NMR (400 MHz, $CDCl_3$): δ 8.05 (s, 1H), 7.52 (d, J = 8.4 Hz, 2H), 7.36 (d, J = 8.4 Hz, 2H). ^{13}C NMR (100 MHz, $CDCl_3$): δ 155.0 (q, J_{CF} = 37.5 Hz), 133.7, 131.9, 129.6, 121.9, 115.7 (q, J_{CF} = 286.7 Hz). ^{19}F NMR (376 MHz, $CDCl_3$): δ -75.7. GC-MS (EI$^+$): calcd for $C_8H_5ClF_3NO$ $[M]^+$ 223.00, found 222.95.

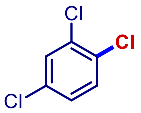

1,2,4-Trichlorobenzene (2l): The title compound was isolated (32.4 mg, 90%) from reaction of **1l** reacted for 6 h according to GP2 as a colorless oil after chromatography

on silica gel (PE/EA = 100:1). ^{1}H NMR (400 MHz, $CDCl_3$): δ 7.46 (d, J = 2.4 Hz, 1H), 7.37 (d, J = 8.4 Hz, 1H), 7.19 (dd, J = 8.4, 2.4 Hz, 1H). ^{13}C NMR (100 MHz, $CDCl_3$): δ 133.5, 133.1, 131.2, 130.4, 128.1. GC-MS (EI$^+$): calcd for $C_6H_3Cl_3$ $[M]^+$ 179.93, found 179.95.

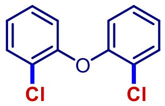

2,2'-Oxybis(chlorobenzene) (2m): The title compound was isolated (19.0 mg, 80%) from reaction of **1m** reacted for 6 h according to GP2 as a colorless oil after chromatography on silica gel (PE/EA = 50:1). ^{1}H NMR (400 MHz, $CDCl_3$): δ 7.47 (d, J = 7.6 Hz, 2H), 7.21 (t, J = 7.6 Hz, 2H), 7.09 (t, J = 7.6 Hz, 2H), 6.87 (d, J = 8.4 Hz, 2H). ^{13}C NMR (100 MHz, $CDCl_3$): δ 152.4, 131.0, 128.1, 125.3, 124.9, 119.7. GC-MS (EI$^+$): calcd for $C_{12}H_8Cl_2O$ $[M]^+$ 238.00, found 238.00.

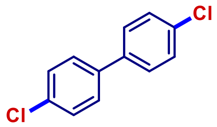

4,4'-Dichloro-1,1'-biphenyl (2t): The title compound was isolated in 81% yield (17.9 mg) from reaction of **1t** reacted for 6 h, and 66% yield (14.7 mg) from reaction of **1y** reacted for 6 h according to GP2 as a white solid after chromatography on silica gel (PE/EA = 50:1). ^{1}H NMR (400 MHz, $CDCl_3$): δ 7.48 (d, J = 8.4 Hz, 4H), 7.41 (d, J = 8.4 Hz, 4H). ^{13}C NMR (100 MHz, $CDCl_3$): δ 138.6, 133.9, 129.2, 128.4. GC-MS (EI$^+$): calcd for $C_{12}H_8Cl_2$ $[M]^+$ 222.00, found 221.95.

Selected GC-MS data for the volatile aryl chlorides

The GC yields for volatile aryl chlorides were calculated according to the equation (A = Amount of substance, P = peak area): (i) $A_{\mathbf{2a/2b}}$ = 0.5580*$P_{\mathbf{2a/2b}}$+0.0023, R^2 = 0.9989, (ii) $A_{\mathbf{2b/2l}}$ = 2.1451*$P_{\mathbf{2b/2l}}$-0.0146, R^2 = 0.9993, (iii) $A_{\mathbf{2a/2l}}$ = 1.3864*$P_{\mathbf{2a/2l}}$-0.1164, R^2 = 0.9997.

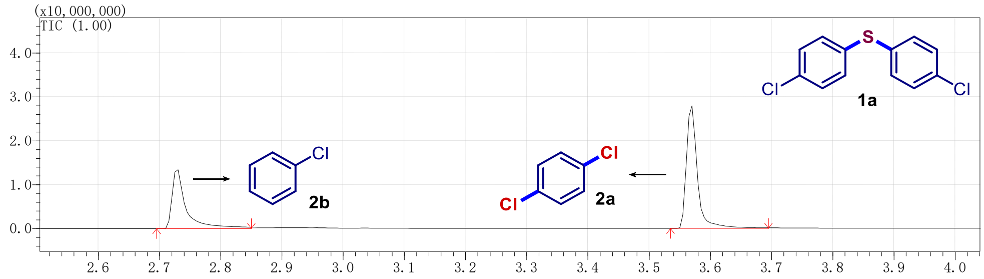

	Peak time	Peak area	Peak area (%)	Yield (%)
2b	2.728	21640768	39.53	100
**2a**	3.569	33104408	60.47	86

Figure S4. GC spectra of **2b** (0.2 mmol), and **2a** from **1a** (0.1 mmol)

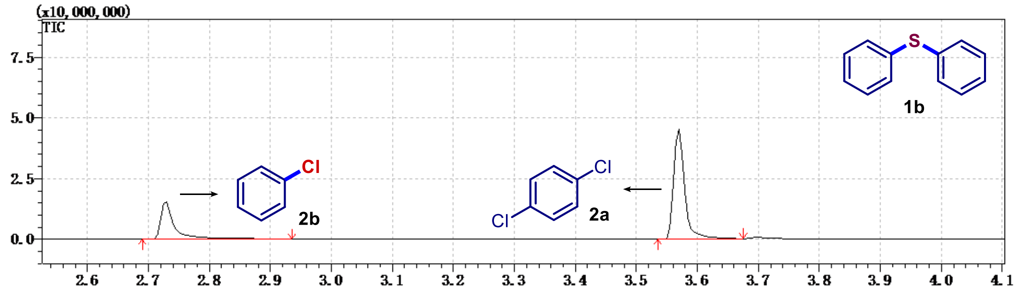

	Peak time	Peak area	Peak area (%)	Yield (%)
**2b**	2.730	25026557	34.07	93
**2a**	3.568	48429730	65.93	100

Figure S5. GC spectra of **2a** (0.2 mmol), and **2b** from **1b** (0.1 mmol)

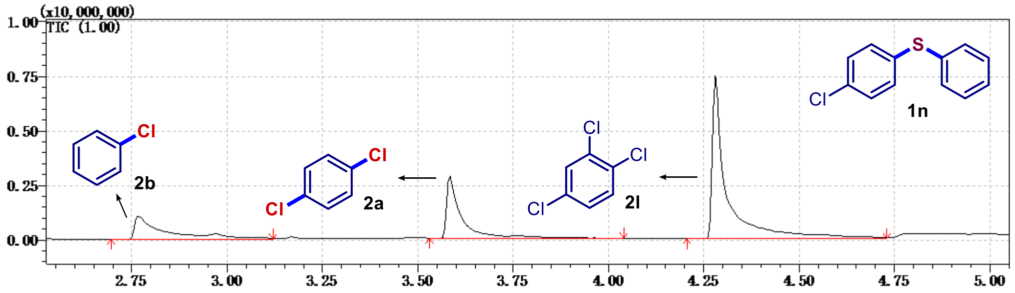

	Peak time	Peak area	Peak area (%)	Yield (%)
**2b**	2.767	5956647	16.17	67
**2a**	3.584	12027491	32.65	54
**2l**	4.281	18853506	51.18	100

Figure S6. GC spectra of **1n** (0.1 mmol), and **2a** & **2b** from **2l** (0.1 mmol)

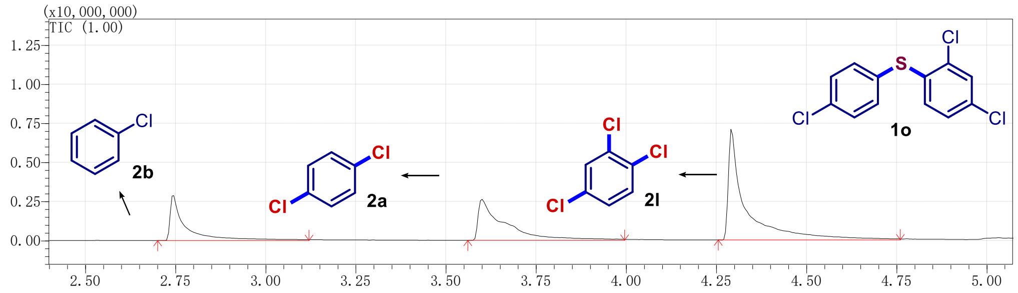

	Peak time	Peak area	Peak area (%)	Yield (%)
**2b**	2.743	8916054	23.04	100
**2a**	3.599	11907421	30.77	75
**2l**	4.292	17874676	46.19	94

Figure S7. GC spectra of **2b** (0.1 mmol), and **2a** & **2l** from **1o** (0.1 mmol)

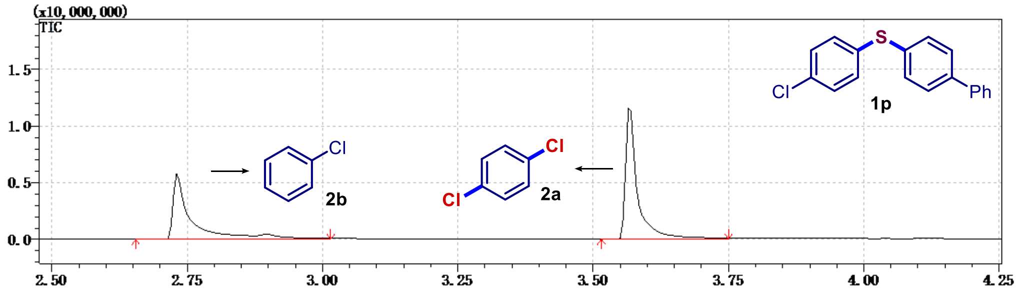

	Peak time	Peak area	Peak area (%)	Yield (%)
**2b**	2.731	13795656	44.10	100
**2a**	3.561	17487011	55.90	69

Figure S8. GC spectra of **2b** (0.1 mmol), and **2a** from **1p** (0.1 mmol)

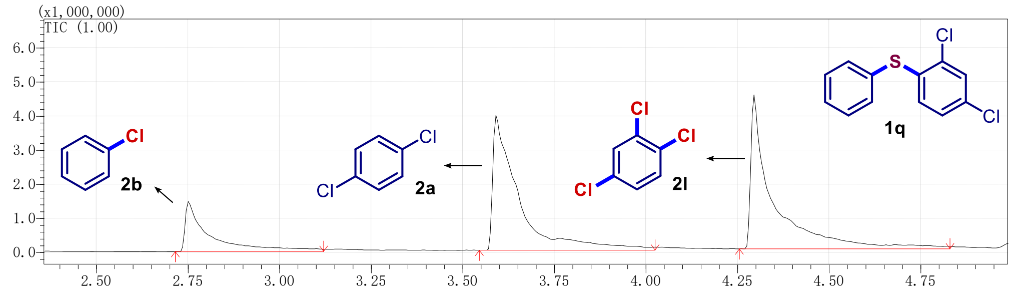

	Peak time	Peak area	Peak area (%)	Yield (%)
**2b**	2.751	6814975	19.72	75
**2a**	3.591	15976486	46.23	100
**2l**	4.295	11732678	33.95	89

Figure S9. GC spectra of **2a** (0.1 mmol), and **2b** & **2l** from **1q** (0.1 mmol)

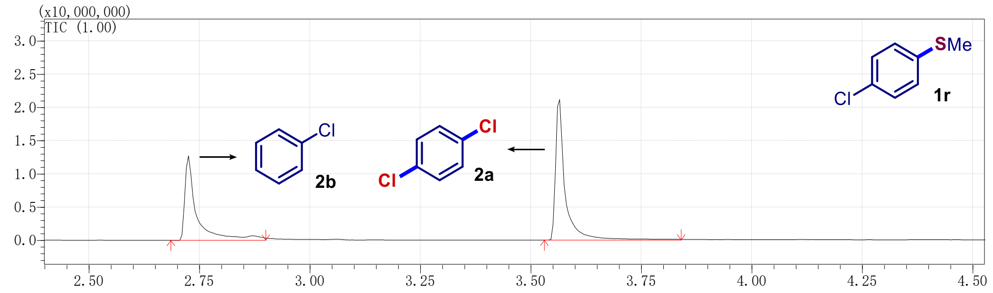

	Peak time	Peak area	Peak area (%)	Yield (%)
**2b**	2.725	24579326	41.18	100
**2a**	3.564	35108207	58.82	80

Figure S10. GC spectra of **2b** (0.1 mmol), and **2a** from **1r** (0.1 mmol)

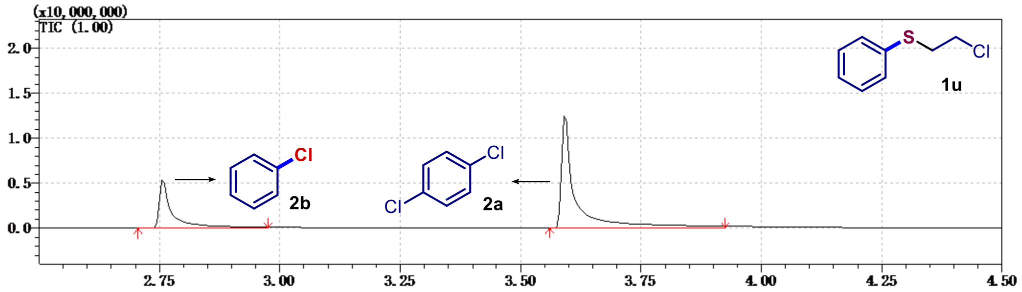

	Peak time	Peak area	Peak area (%)	Yield (%)
**2b**	2.757	10398931	29.47	75
**2a**	3.592	24887567	70.53	100

Figure S11. GC spectra of **2a** (0.1 mmol), and **2b** from **1u** (0.1 mmol)

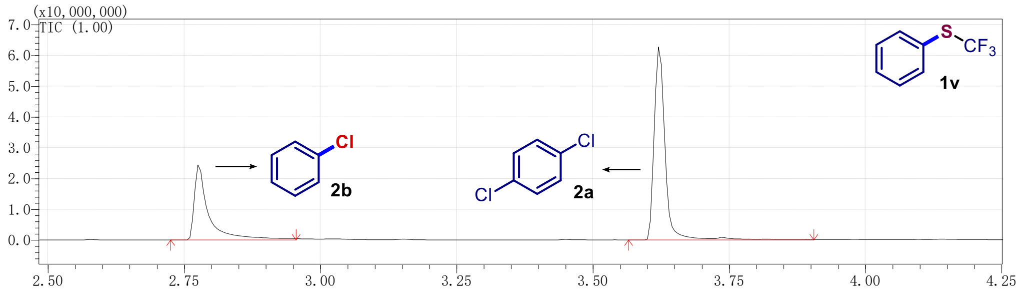

	Peak time	Peak area	Peak area (%)	Yield (%)
**2b**	2.776	42315040	33.18	89
**2a**	3.620	85216726	66.82	100

Figure S12. GC spectra of **2a** (0.1 mmol), and **2b** from **1v** (0.1 mmol)

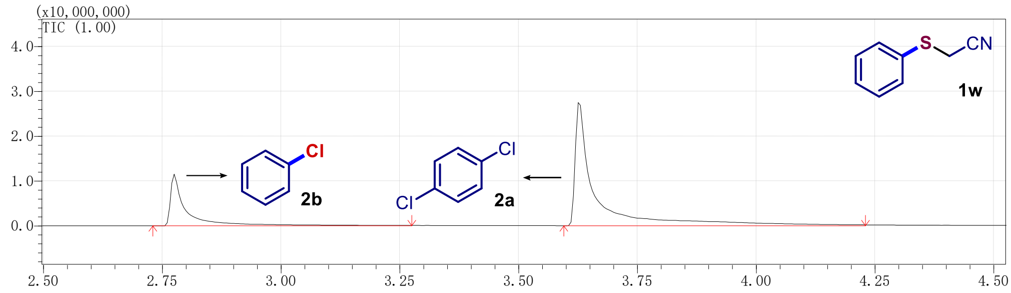

	Peak time	Peak area	Peak area (%)	Yield (%)
**2b**	2.775	27867709	25.18	60
**2a**	3.627	82798526	74.82	100

Figure S13. GC spectra of **2a** (0.1 mmol), and **2b** from **1w** (0.1 mmol)

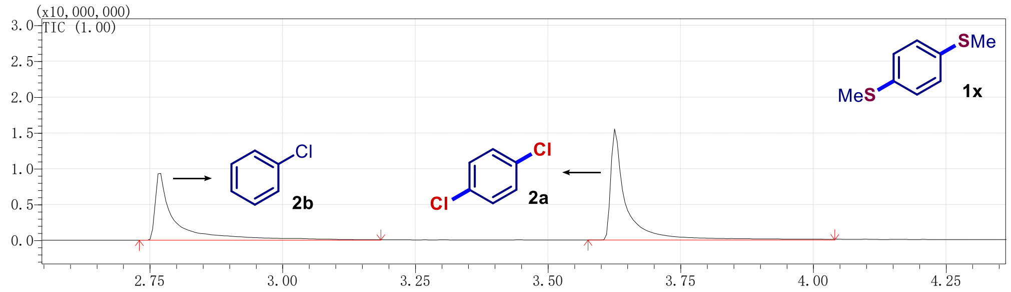

	Peak time	Peak area	Peak area (%)	Yield (%)
**2b**	2.768	25242551	47.05	100
**2a**	3.626	28407929	52.95	62

Figure S14. GC spectra of **2b** (0.1 mmol), and **2a** from **1x** (0.1 mmol)

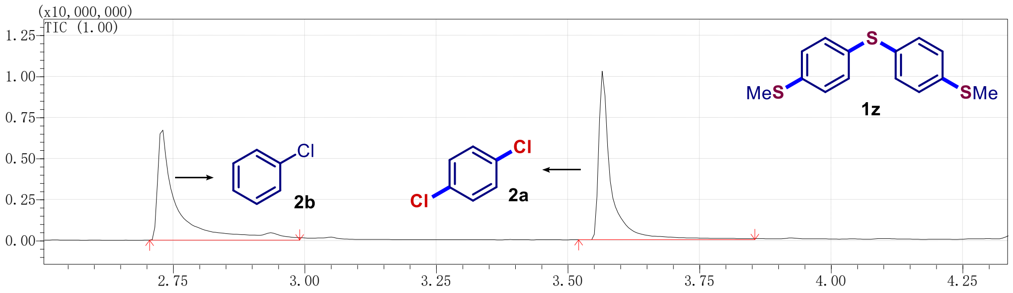

	Peak time	Peak area	Peak area (%)	Yield (%)
**2b**	2.725	17512951	52.88	100
**2a**	3.556	15605338	47.12	50

Figure S15. GC spectra of **2b** (0.2 mmol) and **2a** from **1z** (0.1 mmol) after 24 h

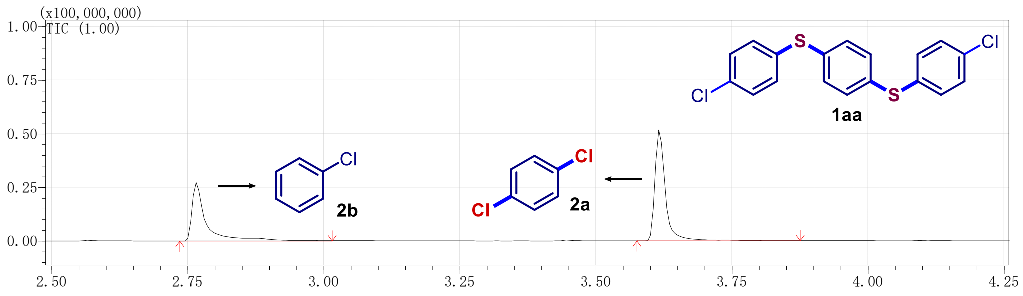

	Peak time	Peak area	Peak area (%)	Yield (%)
2b	2.766	50271211	41.47	100
**2a**	3.616	70947607	58.53	79

Figure S16. GC spectra of **2b** (0.3 mmol) and **2a** from **1aa** (0.1 mmol) after 24 h

^{19}F NMR data for volatile fluorine-containing aryl chlorides

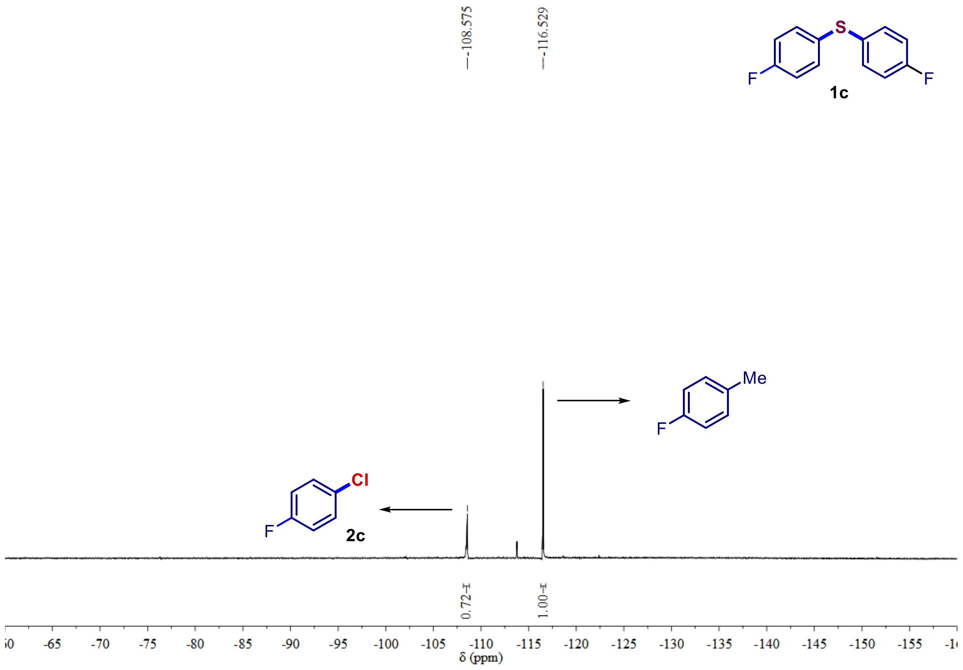

Figure S17. *p*-methylfluorobenzene (0.2 mmol), and **2c** from **1c** (0.1 mmol) as analyzed by ^{19}F NMR

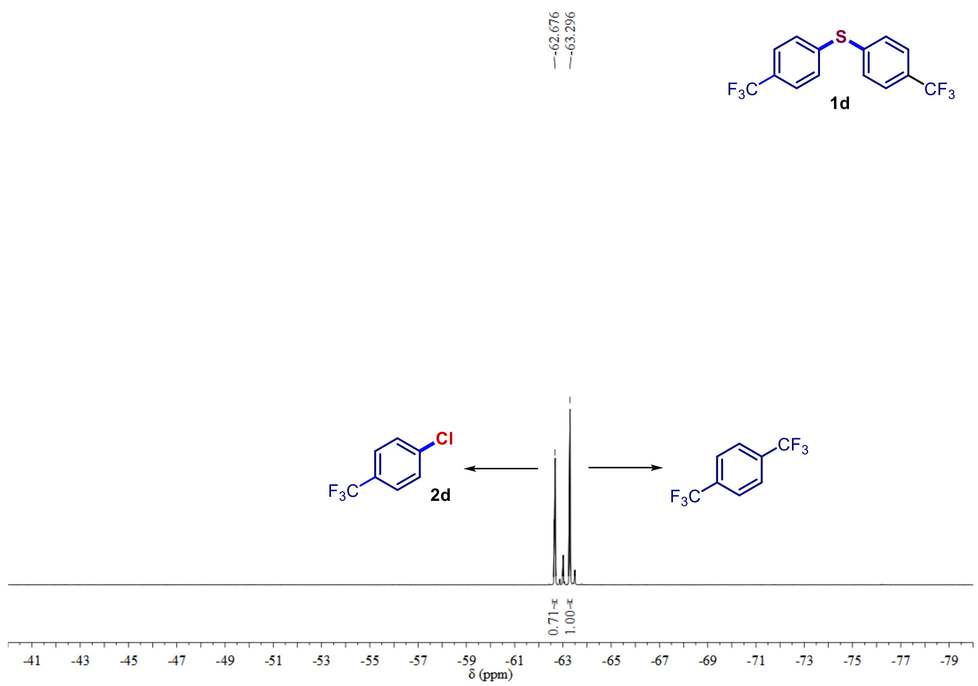

Figure S18. 1,4-bis(trifluoromethyl)benzene (0.1 mmol) and **2d** from **1d** (0.1 mmol) by ^{19}F NMR

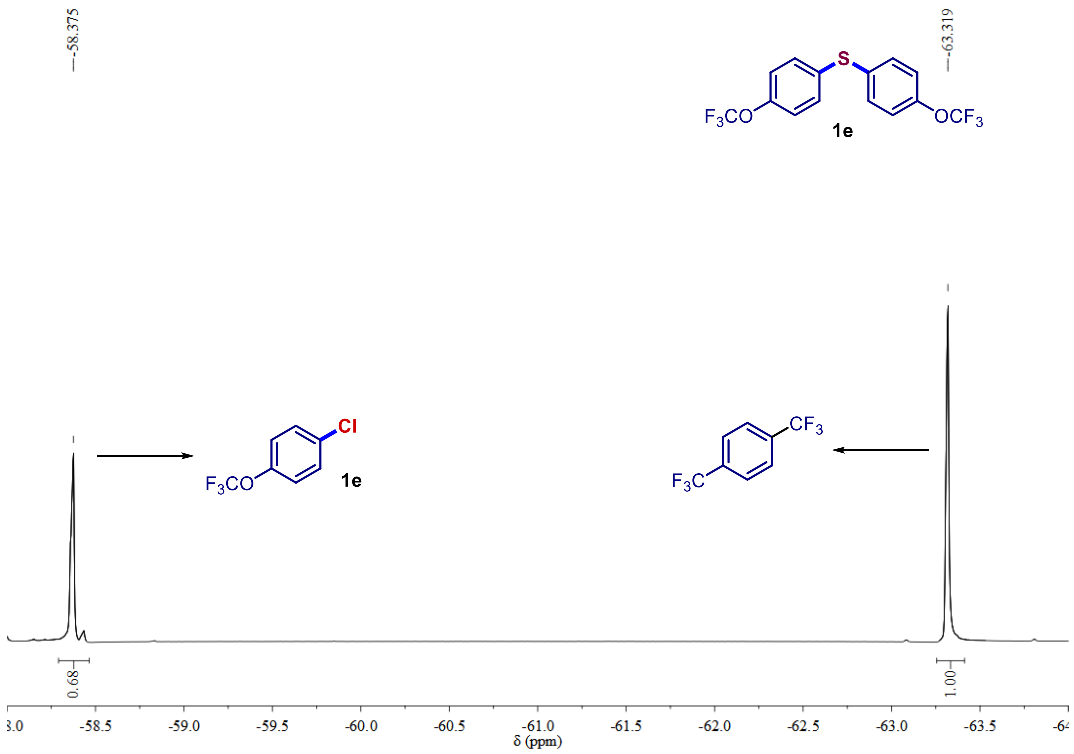

Figure S19. 1,4-bis(trifluoromethyl)benzene (0.1 mmol) and **2e** from **1e** (0.1 mmol) by ^{19}F NMR

The chemical upcycling of PPS resins and plastics

PPS 1 (CAS: 26125-40-6) was purchased from Macklin reagent, **PPS 7** and **8** were provided from Polyplastics Co. Ltd., and others were purchased from Taobao (https://www.taobao.com/). All PPS materials were used without further purification. Note: PPS resins or materials (**PPS 2-4** and **PPS 7-17**) were pulverized into fine powders (~50-100 mesh) using a ball mill at 30 Hz for 30 min prior to use. The purity of the PPS samples (**PPS1**, **PPS 9–17**) was determined through elemental analysis (**Figure S20**).

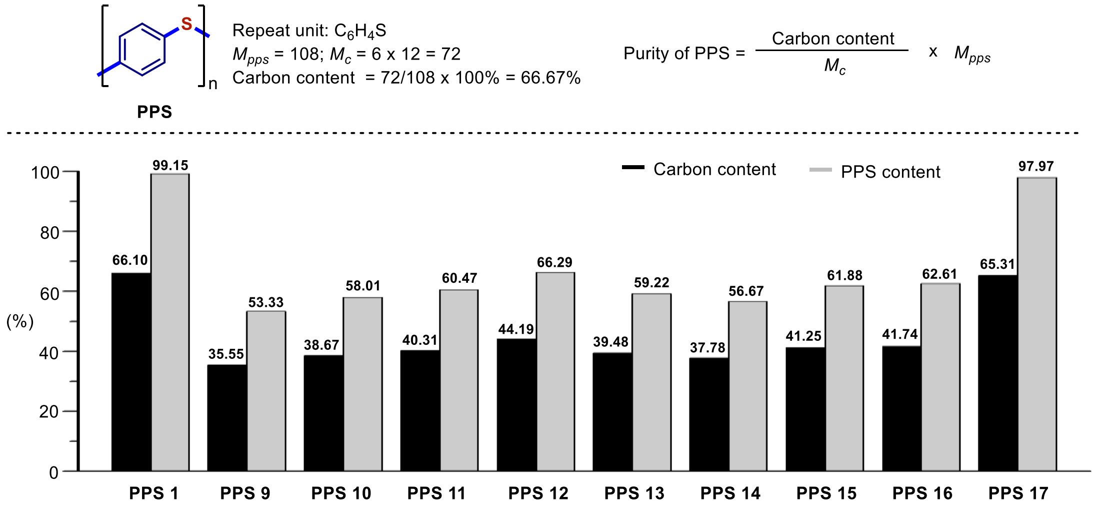

Figure S20. Purity of PPS resin and materials

(i) Optimization of the conditions for chemical upcycling of PPS

A 4 mL screw-capped vial with a stirring bar were added **PPS 1** (0.1 mmol of the repeated unit, 10.8 mg), catalyst (2-10 mol%) solvent and a chlorinated alkane (5-20 equiv). The vial was then sealed with a cap containing a PTFE septum and linked to an oxygen balloon (spherical diameter: ~25 cm). The reaction mixture was stirred under the irradiation of blue LEDs (400-405 nm, 12 W) at 25-30 °C for 24-48 h. The resulting solution was concentrated in vacuum and the residue was purified by flash chromatography on silica gel column to provide the product **2a**. GC-MS yields of **2a** were determined by adding **2b** (0.1 mmol) as an internal standard to the reaction mixture. Then, 5 μL of the solution was drawn, diluted immediately with ethyl acetate, and measured by GC-MS analysis. The selected results were summarized in **Table S2**.

Table S2. The optimization of the conditions for the reaction of **PPS 1**

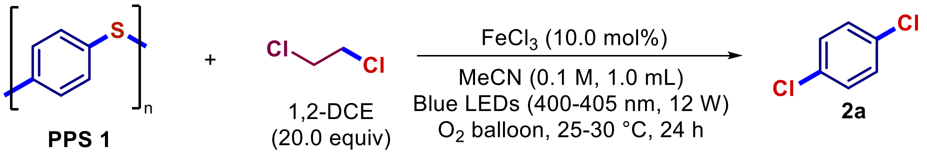

Entry	Variation from the standard conditions	Yield of **2a** (%)
**1**	**None**	**82(76)**
2	No $FeCl_3$	nd.
3	Under dark	nd.
4	N_2 atmosphere	nd.
5	No DCE	trace
6	$Fe(OTf)_3$ instead of $FeCl_3$	57
7	$Fe(NO_3)_3{\cdot}9H_2O$ instead of $FeCl_3$	58
8	10 equiv 1,2-DCE	55
9	5 equiv 1,2-DCE	39
10	DCM instead of 1,2-DCE	76
11	$CHCl_3$ instead of 1,2-DCE	40
12	CCl_4 instead of 1,2-DCE	61
13	1,1,2,2-tetrachloroethane instead of DCE	57
14	2 mol% $FeCl_3$	23
15	5 mol% $FeCl_3$	54
16	6 W instead of 12 W	55
17	1,2-DCE:MeCN (4:1,1 mL)	nd.
18	MeCN:1,2-DCE (4:1,1 mL)	68
19	1,2-DCE as the solvent	nd.
20	36 h instead of 24 h	84
21	48 h instead of 24 h	84
22	NaCl instead of DCE	7

GC yields and isolated yields in parentheses of **2a** were calculated on on a molar basis

(ii) General procedure for chemical upcycling of PPSs resins and plastic (GP3)

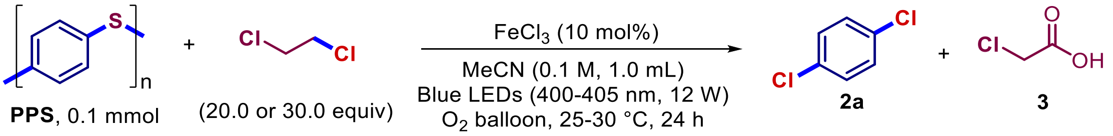

A 4 mL screw-capped vial with a stirring bar were added **PPS** materials (0.1 mmol of the repeated PPS unit, 10.8 mg of **PPS 1**, **5** and **6**; 10.8 mg/purity of **PPS 2-4** and **7-17**), $FeCl_3$ (10 mol%, 0.1 M in CH_3CN solution, 1.0 mL) and 1,2-DCE (20 equiv, 2.0 mmol, 160 μL) *[Note: For the reactions of* ***PPS 5-17****, 3.0 mmol 1,2-DCE were added]*. The vial was then sealed with a cap containing a PTFE septum and linked to an

oxygen balloon (spherical diameter: ~25 cm). The reaction mixture was stirred under the irradiation of blue LEDs (400-405 nm, 12 W) at 25-30 °C for 24 h. The resulting solution was concentrated in vacuum and the residue was purified by flash chromatography on silica gel column to provide the product **2a**. GC-MS yields of **2a** were determined by adding **2b** (0.1 mmol) as an internal standard to the reaction mixture. Then, 5 μL of the solution was drawn, diluted immediately with ethyl acetate, and measured by GC-MS analysis. See **Figure S21-S37** for details. The yields of **3** were obtained using dibromomethane (2.0 or 3.0 mmol) as an internal standard by ^{1}H NMR analysis (For example, **Figure S38** and **S39** for chemical upcycling of **PPS 1** and **PPS 5**, respectively).

GC-MS data

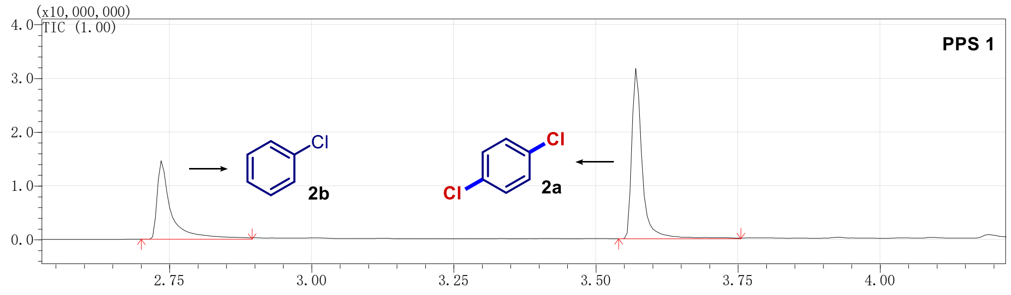

	Peak time	Peak area	Peak area (%)	Yield (%)
**2b**	2.736	25515975	40.53	100
**2a**	3.571	37439799	59.47	82

Figure S21. GC spectra for the chemical recycling of **PPS 1**

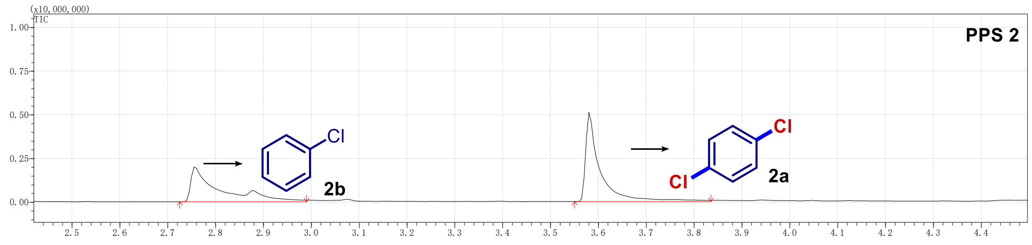

	Peak time	Peak area	Peak area (%)	Yield (%)
**2b**	2.757	8739193	44.79	100
**2a**	3.581	10772289	55.21	69

Figure S22. GC spectra for the chemical recycling of **PPS 2**

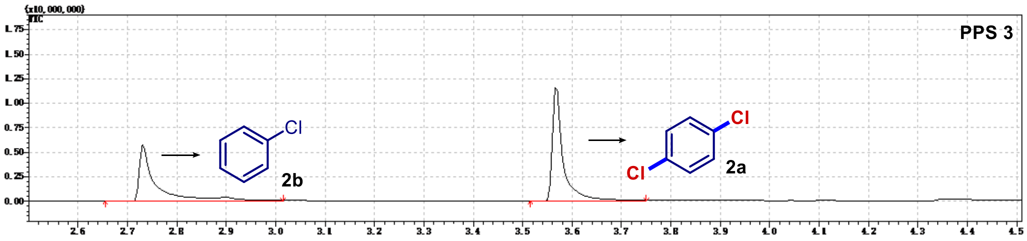

	Peak time	Peak area	Peak area (%)	Yield (%)
**2b**	2.731	13723767	43.17	100
**2a**	3.567	18066288	56.83	74

Figure S23. GC spectra for the chemical recycling of **PPS 3**

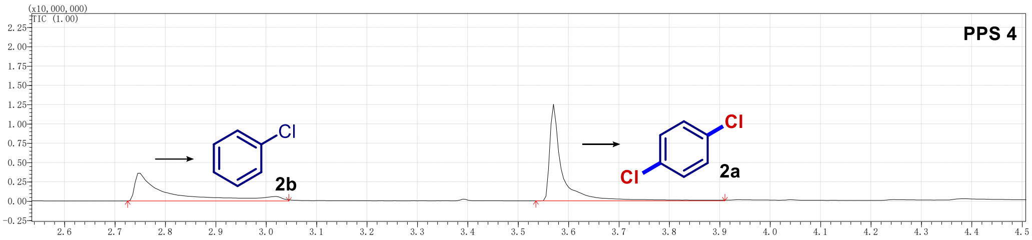

	Peak time	Peak area	Peak area (%)	Yield (%)
**2b**	2.748	15497749	40.39	100
**2a**	3.570	22872513	59.61	82

Figure S24. GC spectra for the chemical recycling of **PPS 4**

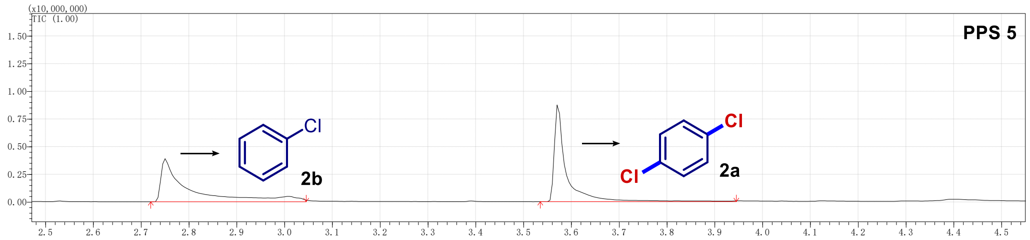

	Peak time	Peak area	Peak area (%)	Yield (%)
**2b**	2.750	13190571	45.01	100
**2a**	3.571	16113326	54.99	68

Figure S25. GC spectra for the chemical recycling of **PPS 5**

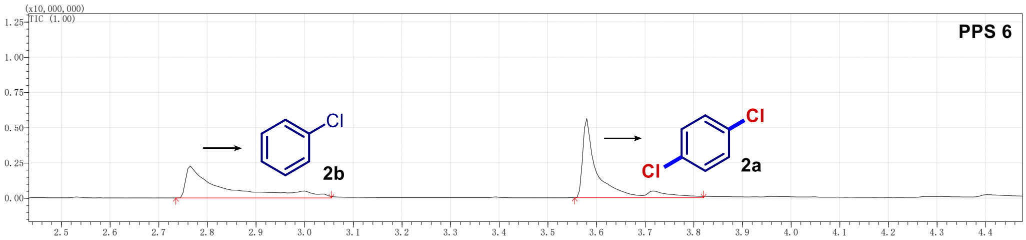

	Peak time	Peak area	Peak area (%)	Yield (%)
**2b**	2.765	10175084	48.31	100
**2a**	3.579	10887229	51.69	60

Figure S26. GC spectra for the chemical recycling of **PPS 6**

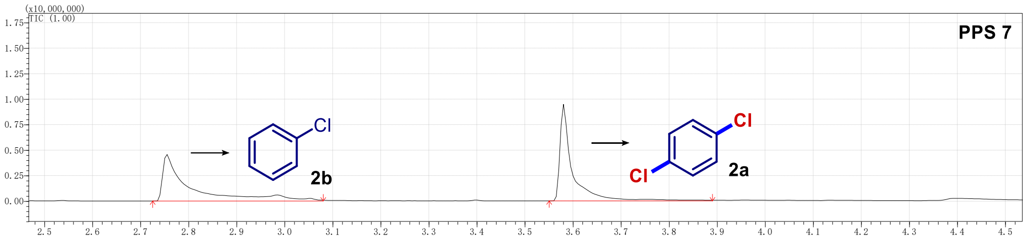

	Peak time	Peak area	Peak area (%)	Yield (%)
**2b**	2.754	14117143	45.42	100
**2a**	3.580	16961021	54.58	67

Figure S27. GC spectra for the chemical recycling of **PPS 7**

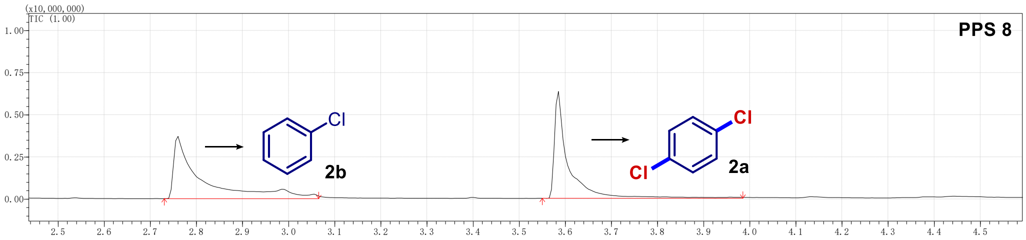

	Peak time	Peak area	Peak area (%)	Yield (%)
**2b**	2.759	15036699	51.52	100
**2a**	3.584	14149440	48.48	53

Figure S28. GC spectra for the chemical recycling of **PPS 8**

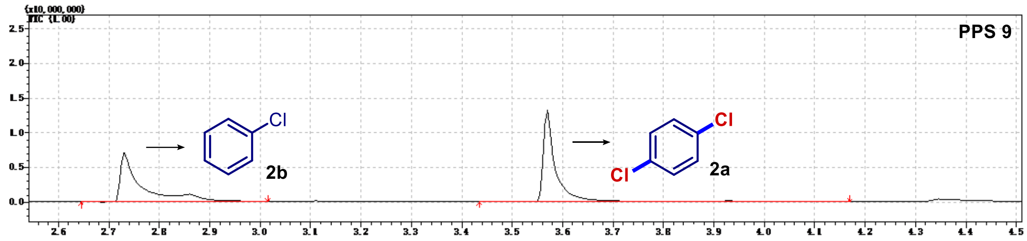

	Peak time	Peak area	Peak area (%)	Yield (%)
**2b**	2.731	22168500	48.18	100
**2a**	3.569	23843331	51.82	60

Figure S29. GC spectra for the chemical recycling of **PPS 9**

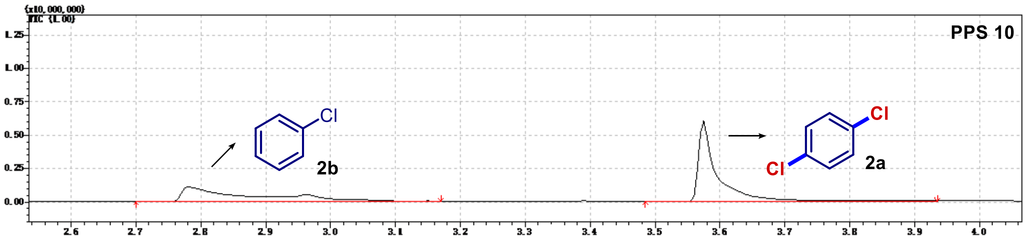

	Peak time	Peak area	Peak area (%)	Yield (%)
**2b**	2.781	8072890	39.03	100
**2a**	3.575	12611787	60.97	87

Figure S30. GC spectra for the chemical recycling of **PPS 10**

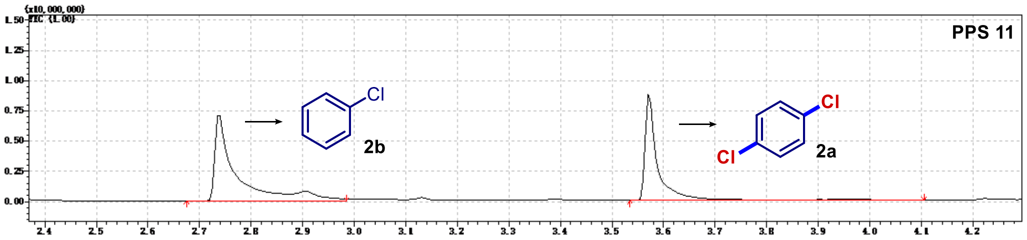

	Peak time	Peak area	Peak area (%)	Yield (%)
**2b**	2.737	19968551	55.47	100
**2a**	3.571	16030279	44.53	45

Figure S31. GC spectra for the chemical recycling of **PPS 11**

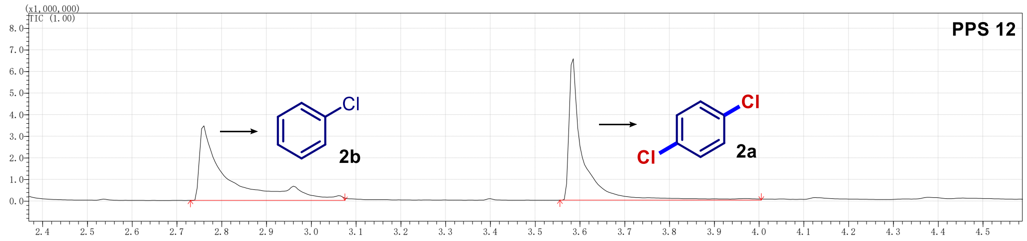

	Peak time	Peak area	Peak area (%)	Yield (%)
**2b**	2.759	14437442	50.75	100
**2a**	3.583	14010720	49.25	54

Figure S32. GC spectra for the chemical recycling of **PPS 12**

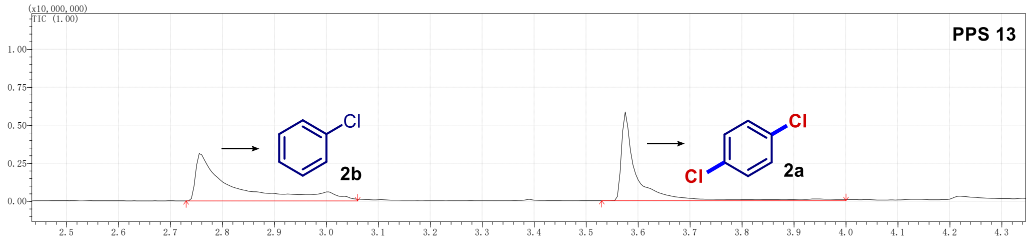

	Peak time	Peak area	Peak area (%)	Yield (%)
**2b**	2.757	12819616	51.35	100
**2a**	3.575	12145556	48.65	53

Figure S33. GC spectra for the chemical recycling of **PPS 13**

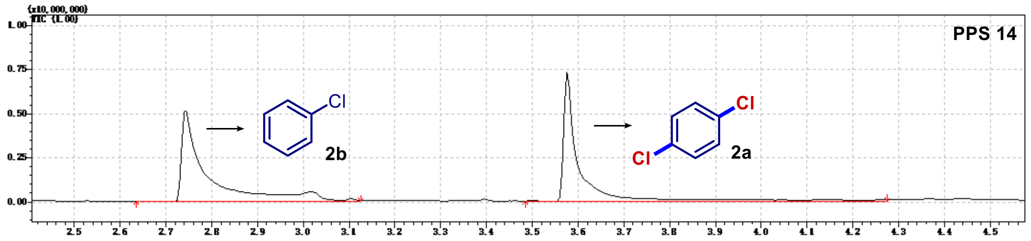

	Peak time	Peak area	Peak area (%)	Yield (%)
**2b**	2.742	20192875	55.05	100
**2a**	3.576	16488097	44.95	46

Figure S34. GC spectra for the chemical recycling of **PPS 14**

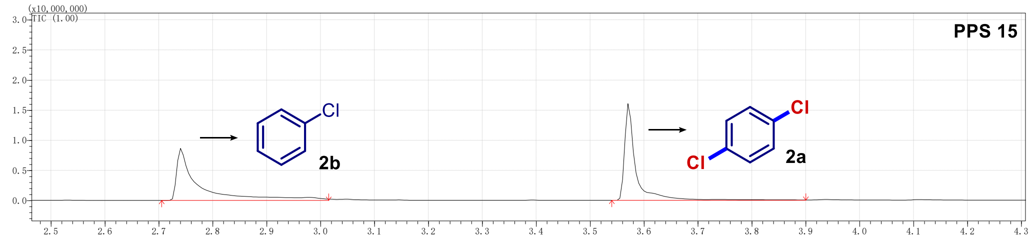

	Peak time	Peak area	Peak area (%)	Yield (%)
**2b**	2.741	21810963	48.11	100
**2a**	3.571	23524649	51.89	60

Figure S35. GC spectra for the chemical recycling of **PPS 15**

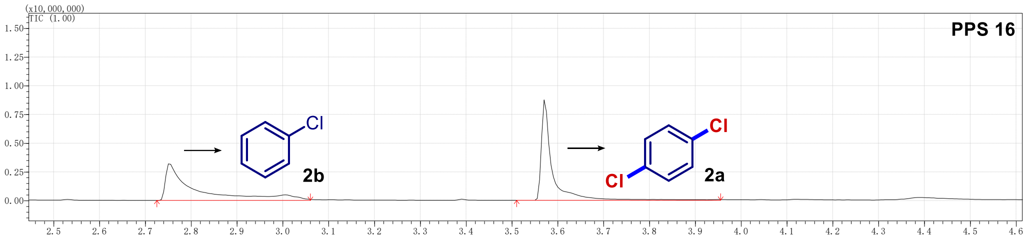

	Peak time	Peak area	Peak area (%)	Yield (%)
**2b**	2.752	12625212	46.54	100
**2a**	3.571	14502446	53.46	64

Figure S36. GC spectra for the chemical recycling of **PPS 16**

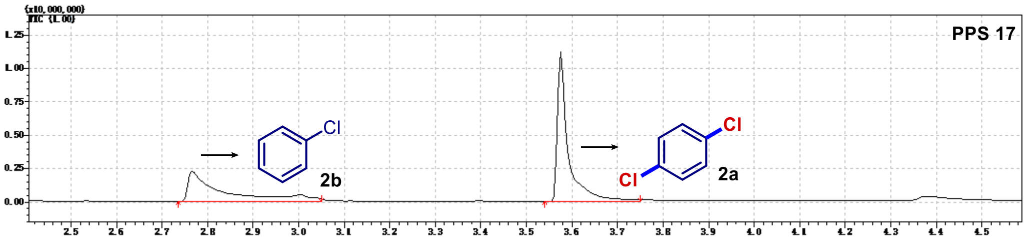

	Peak time	Peak area	Peak area (%)	Yield (%)
**2b**	2.766	11566185	38.45	100
**2a**	3.575	18514920	61.55	89

Figure S37. GC spectra for the chemical recycling of **PPS 17**

Examples of NMR data for the yields of 3

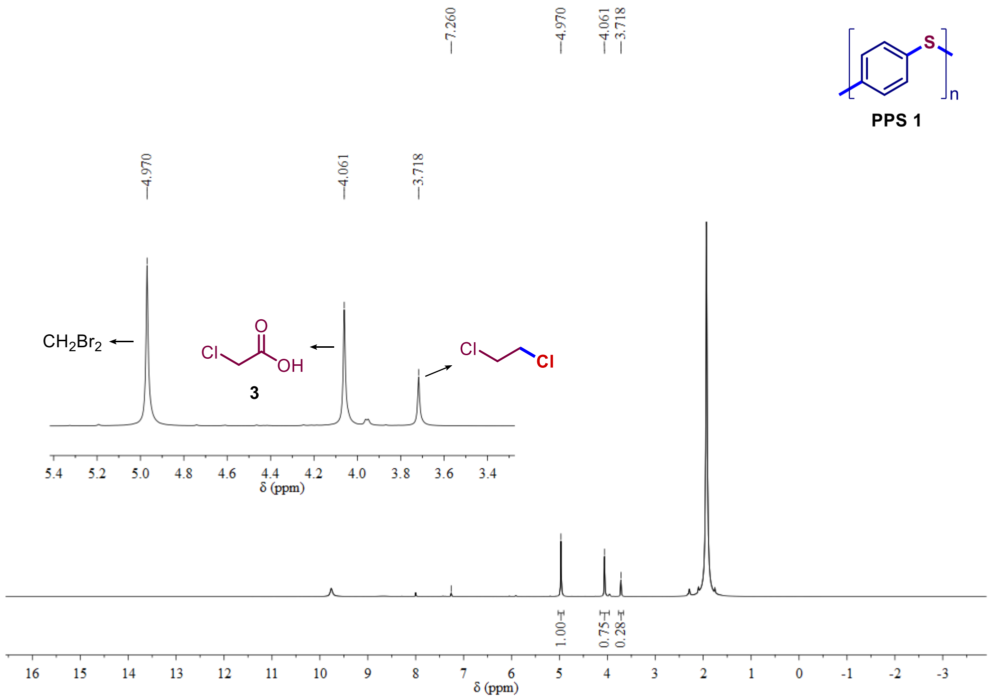

Figure S38. ^{1}H NMR spectra of **3**, 1,2-DCE and CH_2Br_2 (2.0 mmol) for the chemical recycling of **PPS 1**

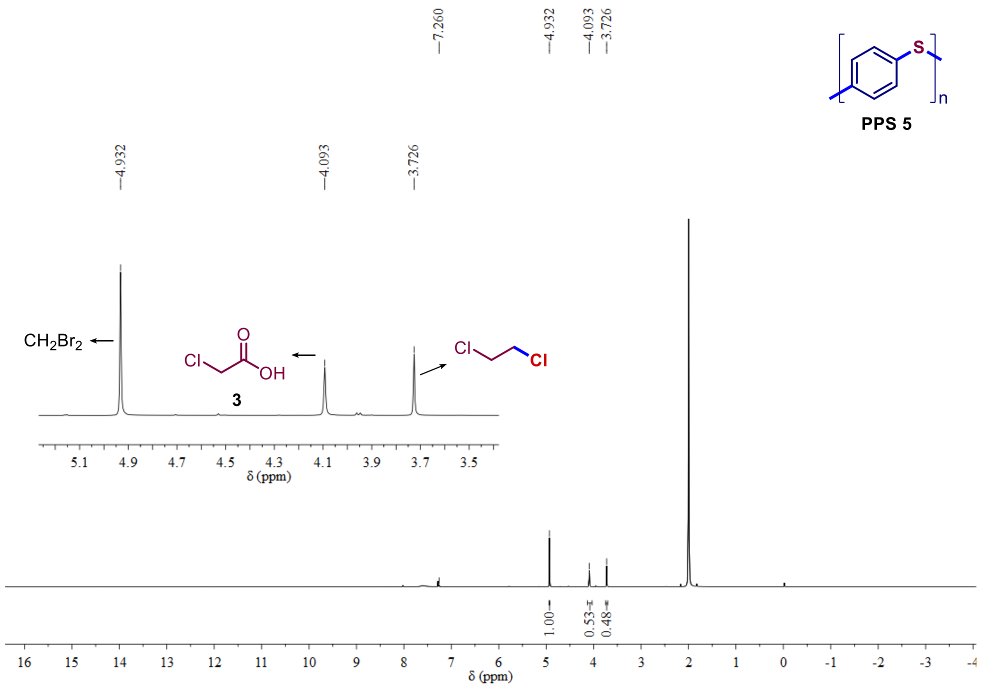

Figure S39. ^{1}H NMR spectra of **3,** 1,2-DCE and CH_2Br_2 (3.0 mmol) for the chemical recycling of **PPS 5**

(iii) The chemical upcycling of PASK

The synthesis of PASK[9]

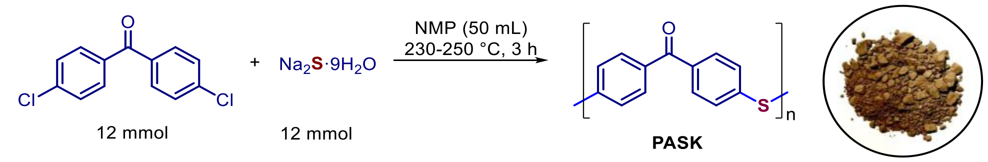

A 100 mL three-neck flask containing a stirring bar equipped with a reflux condenser was charged with bis(4-chlorophenyl)methanone (12 mmol, 3.0 g), $Na_2S·9H_2O$ (12 mmol, 3.4 g), NMP (50 ml) and deionized water (5.0 ml) under N_2 atmosphere. The reaction mixture was heated at 230-250 °C for 3 h. After cooling to room temperature, the resulting mixture was poured into acetone (100 mL) to precipitate brown solids. The solids were collected, washed with hot acetone (10.0 mL) and water (60 °C, 10.0 mL) for three times to remove any inorganic salts, unreacted materials and oligomers, and dried in vacuo, affording 848.1 mg of polyarylene sulfide ketone (PASK) (40% yield).

The chemical upcycling of PASK via the iron-photocatalysis.

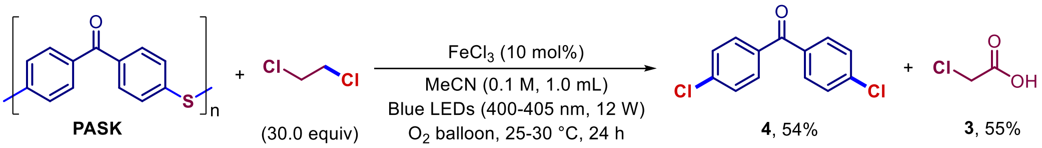

A 4 mL screw-capped vial with a stirring bar were added **PASK** (21.2 mg, 0.1 mmol of the repeated unit), $FeCl_3$ (10 mol%, 0.1 M in CH_3CN solution, 1.0 mL), and 1,2-DCE (3 mmol, 30 equiv, 0.24 mL). The vial was then sealed with a cap containing a PTFE septum and linked to an oxygen balloon (spherical diameter: ~25 cm). The reaction mixture was stirred under the irradiation of blue LEDs (400-405 nm, 12 W) at 25-30 °C for 24 h. The resulting solution was concentrated in vacuum and the residue was purified by flash chromatography on silica gel column to provide the product **4**. The yields of **3** (55%) were obtained by adding dibromomethane (3.0 mmol) as an internal standard to the reaction mixture. Then, 100 μL was taken and diluted with $CDCl_3$ and analyzed by ^1H NMR.

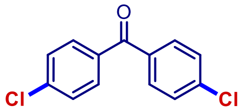

Bis(4-chlorophenyl)methanone (4): The title compound was isolated as a white solid (13.4 mg, 54%) after chromatography on silica gel (PE/EA = 20:1). ^{1}H NMR (400 MHz, $CDCl_3$): δ 7.73 (d, J = 8.4 Hz, 4H), 7.47 (d, J = 8.4 Hz, 4H). ^{13}C NMR (100 MHz, $CDCl_3$): δ 194.4, 139.3, 135.7, 131.5, 128.9. GC-MS (EI$^+$): calcd for $C_{13}H_8Cl_2O$ [M]$^+$ 250.00, found 249.95.

(iv) The gram-scaled chemical upcycling of PPS

To a 250 mL oven-dried three-neck flask equipped with a magnetic stir bar was added with pretreated **PPS 4** powder (2.32 g, 15 mmol PPS), $FeCl_3$ (10 mol%, 1.5 mmol, 243.3 mg), CH_3CN (100 mL) and 1,2-DCE (0.3 mol, 20 equiv, 24 mL). The reaction was stirred and irradiated with four LEDs lights (400 nm, 50 W, each has a cooling fan to keep the reaction temperature at 25-30 °C) for 5 days. O_2 gas was bubbled into the solution at a rate of 3 mL/min. Upon completion of the reaction, the reaction mixture was filtrated and the residues were collected and dried in vacuo to give 0.91 g of light-yellow solid. The filtrate solution was then concentrated in vacuum to give 88 mL of mixed-solvents (~78% recovery) the give a thick residual. After adding 100 mL of petroleum ether and 100 mL of H_2O, the mixture was extracted and the combined organic phase was concentrated in vacuum. The combined organic layer was purified by column chromatography on silica gel using PE/EA = 50:1 as the eluent to yield the corresponding product **2a** as a white solid (1.32 g, 60%). NaOH solution (3M, 50 mL) was gradually added to the aqueous phase and a brown precipitate was precipitated, which was then collected, washed with H_2O and dried to obtain $Fe(OH)_3$ (144.1 mg, 90% iron recovery). The aqueous layer was then acidified by hydrochloric acid (3 M, 55 mL), and concentrated to yield crude solids of chloroacetic acid and sodium chloride. The chloroacetic acid was dissolved in dichloromethane (3 x 50 mL), separated, and concentrated in vacuo to provie a light-yellow solid (13.6 g, 48%).

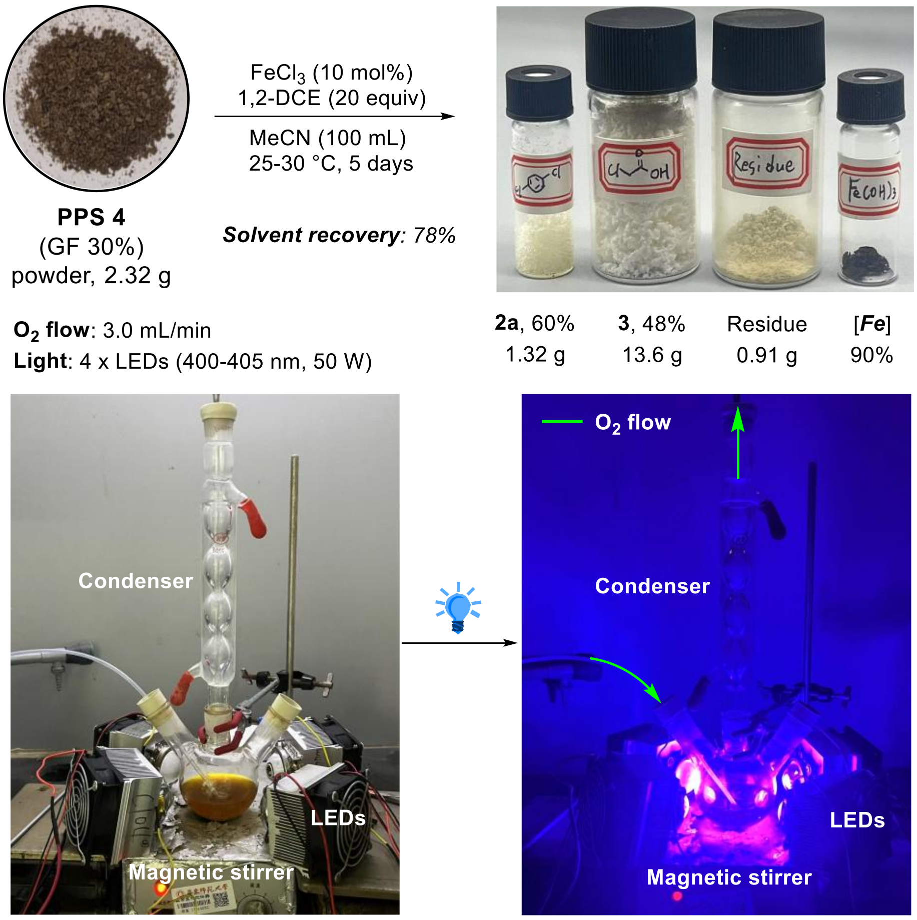

Figure S40. Chemical upcycling of **PPS 4** in gram scale

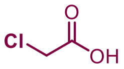

2-Chloroacetic acid (3): ^{1}H NMR (400 MHz, $CDCl_3$): δ 11.35 (br, s, 1H), 4.15 (s, 2H). ^{13}C NMR (100 MHz, $CDCl_3$): δ 173.7, 40.6. GC-MS (EI^+): calcd for $C_2H_3ClO_2$ $[M]^+$ 93.98, found 94.00.

(v) Mixed-plastic experiments

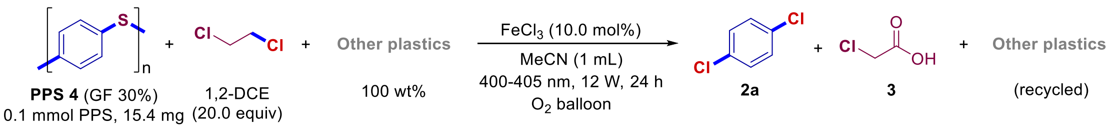

PPS 4 (15.4 mg, 0.1 mmol PPS), $FeCl_3$ (10 mol%, 0.1 M in CH_3CN solution, 1.0 mL), 1,2-DCE (2 mmol, 20 equiv, 160 μL), and ~100 wt% of other general plastic (LDPE, HDPE, PP, PET or PA-66) was added to a 4 mL screw-capped vial. The reaction mixture was stirred under the irradiation of blue LEDs (400-405 nm, 12 W) at 25-30 °C for 24 h. After reaction, the unreacted/undissolved other plastics were

collected, washed several times with EA, and dried in vacuo (**Figure S41**). Yields of **2a** and **3** were obtained according to GP3, respectively. The results were summarized in **Table S3**.

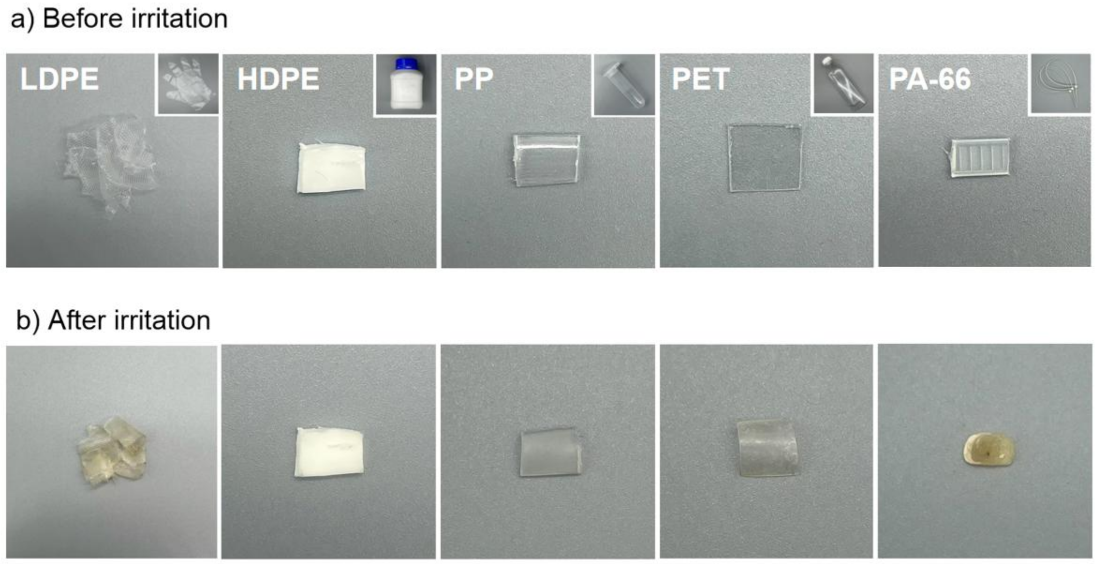

Figure S41. Chemical upcycling of PPS with other plastics before and after irradiation

Table S3. Mixed-plastic experiments

Other plastics	LDPE	HDPE	PP	PET	PA
Initial weight (mg)	16.2	15.8	16.6	15.5	16.0
Final weight (mg)	16.1	15.5	16.4	15.0	14.4
Weight recovery (%)	99	98	99	97	90
Yield of **2a** (%)	82	69	62	75	75
Yield of **3** (%)	74	75	74	72	75

Mechanistic studies on the chemical upcycling of PPS

(i) Reaction tracking by elemental analysis

PPS 9 (20.3 mg, 0.1 mmol of PPS unit), $FeCl_3$ (10 mol%, 0.1 M in CH_3CN solution, 1.0 mL), 1,2-DCE (3.0 mmol, 240 μL) were added to a 4 mL screw-capped vial. The vial was then sealed with a cap containing a PTFE septum and linked to an oxygen balloon (spherical diameter: ~25 cm). The reaction mixture was stirred under the irradiation of blue LEDs (400-405 nm, 12 W) at 25-30 °C for the indicated time (6 h,

12 h, 18 h, 24 h and 30 h). The residues were collected and washed with water and ethyl acetate for three times, and then dried in vacuo. Element analysis of the residues indicated a decrease trend of the carbon content, confirming a diminishing proportion of PPS oligomers during the desulfurizing chlorination process (**Table S4** and **Figure S42**).

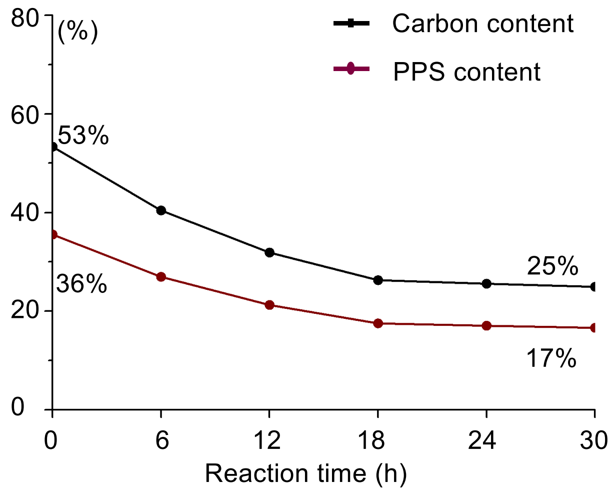

Figure S42. Element analysis of **PPS 9** upcycling

Table S4. Reaction tracking by elemental analysis

Reaction time (h)	0	6	12	18	24	30
Carbon content (%)	53	40	32	26	26	25
PPS content (%)	36	27	21	18	17	17

(ii) High-temperature gel permeation chromatography

According to GP3, **PPS 1** (10.8 mg, 0.1 mmol PPS) was added. The reaction mixture was stirred under the irradiation of blue LEDs (400-405 nm, 12 W) at 25-30 °C for the indicated time (0 h, 2 h, 4 h and 12 h). The residues of six trials were collected and washed with water and ethyl acetate for three times, and then dried in vacuo. 10 mg of the residues was dissolved in 1-chloronaphthalene (4.0 mL) at 210 °C and subjected to high temperature GPC analysis (**Table S5**).[10] The HGPC profiles revealed a notable reduction of M_w from 27.1 kg/mol to 2.6 kg/mol within 12 hours, indicating extensive polymer chain scissions (**Figure S43**).

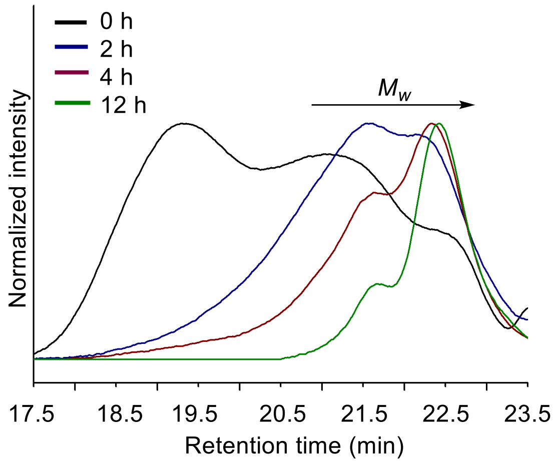

Figure S43. High-temperature GPC profiles of **PPS 1** upcycling

Table S5. High-temperature GPC data for the indicated time

Time (h)	M_p	M_n	M_w	M_z
0	40891	4509	27057	68629
2	4683	2936	9092	32119
4	2348	2612	6607	26131
12	2167	1865	2620	3620

(iii) Fourier transform infrared (FTIR) spectra

According to GP3, **PPS 1** (10.8 mg, 0.1 mmol PPS) was added. The reaction mixture was stirred under the irradiation of blue LEDs (400-405 nm, 12 W) at 25-30 °C for the indicated time (0 h, 6 h and 12 h). The residues of six trials were collected and washed with water and ethyl acetate for three times, and then dried in vacuo. FTIR data showed a newly emerged peak exhibited at 1049 cm^{-1}, associated to aryl C-Cl bond (**Figure S44**).

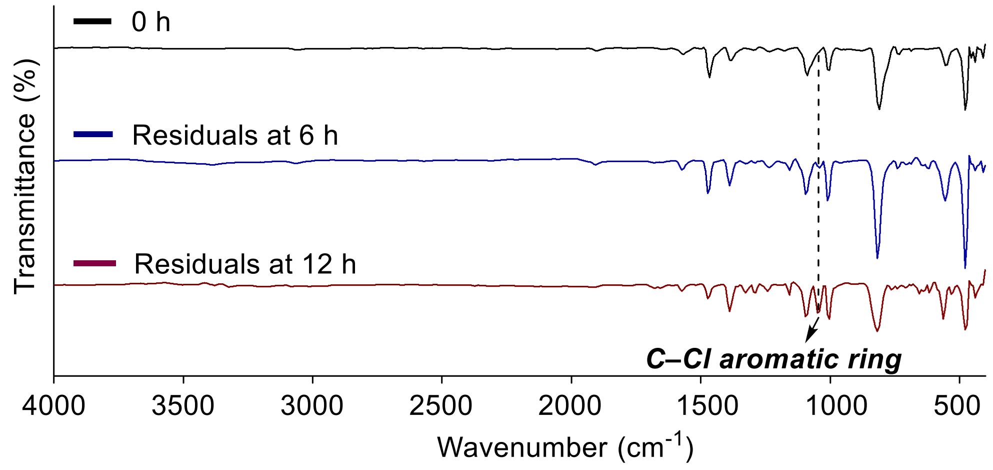

Figure S44. Fourier transform infrared (FTIR) spectra of the residues from **PPS 1**

(iv) MALDI-TOF-MS analysis

According to GP3, **PPS 1** (10.8 mg, 0.1 mmol PPS) was added. The reaction mixture was stirred under the irradiation of blue LEDs (400-405 nm, 12 W) at 25-30 °C for 24 h. The remaining solids of **PPS 1** (four trials) were collected and washed with water and ethyl acetate for three times, and then dried in vacuo. MALDI-TOF-MS analysis of the residues identified various peaks matching the expected chlorinated PPS oligomers with *m/z* values ranking from 240 to 800, and other unidentified peaks (**Figure S45**).

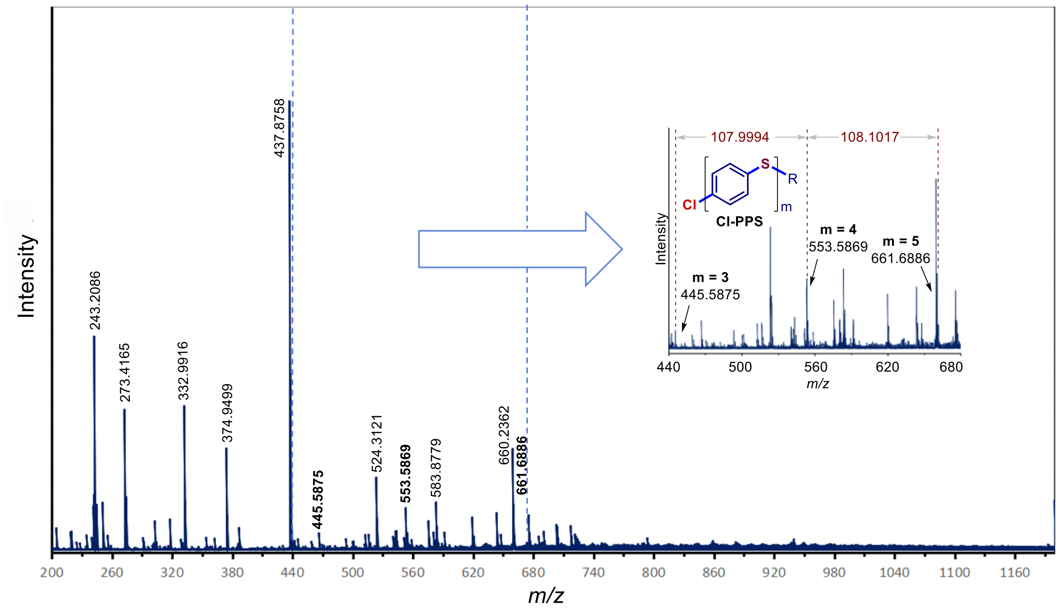

Figure S45. MALDI-TOF-MS data of the residues from **PPS 1**

(v) Differential scanning calorimetry (DSC) measurement

According to GP3, **PPS 1** (10.8 mg, 0.1 mmol PPS) was added. The reaction mixture was stirred under the irradiation of blue LEDs (400-405 nm, 12 W) at 25-30 °C for the indicated time (0 h, 6 h and 12 h). The residues of six trials were collected and washed with water and ethyl acetate for three times, and then dried in vacuo. The samples were firstly heated to 300 °C at the rate of 10 °C/min at N_2 atmosphere, then cooled to room temperature at the same rate, and subsequently heated again to 300 °C. The DSC curves indicated a sharp decline in both the melting endotherms (ΔH_m) and melting temperature (T_m) of **PPS 1** after light irradiation (**Figure S46**).

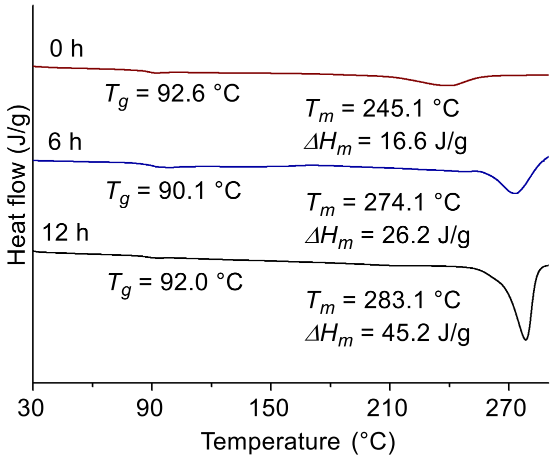

Figure S46. DSC curves of the residues from **PPS 1**

(vi) X-ray diffraction (XRD) measurement

According to GP3, **PPS 1** (10.8 mg, 0.1 mmol PPS) was added. The reaction mixture was stirred under the irradiation of blue LEDs (400-405 nm, 12 W) at 25-30 °C for the indicated time (0 h, 6 h and 12 h). The residues of six trials were collected and washed with water and ethyl acetate for three times, and then dried in vacuo. The samples were tested by XRD with a 2θ ranging from 2° to 50°, which observed pronounced attenuation of the characteristic crystalline peak (2θ = 20.88°), thus suggesting the destruction of long-range crystalline order within PPS chains scissions (**Figure S47**).

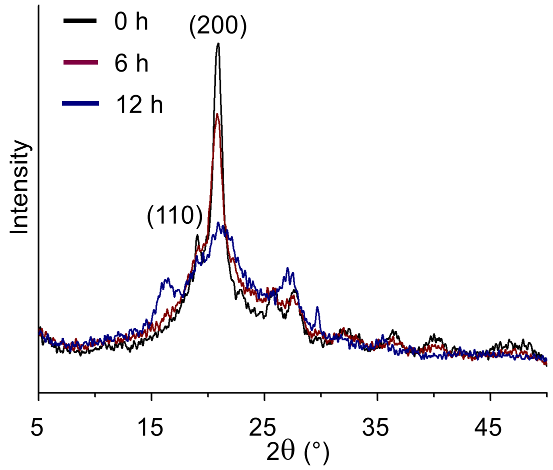

Figure S47. XRD spectra of the residues from **PPS 1**

(vii) Thermogravimetric analysis (TGA) measurement

According to GP3, **PPS 1** (10.8 mg, 0.1 mmol PPS) was added. The reaction mixture was stirred under the irradiation of blue LEDs (400-405 nm, 12 W) at 25-30 °C for the indicated time (0 h, 6 h and 12 h). The residues of six trials were collected and washed with water and ethyl acetate for three times, and then dried in vacuo. TGA curves indicated that PPS residues generated from chain scissions and polymer matrix disruption during PPS upcycling exhibited diminished thermal stability compared to original PPS polymers, which decomposed at reduced temperature within 50 wt% loss, and reached up to 61 wt% loss (**Figure S48**).

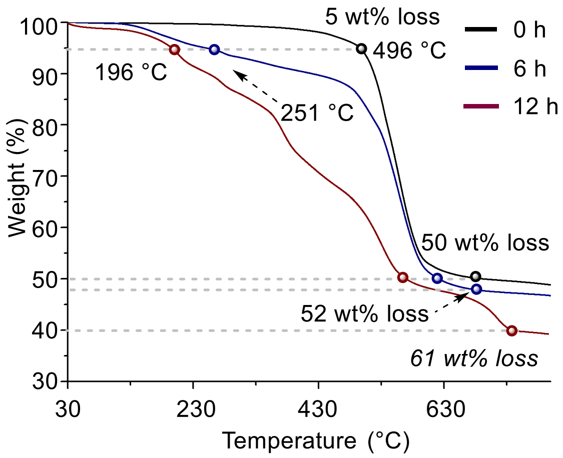

Figure S48. TGA curves of the residues from **PPS 1**

(viii) Water contact angle test

According to GP3, **PPS 1** (10.8 mg, 0.1 mmol PPS) was added. The reaction mixture was stirred under the irradiation of blue LEDs (400-405 nm, 12 W) at 25-30 °C for the indicated time (0 h, 6 h, 12 h and 24 h). The residues of six trials were collected and washed with water and ethyl acetate for three times, and then dried in vacuo. The water contact angle decreased from an average of 139.9° to 92.3° for the residual oligomers revealed the enhanced surface wettability (**Figure S49**).

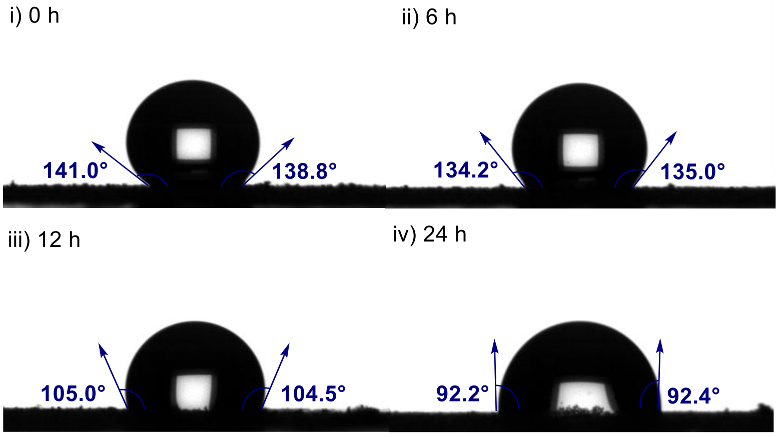

Figure S49. Water contact angle of the residues from **PPS 1**

(ix) Scanning electron microscopy (SEM)

According to GP3, **PPS 1** (10.8 mg, 0.1 mmol PPS) was added. The reaction mixture was stirred under the irradiation of blue LEDs (400-405 nm, 12 W) at 25-30 °C for the indicated time (0 h, 12 h and 24 h). The residues of six trials were collected and washed with water and ethyl acetate for three times, and then dried in vacuo. The collected solids and **PPS 1** were each taken 10 mg tested by SEM. The formation of micropores, fissures, and lamellar structures was founded, demonstrating the hierarchical structural disintegration by disrupting the crystalline domains of PPS plastics (**Figure S50**).

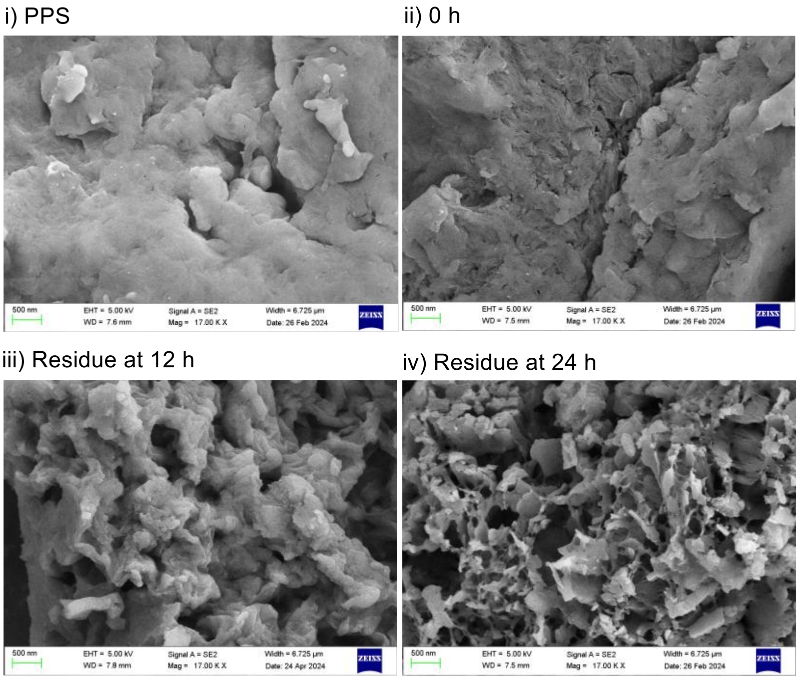

Figure S50. SEM images of the residues from **PPS 1**

Mechanistic studies on iron-photocatalyzed C–S bond cleavage

(i) Radical quenching experiments

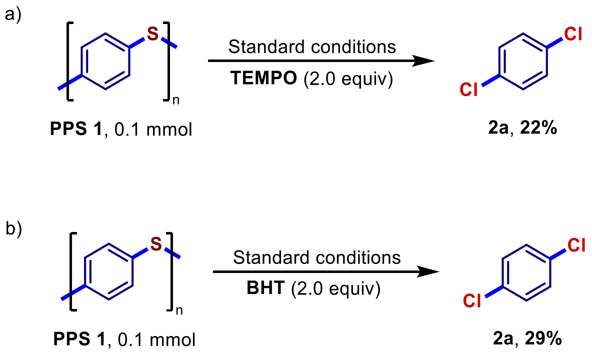

Figure S51. The reaction of **PPS 1** with TEMPO or BHT

PPS 1 (10.8 mg, 0.1 mmol PPS), $FeCl_3$ (10 mol%, 0.1 M in CH_3CN solution, 1.0 mL), 1,2-DCE (2 mmol, 20 equiv, 0.16 mL) and TEMPO (0.2 mmol, 31.3 mg) were added to a 4 mL screw-capped vial. The reaction mixture was stirred under the irradiation of blue LEDs (400-405 nm, 12 W) at 25-30 °C for 24 h. The yield of **2a** reduced to 22% as detected by GC-MS.

PPS 1 (10.8 mg, 0.1 mmol PPS), $FeCl_3$ (10 mol%, 0.1 M in CH_3CN solution, 1.0 mL), 1,2-DCE (2 mmol, 20 equiv, 0.16 mL) and BHT (0.2 mmol, 44.2 mg) were

added to a 4 mL screw-capped vial. The reaction mixture was stirred under the irradiation of blue LEDs (400-405 nm, 12 W) at 25-30 °C for 24 h. The yield of **2a** reduced to 29% as detected by GC-MS.

(ii) UV-Vis spectroscopy

The 1*10^{-4} M solution of $FeCl_3$ was prepared in mixed acetonitrile/1,2-DCE (100:16). The mixture of **PPS 1** in 1*10^{-3} M of mixed acetonitrile/1,2-DCE were irradiated for the indicated time (0, 12 and 24 h), and diluted into a concentration of 1*10^{-4} M. UV-Vis spectroscopy revealed two emission peaks (λ_{max}) at 312 nm and 363 nm, indicating that $FeCl_3$ severed as the photocatalyst (**Figure S52**).

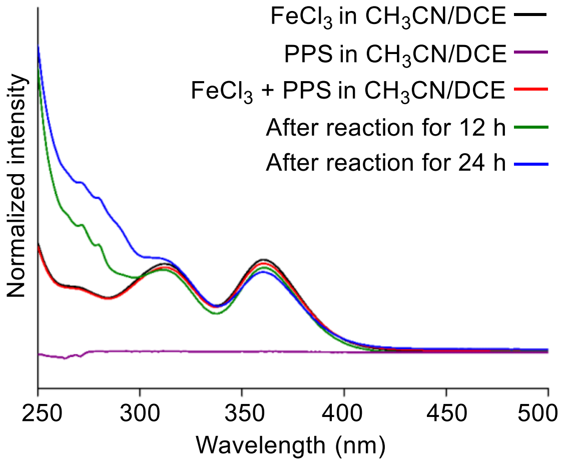

Figure S52. UV-Vis absorption spectra

(iii) Light-on/off experiment

According to GP3, **PPS 1** (10.8 mg, 0.1 mmol PPS) was added. The reaction mixture was stirred under the irradiation of blue LEDs (400-405 nm, 12 W) at 25-30 °C. The reaction mixture was stirred with or without irradiation under blue LEDs (400-405 nm, 12 W) at 25-30 °C. 5 µL of the solution was drawn at intervals for 1 hours, diluted immediately with ethyl acetate and measured by GC-MS analysis to determine the yields of **2a**. The results were summarized in **Figure S53**, excluding the radical chain mechanism of this reaction.

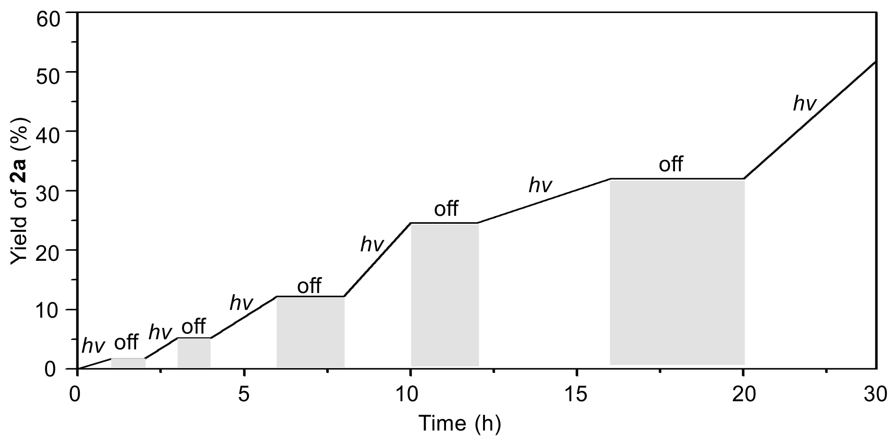

Figure S53. Reaction profile of light on/off experiments for the reaction of **PPS 1**

(iv) The detections of active radical species.

(a) Control experiments to probe the formation of the chlorine radical

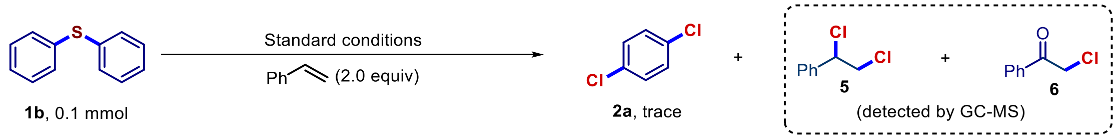

1b (0.1 mmol, 18.6 mg), $FeCl_3$ (2 mol%, 0.02 M in CH_3CN solution, 1.0 mL), 1,2-DCE (1 mmol, 10 equiv, 0.08 mL) and styrene (0.2 mmol) were added to a 4 mL screw-capped vial. The reaction mixture was stirred under the irradiation of blue LEDs (400-405 nm, 12 W) at 25-30 °C for 6 h. The yield of **2b** reduced to trace amount as detected by GC-MS. In the reaction, chlorinated adducts **5** and **6** were detected by GC-MS, supporting the formation of chlorine radicals (**Figure S54**).

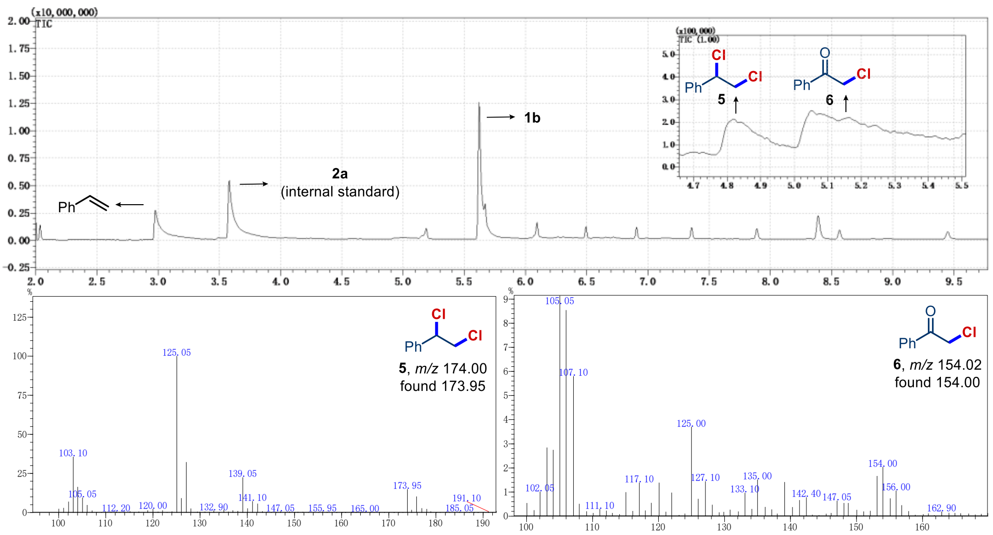

Figure S54. Detection of **5** and **6** for the reaction of **1b** with styrene

When the reaction of **PPS 1** was performed in the presence of 0.12 mmol of 5,5-dimethyl-1-pyrroline *N*-oxide (DMPO). Without light irradiation, no obvious signal was obtained as measured by EPR (**Figure S55**, top), and new peaks assigned to the chloride radical and hydroxide radical were observed after irradiation under blue LEDs (400-405 nm, 12 W) at 25-30 ºC for 10 mins (**Figure S55**, bottom).

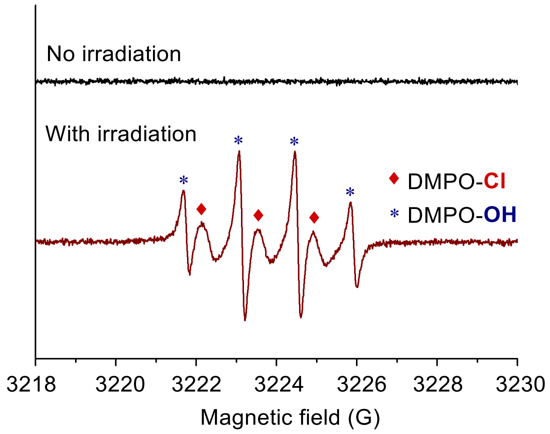

Figure S55. EPR spectra for detection of chloride radicals

(b) The detection of superoxide radicals

When the reaction of **PPS 1** was performed in the presence of 0.12 mmol of 5,5-dimethyl-1-pyrroline *N*-oxide (DMPO). Without light irradiation, no obvious signal was obtained as measured by EPR (**Figure S56**, top), and new peaks assigned to the superoxide radical were observed after irradiation under blue LEDs (400-405 nm, 12 W) at 25-30 ºC for 10 mins (**Figure S56**, bottom).

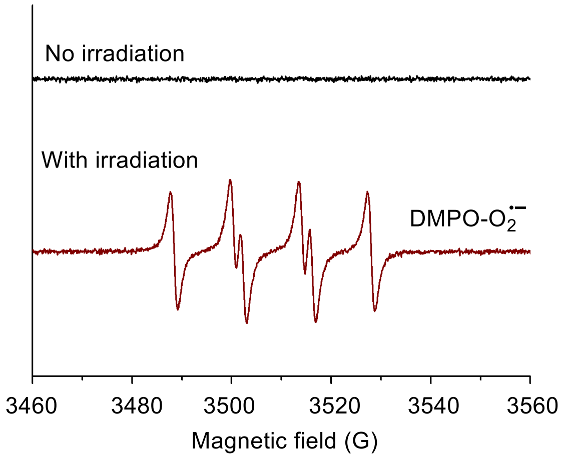

Figure S56. EPR spectra for detection of superoxide radicals

(c) Detection of the phenylthiyl radicals

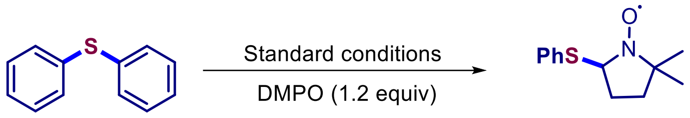

1b (18.6 mg, 0.1 mmol), $FeCl_3$ (2 mol%, 0.02 M in CH_3CN solution, 1.0 mL), 1,2-DCE (1 mmol, 10 equiv, 0.08 mL) and 0.12 mmol of 5,5-dimethyl-1-pyrroline *N*-oxide (DMPO) were added. The reaction mixture was taken and measured by EPR before irradiation and no obvious signal was obtained (**Figure S57**, top). Then the reaction mixture was stirred under the irradiation of blue LEDs (400-405 nm, 12 W) at 25-30 ºC for 10 mins. Further EPR experiment (**Figure S57**, bottom) indicated the generation of the benzenethiyl radical.[11]

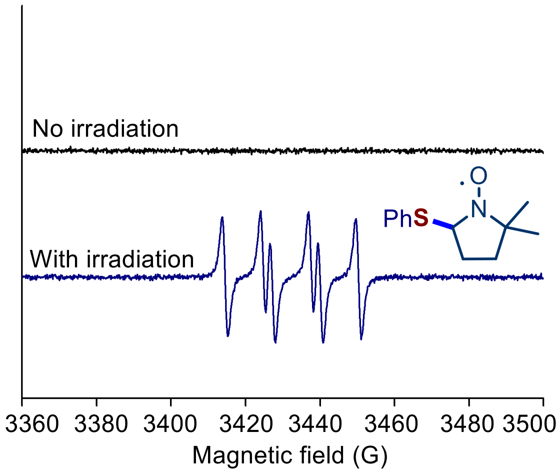

Figure S57. EPR spectra for detection of phenylthiyl radicals

(d) Detection of the 1,2-dichloroethyl radicals

Hydrogen-atom transfer (HAT) of 1,2-dichloroethane by the chloride radical generated the 1,2-dichloroethyl radical (**8**), which was further oxidized to form chloroacetic acid and chlorides. Chloroacetic acid did not serve as the chlorine source for PPS upcycling (**Figure S58**). **8** could be trapped by an chloride radical to form 1,1,2-trichloroethane (**9**) in less than 5% NMR yields, which was also detected by ^{1}H NMR and GC-MS. (**Figure S59**).

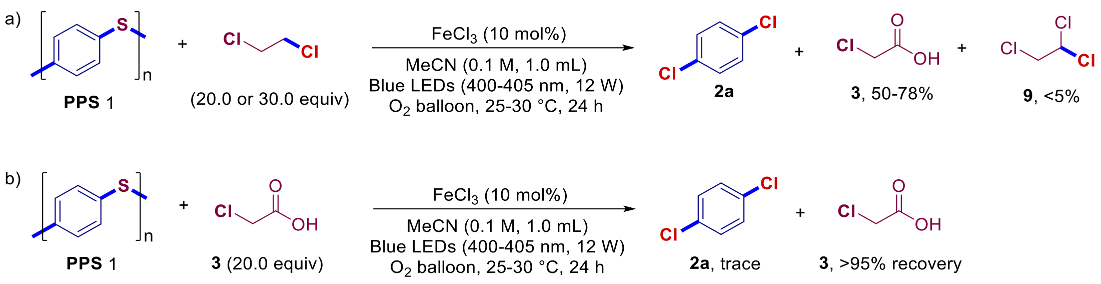

Figure S58. Reaction with **3** and detection of 1,1,2-trichloroethane (**9**)

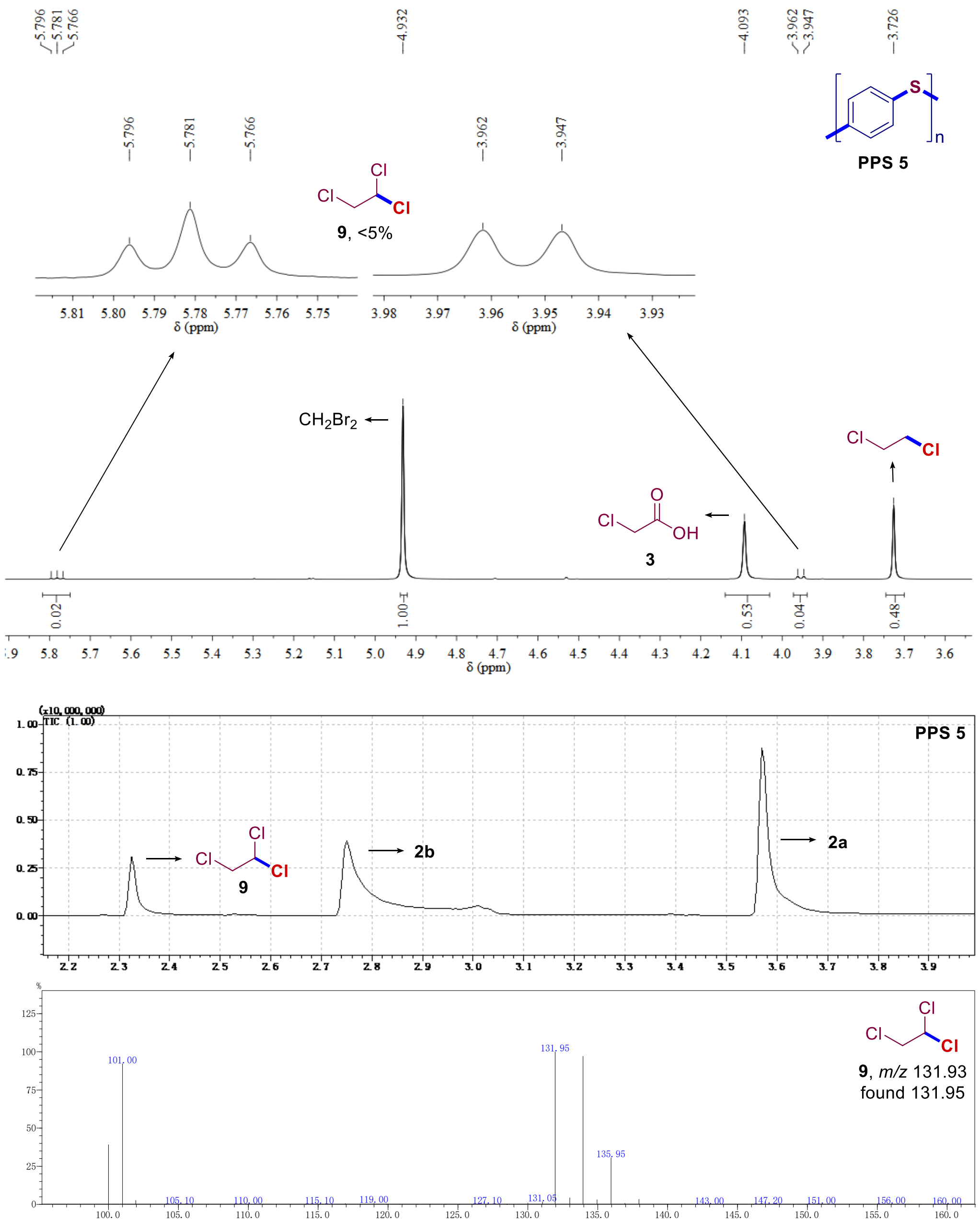

Figure S59. ^{1}H NMR and GC-MS spectra for detection of 1,1,2-trichloroethane (**9**)

(v) The detection of the sulfur species

(a) The capture of sulfur (0) by triphenylphosphine

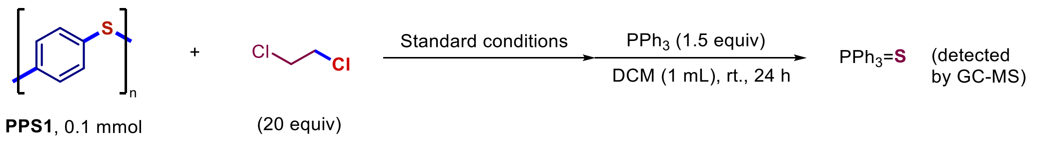

According to GP3, **PPS 1** (10.8 mg, 0.1 mmol PPS) was added. After removing the solvent, triphenylphosphine (0.15 mmol, 39.3 mg) and dichloromethane (1.0 mL) was added. The reaction mixture was stirred at room temperature for another 24 h and analyzed by GC-MS, confirming the formation of triphenylphosphine sulfide, which indicated the existence of sulfur(0) species (**Figure S60**).[12-13]

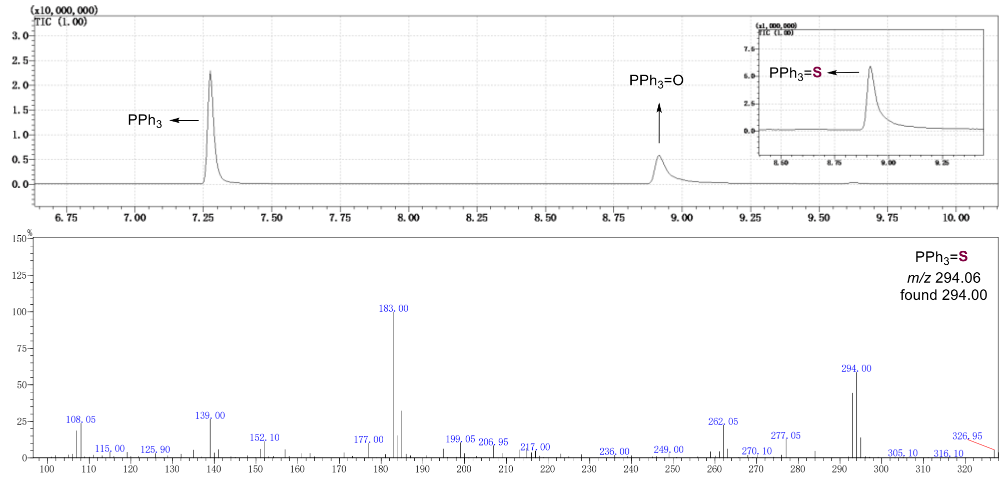

Figure S60. Detection of triphenylphosphine sulfide by GC-MS

(b) The detection of SO_2 by GC-MS

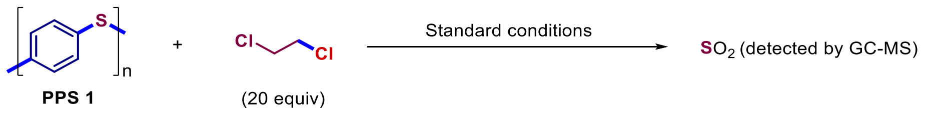

Besides, phenylthiyl radical might be oxidized to provide arylsulfonyl chlorides. Then it is easy to be attacked by chlorine radicals again and extrude sulfur dioxide, and its presence was detected by GC-MS (**Figure S61**).

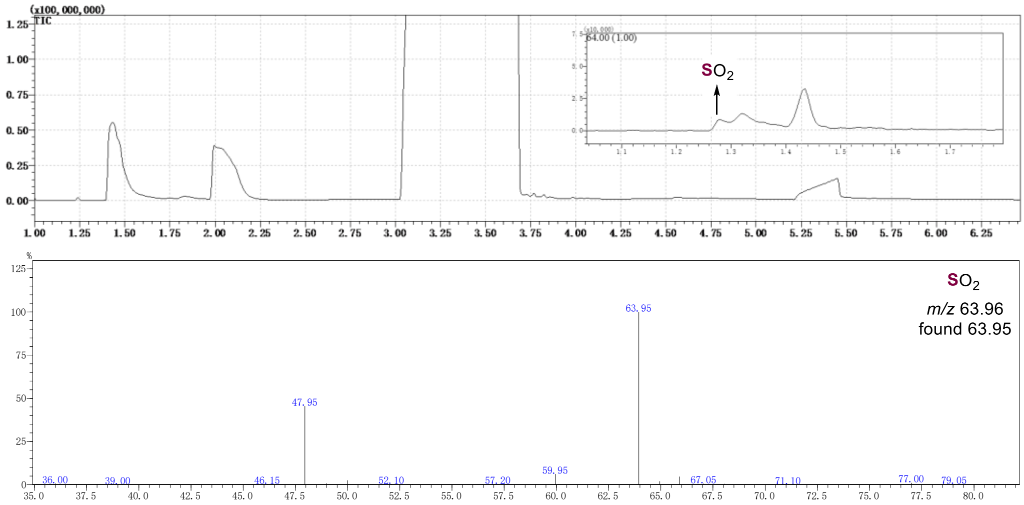

Figure S61. Detection of SO_2 by GC-MS

(c) The precipitation of sulfate ion by $BaCl_2$

According to GP3, **PPS 1** (10.8 mg, 0.1 mmol PPS) was added. The reaction mixture was irradiated under blue LEDs (400-405 nm, 12 W) for the indicated time, then treated with H_2O (1.0 mL). The inorganic layer was separated and treated with acidic $BaCl_2$/HCl solution. For the reaction before light irradiation, no precipitate was observed. The reaction after light irradiation for 24 h yielded withe precipitate immediately.

Blank reaction: $FeCl_3$ (10 mol%, 0.1 M in CH_3CN solution, 1.0 mL) and 1,2-DCE (2.0 mmol, 20 equiv, 160 μL) were added to a 4 mL screw-capped vial. The reaction mixture was irradiated under blue LEDs (400-405 nm, 12 W) for 24 h. After similar treatment with $BaCl_2$/HCl solution, no precipitate was observed. These results indicated that sulfate ion was probably generated.[13-14]

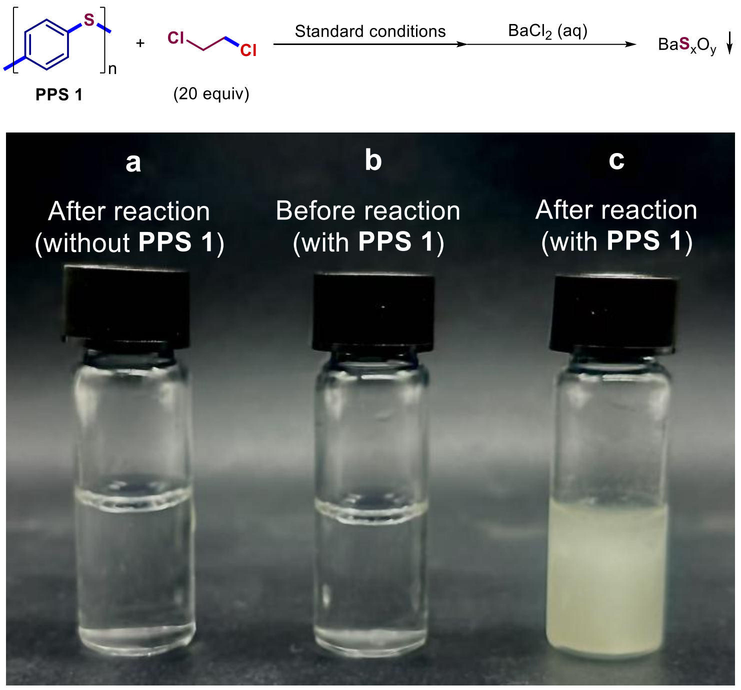

Figure S62. Detection of hypervalent sulfur-containing anion species by $BaCl_2$

(d) Control experiments to probe the possible reaction intermediates

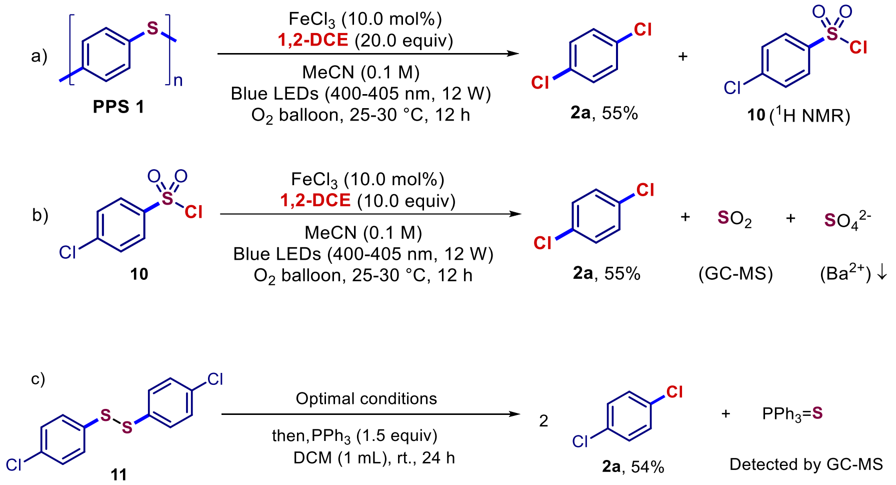

Figure S63. Control experiments to probe the possible reaction intermediates

The detection and reaction of 4-chlorobenzenesulfonyl chloride under the optimized conditions

The reaction mixture of **PPS 1** was stirred under blue LEDs (400-405 nm, 12 W) at 25-30 °C for 12 h, and then analyzed by ^{1}H NMR, confirming the formation of 4-chlorobenzenesulfonyl chloride (**10**) (**Figure S64**). Furthermore, **10** (42.0 mg, 0.2 mmol) reacted with 1,2-DCE (1.0 mmol, 10 equiv, 0.08 mL) successfully to give **2a**

in 95% GC yield under the iron-photocatalysis in only 6 hours, with the detection of SO_2 and sulfate ions (**Figure S63**). These results demonstrated that 4-chlorobenzenesulfonyl chloride, generated from chlorine radical mediated oxidation of *p*-chlorobenzenethiyl radicals, underwent photoinduced desulfonylative chlorination to give **2a**.

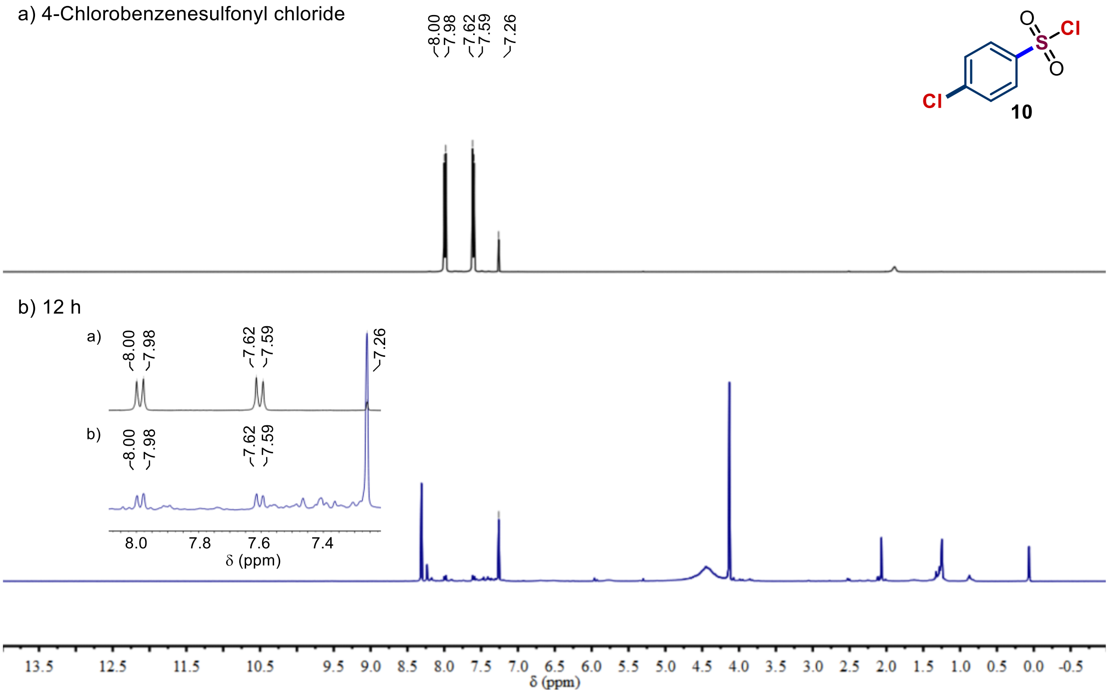

Figure S64. ^{1}H NMR spectra for the detection of **10**

The reaction of 1,2-bis(4-chlorophenyl)disulfane (**11**) under the optimized conditions

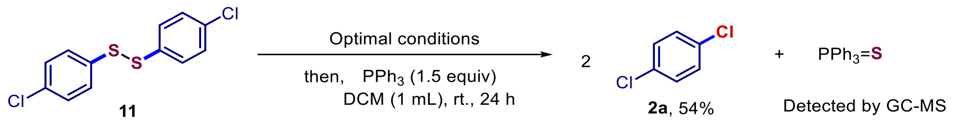

1,2-bis(4-chlorophenyl)disulfane **11** (28.6 mg, 0.1 mmol), $FeCl_3$ (2 mol%, 0.02 M in CH_3CN solution, 1.0 mL), 1,2-DCE (2.0 mmol, 20 equiv, 160 μL) were added to a 4 mL screw-capped vial. The reaction mixture was stirred under blue LEDs (400-405 nm, 12 W) at 25-30 °C for 24 h, affording **2a** in 54% GC yield. Further treatment with triphenylphosphine (0.15 mmol, 39.3 mg) in dichloromethane (1.0 mL) for 24 hours also gave triphenylphosphine sulfide as detected by GC-MS, which suggested that diaryldisulfanes, generated from dimerization of phenylthiyl radical, might serve as

the intermediates.

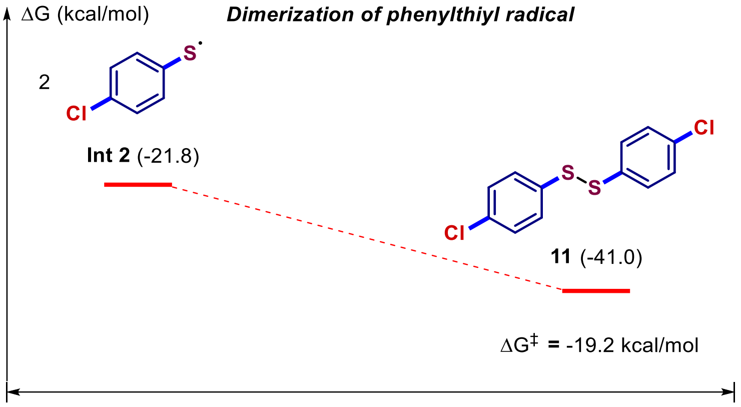

Figure S65. DFT calculations for phenylthiyl radical and diaryldisulfanes **11**

(vi) Proposed catalytic cycle

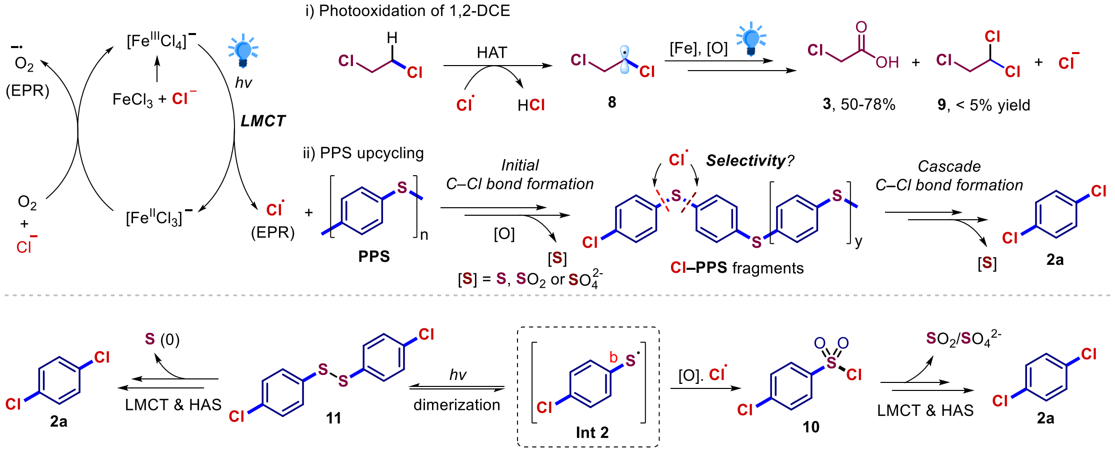

Figure S66. Regioselectivity in the chemical upcycling of PPS

DFT calculations

All calculations were performed with the Gaussian 16 package[15] and ORCA program.[16] Geometry optimizations were performed with PBE0-D3(BJ)[17] set with the PCM(MeCN)[18] solvation model. The def2-SVP[19] basis set was used for all atoms. All optimized structures were verified by frequency calculations and only one imaginary frequency was found in the transition states, while the other structures had no imaginary frequency. Besides, the thermal correction to Gibbs free energy were

obtained after frequency calculations. Intrinsic Reaction Coordinate (IRC)[20] were utilized to confirm the reaction pathway. In addition, the single point calculations considering solvation effect were performed with PWPB95-D3(BJ)[21] and def2-TZVPP basis set with the SMD(MeCN)[22] solvation model. All optimized structures were visualized using CYLview20.[23]

Cartesian coordinates for optimized structure

ClPhCl

C	0.00000000	0.69613200	1.21315000
C	0.00000000	-0.69613000	1.21315000
C	0.00000000	-1.38354500	0.00000000
C	0.00000000	-0.69613000	-1.21315000
C	0.00000000	0.69613200	-1.21315000
C	0.00000000	1.38354800	0.00000000
H	0.00000000	1.24706800	2.15489600
H	0.00000000	-1.24706200	2.15489900
H	0.00000000	-1.24706200	-2.15489900
H	0.00000000	1.24706800	-2.15489600
Cl	0.00000000	-3.11757200	0.00000000
Cl	0.00000000	3.11757400	0.00000000

ClPhSPh

C	2.84665100	0.91331900	0.61798900
C	1.58706200	1.50752700	0.64177800
C	0.51651500	0.92808400	-0.04763800
C	0.71780400	-0.26042200	-0.76074500
C	1.96734000	-0.86990700	-0.77105200
C	3.02752600	-0.27625000	-0.08436300
H	3.68180900	1.36349800	1.15704000
H	1.43469100	2.42714300	1.21131500

H	-0.10863400	-0.71781400	-1.30866500
H	2.12491000	-1.79911500	-1.32119300
Cl	4.58875000	-1.03278000	-0.10345300
S	-1.03713900	1.78272400	-0.05145700
C	-2.21318000	0.45450400	0.03836400
C	-3.31598700	0.48154500	-0.82075000
C	-2.09133200	-0.56692100	0.98898800
C	-4.29466000	-0.50850800	-0.72590500
H	-3.40085000	1.27424600	-1.56766500
C	-3.06037000	-1.56338700	1.06062000
H	-1.23463300	-0.58133600	1.66656900
C	-4.16669500	-1.53506500	0.20811300
H	-5.15499000	-0.48144100	-1.39882700
H	-2.95690700	-2.36331600	1.79770800
H	-4.92816900	-2.31557800	0.27280800

Int-1

C	-0.63977100	-1.20498800	-1.15788900
C	0.50665400	-1.03540300	-0.41574700
C	0.41697600	-0.78533900	0.97456600
C	-0.86137500	-0.75534000	1.59311500
C	-2.00374300	-0.94154500	0.84589700
C	-1.90864700	-1.11933200	-0.54487400
H	-0.56897900	-1.36802400	-2.23552500
H	1.48300000	-1.09083100	-0.89858300
H	-0.93238500	-0.59709300	2.67131300
H	-2.97941600	-0.90703500	1.33170300
S	1.78846100	-0.72820800	2.03587500
Cl	-0.20734500	1.99594600	0.52985600
C	3.14542100	-0.34363900	0.96519900

C	4.27165700	-1.17031700	0.98420100
C	3.12482800	0.81670500	0.18111900
C	5.38387100	-0.84449800	0.21293500
H	4.28017200	-2.07417100	1.59693100
C	4.22944000	1.13504600	-0.60046500
H	2.23407800	1.45567900	0.17877500
C	5.35161300	0.30421200	-0.57688800
H	6.26877700	-1.48242700	0.21664200
H	4.22733500	2.03112600	-1.22311800
S	-3.30390700	-1.22300800	-1.58619800
C	-4.50526000	-0.22554200	-0.74699700
C	-4.17217100	1.04393600	-0.25225800
C	-5.81966600	-0.69562400	-0.67465100
C	-5.16343500	1.82571300	0.33276900
H	-3.14244900	1.40915200	-0.30679400
C	-6.80596300	0.10684100	-0.10251100
H	-6.06635600	-1.68797400	-1.05859800
C	-6.47983100	1.36369100	0.40499800
H	-4.90551300	2.81112700	0.72754900
H	-7.83308000	-0.26023500	-0.04501600
H	-7.25294400	1.98718800	0.85953300
Cl	6.72766000	0.70751900	-1.54691800

Ts-1

C	-0.74181900	-0.99712800	-1.30715200
C	0.36319100	-0.18526600	-1.25524900
C	0.31402300	1.07101800	-0.54524600
C	-0.99253500	1.55753400	-0.16956800
C	-2.08267800	0.72449600	-0.21179800
C	-1.97407500	-0.57851200	-0.75085400
H	-0.67057600	-1.97193700	-1.79623500

H	1.31084200	-0.49936900	-1.69633800
H	-1.07666800	2.57253700	0.22289800
H	-3.04764700	1.08543800	0.14789700
S	1.13316200	0.56421000	1.41176200
Cl	1.43087300	2.30253700	-1.14863200
C	2.76489800	0.12053500	0.96314900
C	3.07649100	-1.20433600	0.61138200
C	3.78494000	1.08765200	0.93496400
C	4.36892900	-1.55705200	0.23858600
H	2.29075500	-1.96230400	0.63460900
C	5.08146300	0.74412400	0.57002600
H	3.54904400	2.11859000	1.20461900
C	5.36324500	-0.57764100	0.22062400
H	4.61055100	-2.58543900	-0.03498500
H	5.87403400	1.49394700	0.55237300
Cl	6.97691800	-1.01114800	-0.24065600
S	-3.29891700	-1.72213000	-0.82858500
C	-4.65457700	-0.82440400	-0.11111200
C	-4.89156100	-0.90817000	1.26443100
C	-5.50086400	-0.07278600	-0.93204100
C	-5.97247500	-0.22510100	1.81923200
H	-4.22600300	-1.50326100	1.89337400
C	-6.58001100	0.60664900	-0.36927000
H	-5.30773900	-0.02016500	-2.00561500
C	-6.81514500	0.53136400	1.00403700
H	-6.15756100	-0.28634900	2.89401500
H	-7.24100800	1.19659300	-1.00820700
H	-7.66235900	1.06434000	1.44176900

Int-2

S	3.16421500	0.00000000	-0.00000100
C	1.45295200	0.00000100	0.00000400
C	0.72452400	-1.21770200	0.00000000
C	0.72452400	1.21770300	-0.00000100
C	-0.65931400	-1.22130700	0.00000000
H	1.27558100	-2.16017300	-0.00000300
C	-0.65931400	1.22130800	0.00000000
H	1.27558000	2.16017400	-0.00000400
C	-1.34573000	0.00000100	0.00000700
H	-1.21770800	-2.15857700	-0.00000400
H	-1.21770700	2.15857900	-0.00000400
Cl	-3.06880200	0.00000000	-0.00000900

Int-3

C	-3.49184200	1.71088500	0.11857300
C	-2.46139100	0.90813700	-0.33028900
C	-2.74683000	-0.32465500	-0.94321500
C	-4.09890200	-0.76176600	-1.02673500
C	-5.14238500	0.08590500	-0.61659000
C	-4.83242900	1.30017500	-0.03762000
H	-3.27697900	2.67814300	0.57590500
H	-1.42837700	1.24566600	-0.23500000
H	-4.33251200	-1.69162700	-1.54806100
H	-6.17957900	-0.23142900	-0.72707500
S	-1.53485300	-1.34067800	-1.66842800
Cl	-3.73745100	-2.11877600	1.11410800
C	-0.09282800	-0.96012800	-0.71659500
C	1.07438900	-0.54723900	-1.36121000
C	-0.10069200	-1.14446600	0.67378900
C	2.23306100	-0.31028000	-0.62619500

H	1.07946600	-0.40158100	-2.44363200
C	1.04923900	-0.89429600	1.40678500
H	-1.01653900	-1.47976700	1.16969400
C	2.22754700	-0.47746400	0.76301700
H	3.13954300	0.01434600	-1.13914300
H	1.03900500	-1.03311100	2.49090400
Cl	-6.09241300	2.34622800	0.50939500
S	3.63301100	-0.19768500	1.79664800
C	4.88107900	0.31339000	0.63952500
C	5.03979700	1.67074700	0.34189900
C	5.72380100	-0.63962500	0.05838400
C	6.03509700	2.07067000	-0.54814900
H	4.37922500	2.40625800	0.80605400
C	6.71718200	-0.23230800	-0.83057900
H	5.59357800	-1.69609800	0.30261800
C	6.87231100	1.12066400	-1.13435900
H	6.15798500	3.13041500	-0.78296600
H	7.37426700	-0.97641000	-1.28650000
H	7.65247400	1.43705600	-1.83060000

Ts-2

C	3.78122700	-0.23823300	1.37995900
C	2.67136000	-0.22010700	0.57695500
C	2.74622800	0.23496800	-0.80702400
C	4.08595200	0.50328300	-1.32455700
C	5.18303400	0.47952700	-0.50440700
C	5.03850800	0.12551900	0.85327000
H	3.70369400	-0.54975000	2.42281600
H	1.69668700	-0.51558400	0.96957200
H	4.18574100	0.76252000	-2.38092800

H	6.17245900	0.71854800	-0.89727500
S	1.76735800	-0.66215800	-2.02186700
Cl	1.93290500	2.12558700	-0.67544100
C	0.22553700	-0.90706900	-1.18421600
C	-0.03552900	-2.12329500	-0.54118000
C	-0.75572100	0.08957300	-1.19965400
C	-1.25484300	-2.33358900	0.09038400
H	0.72556700	-2.90654500	-0.53389700
C	-1.97605200	-0.11487600	-0.56389600
H	-0.55314500	1.03659600	-1.70197000
C	-2.23368100	-1.32775900	0.08688300
H	-1.44894900	-3.28567900	0.59068100
H	-2.73092600	0.67273800	-0.57945700
Cl	6.42179100	0.10141600	1.87234100
S	-3.74465100	-1.69315000	0.92979500
C	-4.71764800	-0.23226000	0.65216800
C	-4.69577100	0.80730800	1.58730800
C	-5.52946700	-0.14247800	-0.48333400
C	-5.47931900	1.94122700	1.37807000
H	-4.06086400	0.72413100	2.47202700
C	-6.30842400	0.99504700	-0.68735800
H	-5.54233100	-0.96314000	-1.20384100
C	-6.28345700	2.03589500	0.24179700
H	-5.46119300	2.75423900	2.10745500
H	-6.94016700	1.06706800	-1.57564700
H	-6.89661400	2.92558300	0.08045700

Int-4

C	-3.97731300	-0.79571800	-1.08599100
C	-2.78519000	-0.34925600	-0.58990500

C	-2.70964800	0.73937800	0.40573700
C	-4.02107400	1.26524400	0.84740300
C	-5.19700300	0.80003300	0.33055900
C	-5.19295500	-0.22834100	-0.63946700
H	-3.99882500	-1.59779900	-1.82587900
H	-1.84353100	-0.78498100	-0.92979800
H	-4.01495000	2.05621400	1.60071900
H	-6.14776300	1.21709300	0.66728900
S	-1.78280400	0.25716900	1.92259100
Cl	-1.79696600	2.16443900	-0.42810200
C	-0.24167600	-0.32223500	1.27110700
C	-0.04748500	-1.68787200	1.02511500
C	0.80884600	0.57241000	1.03893200
C	1.17084400	-2.14795800	0.54189800
H	-0.86066900	-2.39188600	1.21443200
C	2.03050100	0.11736600	0.55325500
H	0.66372500	1.63574000	1.23669700
C	2.21917200	-1.24665800	0.29951800
H	1.31114200	-3.21524300	0.35269400
H	2.83955500	0.82751300	0.37509600
S	3.72549800	-1.93512000	-0.32038800
C	4.78697600	-0.51588700	-0.45148300
C	4.84987300	0.19860600	-1.65204900
C	5.58409600	-0.14087200	0.63492700
C	5.70378300	1.29528900	-1.75917100
H	4.22570200	-0.10621700	-2.49481100
C	6.43458200	0.95746400	0.52130000
H	5.52991600	-0.70983600	1.56558500
C	6.49435000	1.67504800	-0.67400400
H	5.75201400	1.85484300	-2.69613500

H	7.05538700	1.25223700	1.37044800
H	7.16312200	2.53452800	-0.76106500
Cl	-6.68383800	-0.80277900	-1.27522400

Ts-3

C	-3.27640700	1.60724300	0.68673100
C	-2.53632200	0.46664700	0.90236100
C	-3.01780300	-0.81755900	0.44706200
C	-4.40185500	-0.89388600	0.03466300
C	-5.12031100	0.25474400	-0.18995300
C	-4.55345700	1.51036900	0.10975300
H	-2.88327000	2.58315300	0.97589100
H	-1.54833300	0.52000800	1.36319800
H	-4.83252700	-1.87609500	-0.16732200
H	-6.14189400	0.20122700	-0.56976000
S	-2.01787300	-1.04749900	-1.45956300
Cl	-2.44236800	-2.19571800	1.39383300
C	-0.34867600	-1.13439300	-0.95629300
C	0.43776600	0.02693400	-0.87103300
C	0.23867400	-2.37030500	-0.62659200
C	1.76750300	-0.03610500	-0.47246900
H	-0.00741700	0.99121400	-1.12608100
C	1.56693900	-2.44088300	-0.23457900
H	-0.36296100	-3.27902500	-0.68798300
C	2.34361300	-1.27276000	-0.15231400
H	2.35979600	0.87837700	-0.41344500
H	2.01114800	-3.40964300	0.00860100
S	4.02026700	-1.47346900	0.36646900
C	4.68008800	0.17112100	0.23193000
C	5.21113500	0.61096800	-0.98523900

C	4.70249800	1.00553500	1.35368000
C	5.75142800	1.89194300	-1.08035500
H	5.19426200	-0.05232200	-1.85278300
C	5.24770500	2.28476200	1.25210300
H	4.28838000	0.64957100	2.29946500
C	5.76999700	2.72820700	0.03682800
H	6.16345100	2.23688400	-2.03147800
H	5.26411700	2.93771400	2.12772600
H	6.19645500	3.73109600	-0.03992700
Cl	-5.47007400	2.94098200	-0.17691600

Int-5

C	0.64569400	-0.51175600	0.00001700
C	1.87848800	-1.13530100	0.00000600
C	3.08771300	-0.38919600	-0.00001200
C	2.98540900	1.02957700	-0.00001700
C	1.75756000	1.65673200	-0.00000500
C	0.56908800	0.89381500	0.00001100
H	-0.26755100	-1.10873300	0.00003000
H	1.93894300	-2.22577900	0.00001100
H	3.90382900	1.62031400	-0.00003100
H	1.70155700	2.74825400	-0.00001000
S	4.60569500	-1.16656400	-0.00002600
S	-0.93345300	1.79311600	0.00002700
C	-2.17287700	0.51955100	0.00001300
C	-2.67121100	0.03742100	-1.21416500
C	-2.67123600	0.03741900	1.21418100
C	-3.66267200	-0.94214900	-1.20949200
H	-2.27716200	0.42730800	-2.15509200
C	-3.66269700	-0.94215100	1.20948500

H	-2.27720600	0.42730300	2.15511600
C	-4.15695100	-1.43178200	-0.00000900
H	-4.05158100	-1.32313800	-2.15645000
H	-4.05162600	-1.32314100	2.15643500
H	-4.93478800	-2.19884200	-0.00001800

ClPhSSPhCl

S	0.57803800	0.84246400	2.29400400
C	-0.10477600	1.87286300	1.00741600
C	0.45418900	3.15109600	0.89043500
C	-1.11327900	1.47001700	0.13255200
C	0.00597300	4.02130700	-0.09769000
H	1.24453500	3.47504800	1.57260600
C	-1.56584300	2.33963500	-0.85671500
H	-1.55513500	0.47546800	0.22501800
C	-1.00388200	3.60962200	-0.96750800
H	0.43756300	5.01903000	-0.19172000
H	-2.35457800	2.02851200	-1.54360400
Cl	-1.56584300	4.69204000	-2.20101800
S	-0.57803800	-0.84246400	2.29400400
C	0.10477600	-1.87286300	1.00741600
C	-0.45418900	-3.15109600	0.89043500
C	1.11327900	-1.47001700	0.13255200
C	-0.00597300	-4.02130700	-0.09769000
H	-1.24453500	-3.47504800	1.57260600
C	1.56584300	-2.33963500	-0.85671500
H	1.55513500	-0.47546800	0.22501800
C	1.00388200	-3.60962200	-0.96750800
H	-0.43756300	-5.01903000	-0.19172000
H	2.35457800	-2.02851200	-1.54360400

Cl 1.56584300 -4.69204000 -2.20101800

References

1. R. S. Aal E Ali, Y. Zhou, K. Gong, X. Jiang, *Green Synth. Catal.* **2023**, *4*, 169-172.

2. W. Deng, Y. Zou, Y. Wang, L. Liu, Q. Guo, *Synlett* **2004**, *7*, 1254-1258.

3. Rábai, *Synthesis* **1989**, *7*, 523-525.

4. I. Güell, and X. Ribas, *Eur. J. Org. Chem.* **2014**, *15*, 3188-3195.

5. A. Augurusa, M. Mehta, M. Perez, J. Zhu, and D. W. Stephan, *Chem. Commun.* **2016**, *52*, 12195-12198.

6. M. J. Westa, A. J. B. Watson, Org. Biomol. Chem. **2019**, *17*, 5055-5059.

7. Y. Fan, S. Pitie, C. Liu, C. Zhao, C. Zhao, M. Seydou, Y. J. Dappe, R. J. Nichols, L. Yang, *J. Phys. Chem. C* **2022**, *126*, 3635-3645.

8. R. N. Salvatore, R. A. Smith, A. K. Nischwitz, T. A. Gavin, *Tetrahedron Lett.* **2005**, *46*, 8931-8935.

9. K. H. Seo, L. S. Park, J. B. Baek, W. Brostow, *Polymer* **1993**, *34*, 12.

10. C. J. Stacy, *J. Appl. Polym. Sci.* **1986**, *32*, 3959.

11. Markushyna, Y., C. M. Schgüßlbauer, T. Ullrich, D. M. Guldi, M. Antonietti, A. Savateev, *Angew. Chem. Int. Ed.* **2021**, *60*, 20543-20550.

12. J. Shen, J. Li, M. Chen, X. Yue, X. Shi, *Org. Lett.* **2024**, *26*, 1495–1500.

13. B. Gu, Y. Zhao, C. Li, X. Jiang, *Green Chem.* **2025**, *27*, 3851-3857.

14. R. Huang, Y. Zhao, C. Li, D. Li, X. Jiang, *Nat. Sustain.* **2025**, *8*, 818-826.

15. M. J. Frisch, Gaussian, Inc. Wallingford CT. **2016**.

16. F. Neese, *Comput Mol Sci.* **2022**, *12*, 1606.

17. (a) J. P. Perdew, K. Burke, M. Ernzerhof, *Phys. Rev. Lett.* **1996**, *77*, 3865-3868. (b) J. P. Perdew, K. Burke, M. Ernzerhof, *Phys. Rev. Lett.* **1997**, *78*, 1396. (c) C. Adamo, V. Barone, *J. Chem. Phys.* **1999**, *110*, 6158-6169. (d) M. Ernzerhof, G. E. Scuseria, *J. Chem. Phys.* **1999**, *110*, 5029-5036. (e) S. Grimme, S. Ehrlich, L. Goerigk, *J. Comput.*

Chem. **2011**, *32*, 1456-1465.

18. (a) S. Miertuš, E. Scrocco, J. Tomasi, *Chem. Phys.* **1981**, *55*, 117-129. (b) S. Miertuš, J. Tomasi, *Chem. Phys.* **1982**, *65*, 239-245. (c) J. L. Pascual-Ahuir, E. Silla, I. Tuñon, *Comp. Chem.* **1994**, *15*, 1127-1138. (d) J. Tomasi, B. Mennucci, R. Cammi, *Chem. Rev.* **2005**, *105*, 2999-3093.

19. (a) A. Schaefer, H. Horn, R. Ahlrichs, *Chem. Phys.* **1992**, *97*, 2571-2577. (b) A. Schaefer, C. Huber, R. Ahlrichs, *J. Chem. Phys.* **1994**, *100*, 5829-5835. (c) F. Weigend, R. Ahlrichs, *Phys. Chem. Chem. Phys.* **2005**, *7*, 3297-3305. (d) F. Weigend, *Phys. Chem. Chem. Phys.* **2006**, *8*, 1057-1065.

20. (a) K. Fukui, *Acc. Chem. Res.* **1981**, *14*, 363-368. (b) H. P. Hratchian, H. B. Schlegel, E. C. E. Dykstra, G. Frenking, K. S. Kim, G. Scuseria, Elsevier, Amsterdam, **2005**, 195-249.

21. L. Goerigk, S. Grimme, *J. Chem. Theory Comput.* **2011**, *7*, 291–309.

22. A. V. Marenich, C. J. Cramer, D. G. Truhlar, *J. Phys. Chem. B* **2009**, *113*, 6378-6396.

23. C. Y. Legault, **2020**, http://www.cylview.org.

NMR spectra

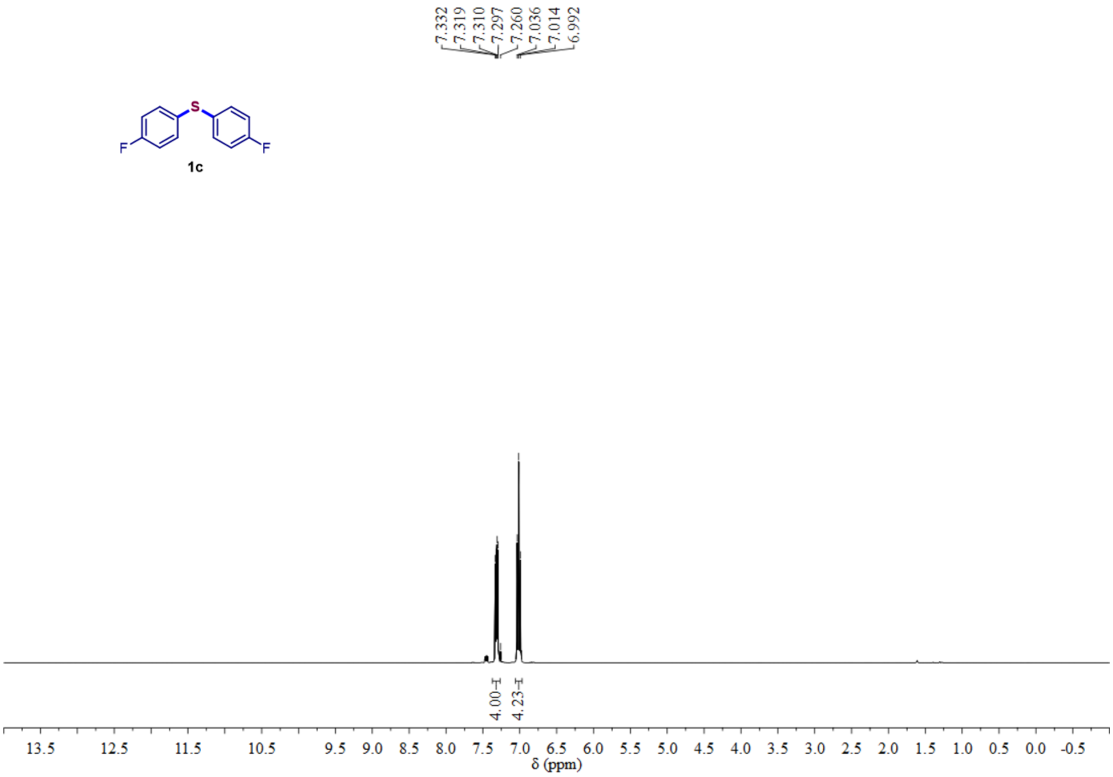

Figure S67. ^{1}H NMR spectra of **1c**

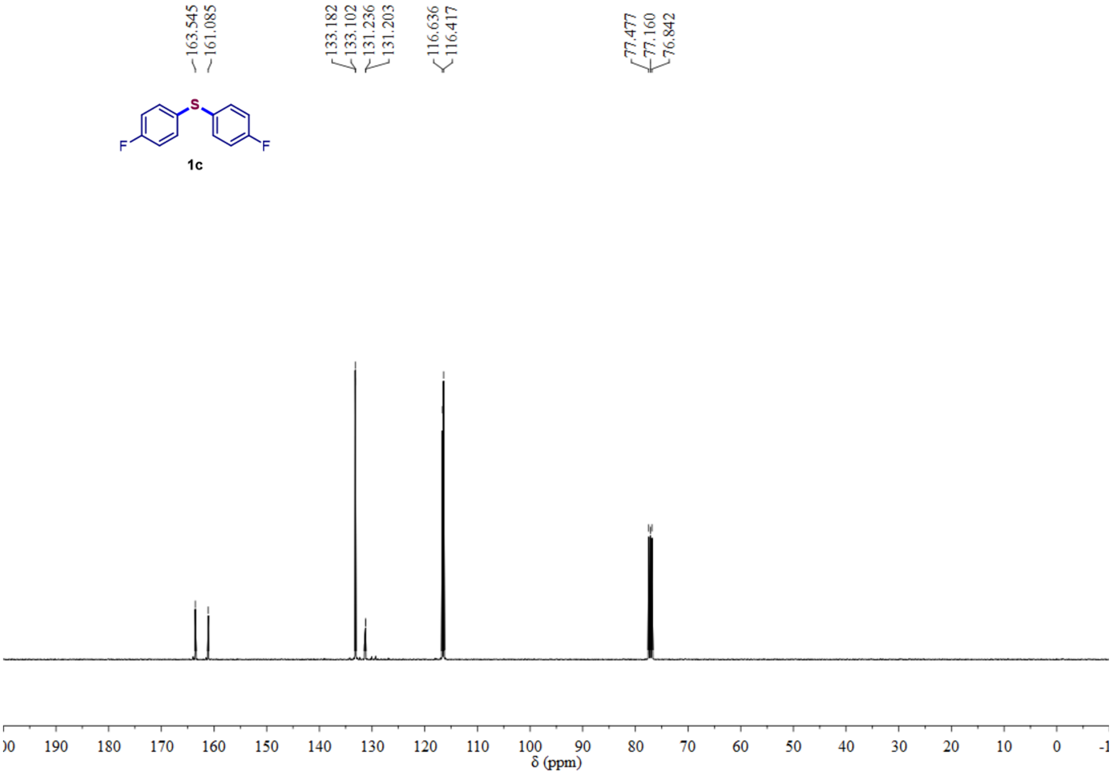

Figure S68. ^{13}C NMR spectra of **1c**

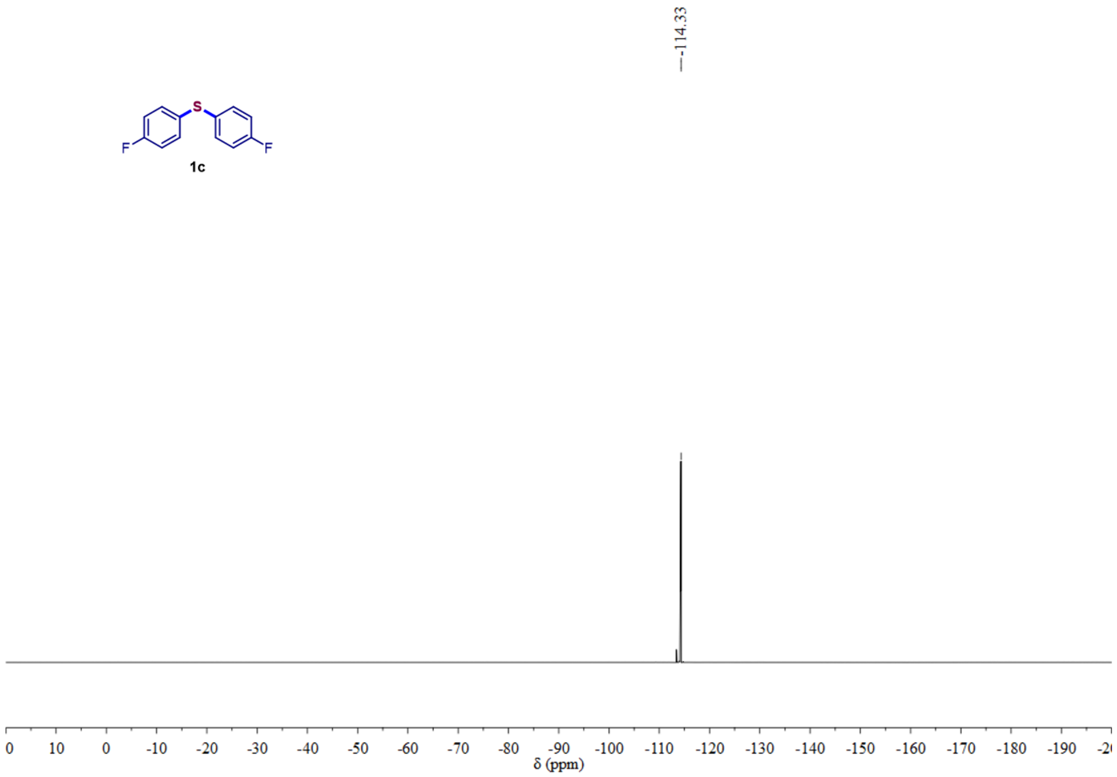

Figure S69. ^{19}F NMR spectra of **1c**

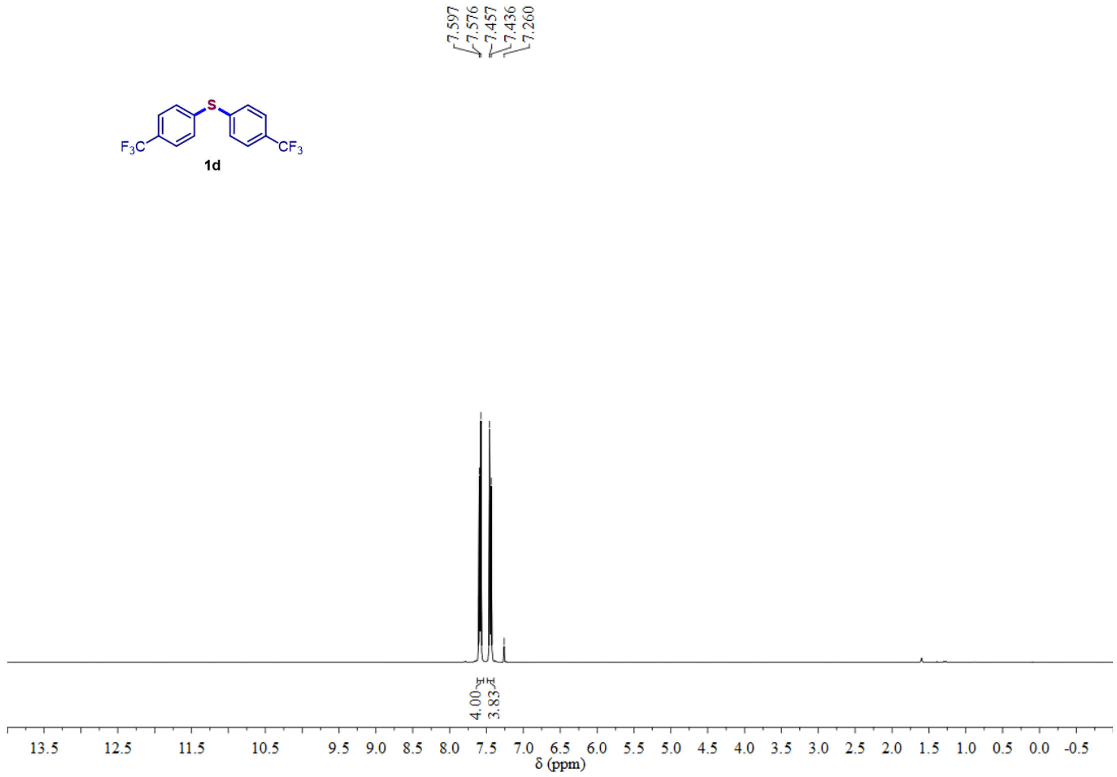

Figure S70. ^{1}H NMR spectra of **1d**

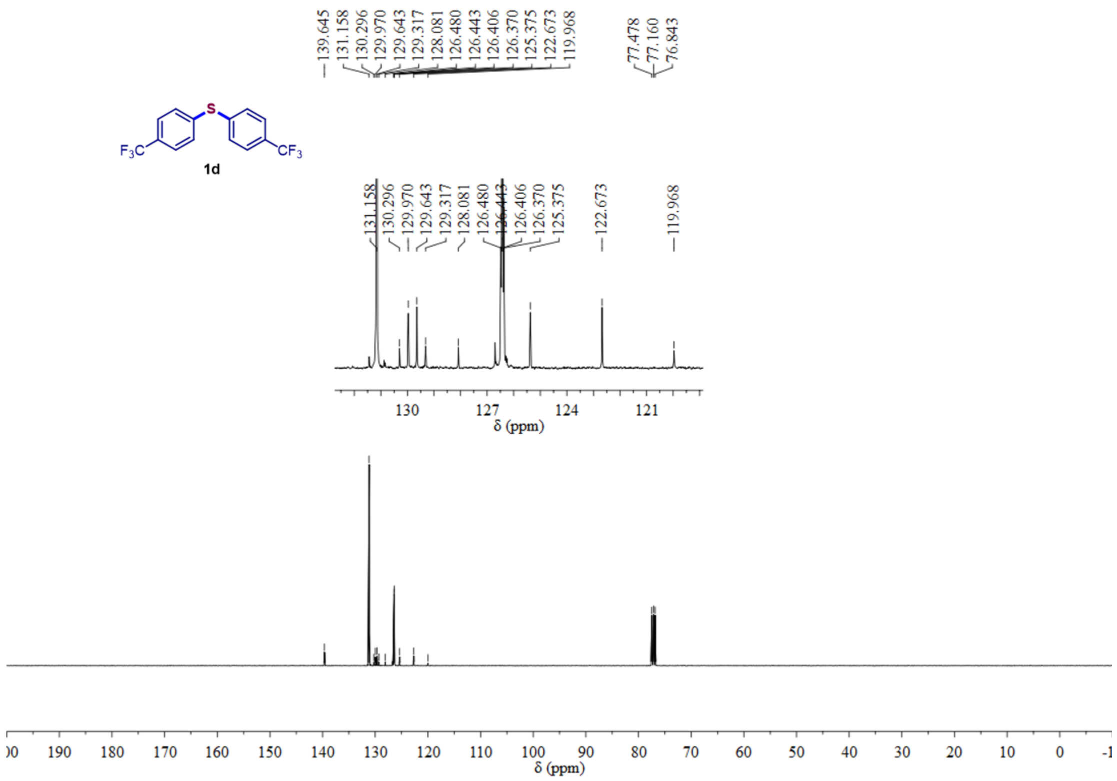

Figure S71. ^{13}C NMR spectra of **1d**

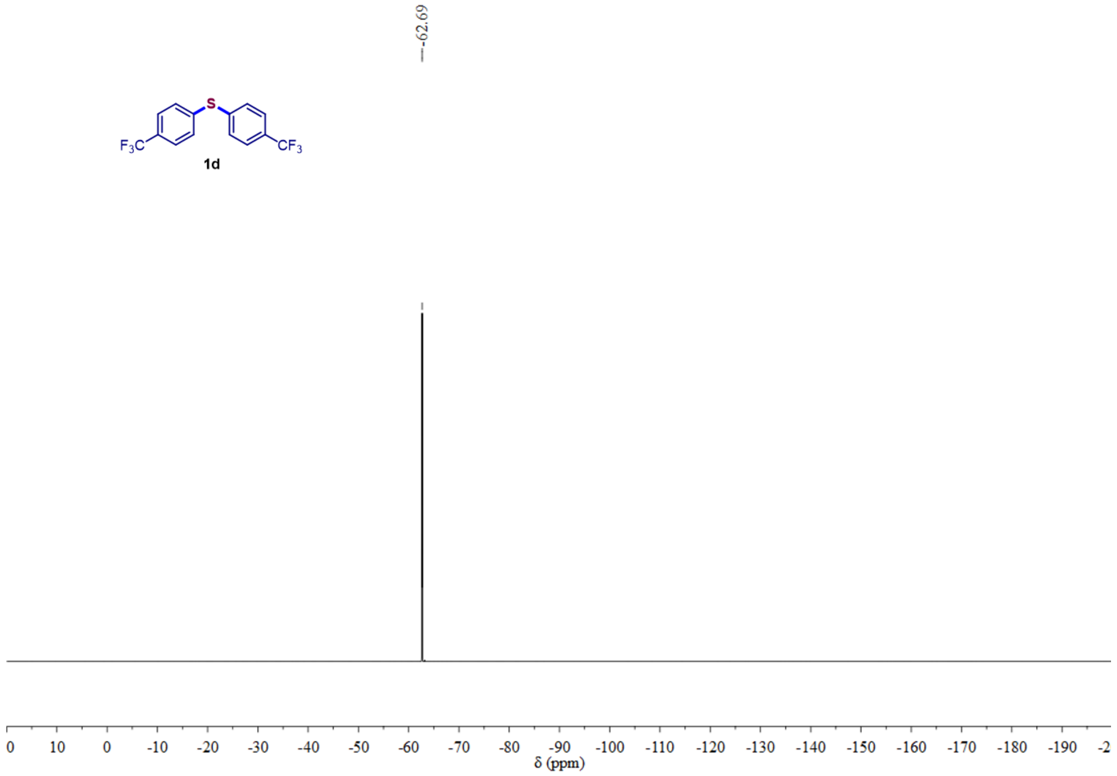

Figure S72. ^{19}F NMR spectra of **1d**

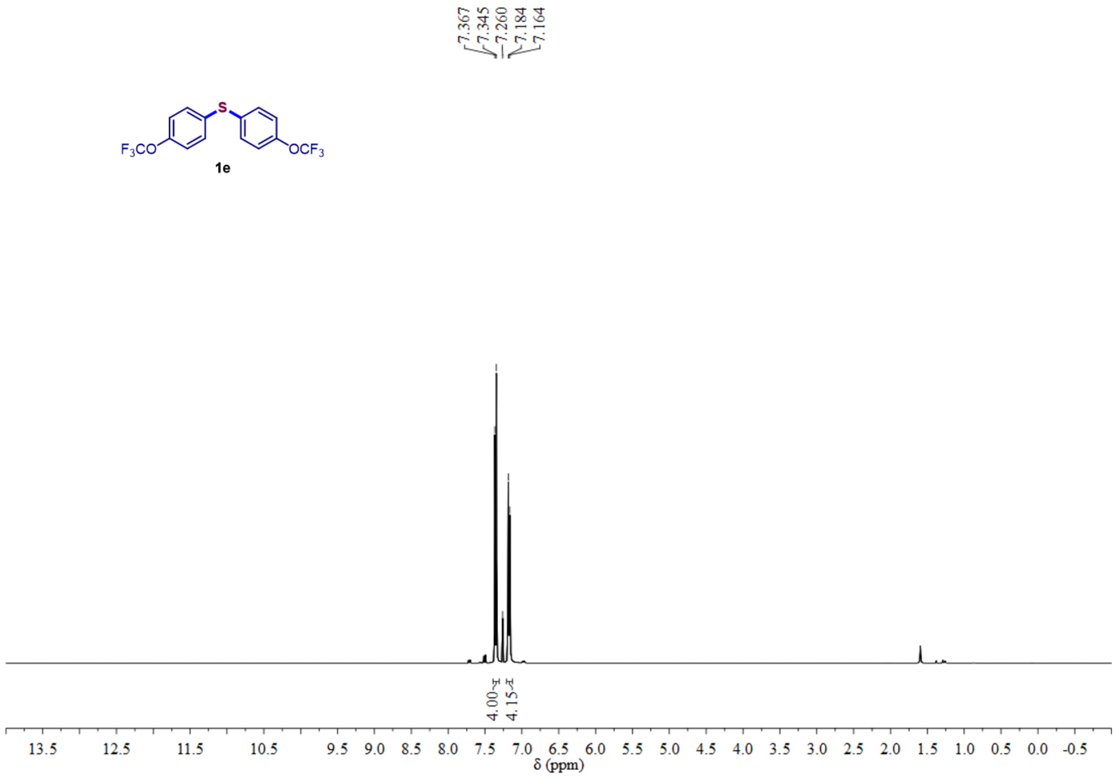

Figure S73. ^{1}H NMR spectra of **1e**

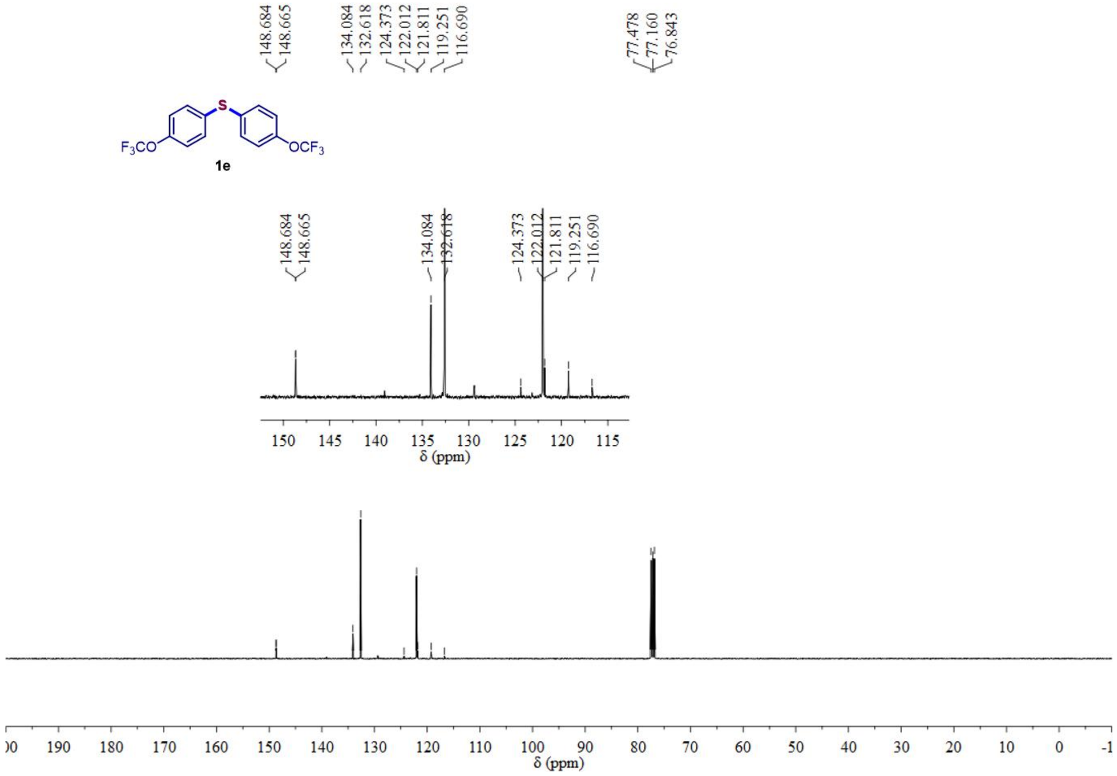

Figure S74. ^{13}C NMR spectra of **1e**

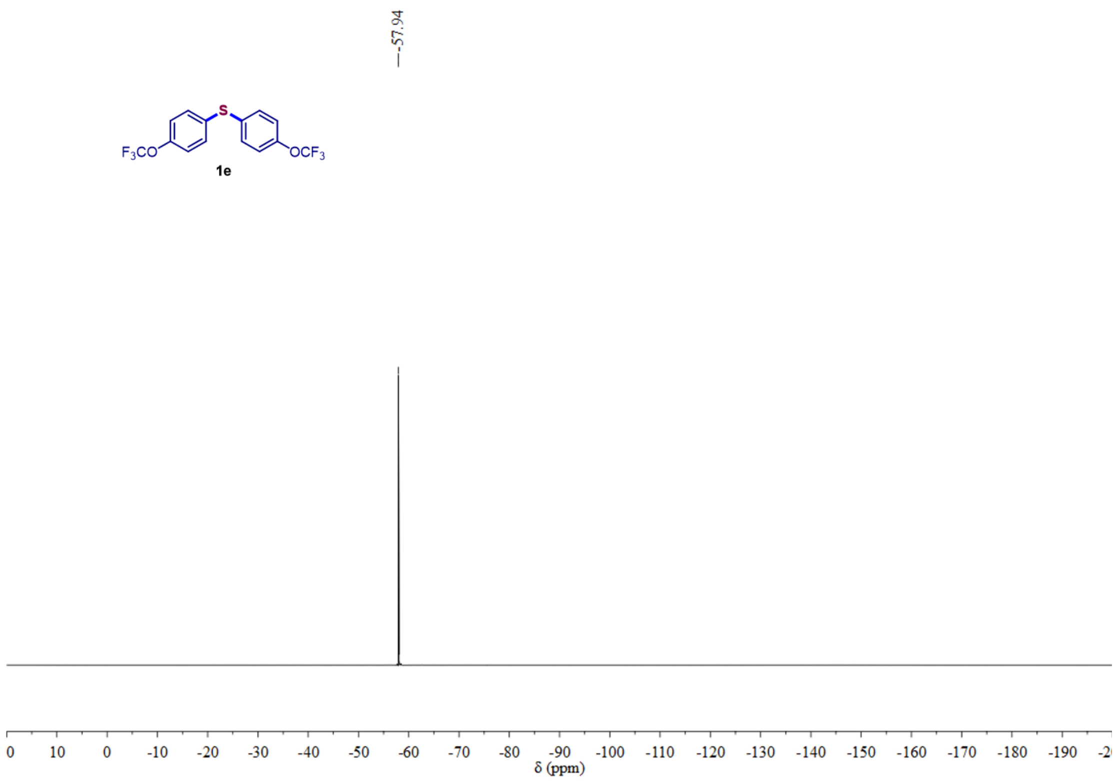

Figure S75. ^{19}F NMR spectra of **1e**

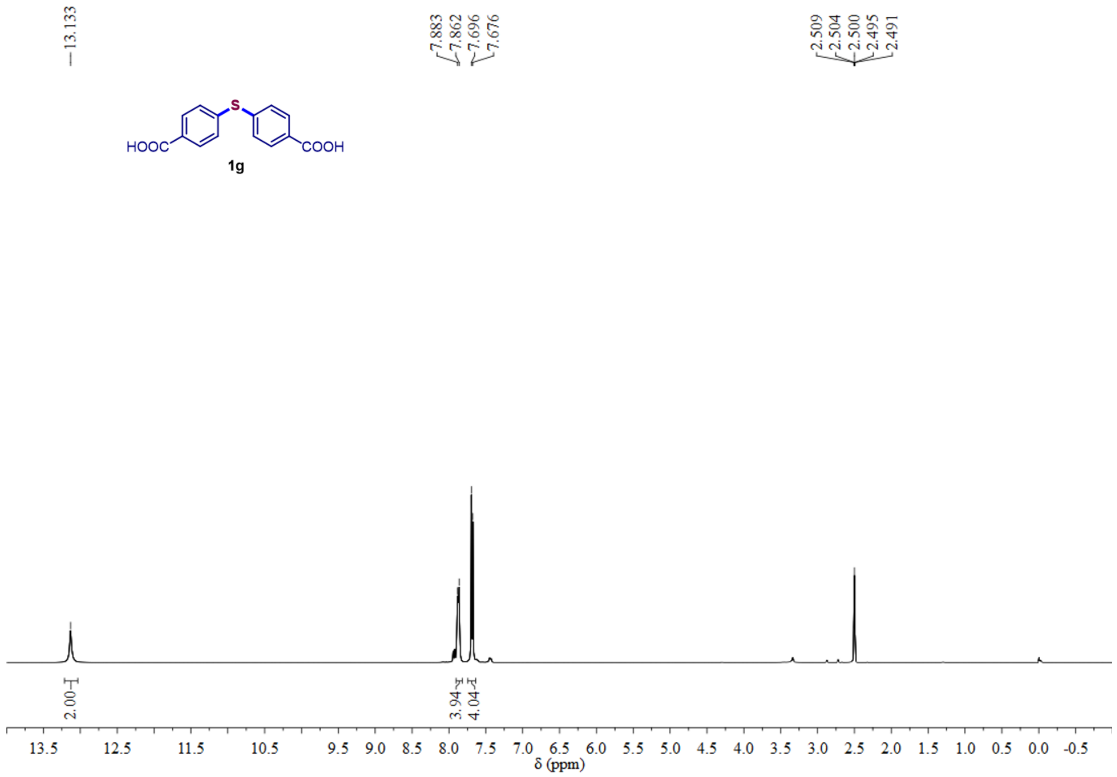

Figure S76. ^{1}H NMR spectra of **1g**

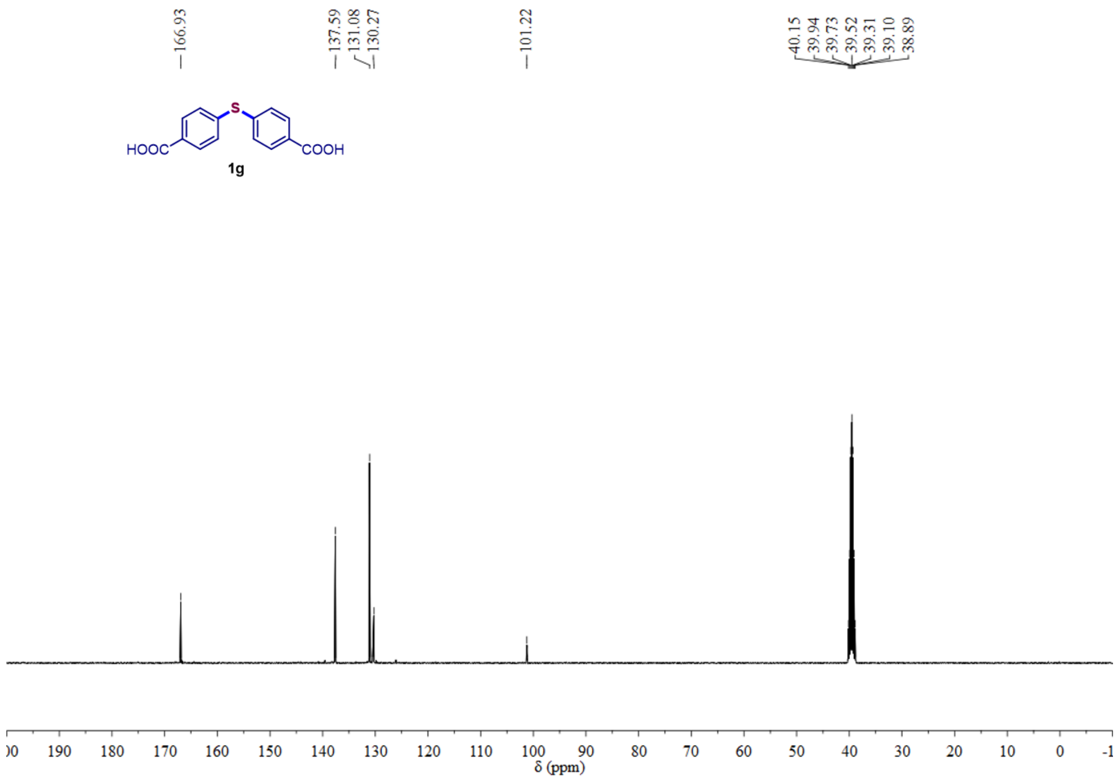

Figure S77. ^{13}C NMR spectra of **1g**

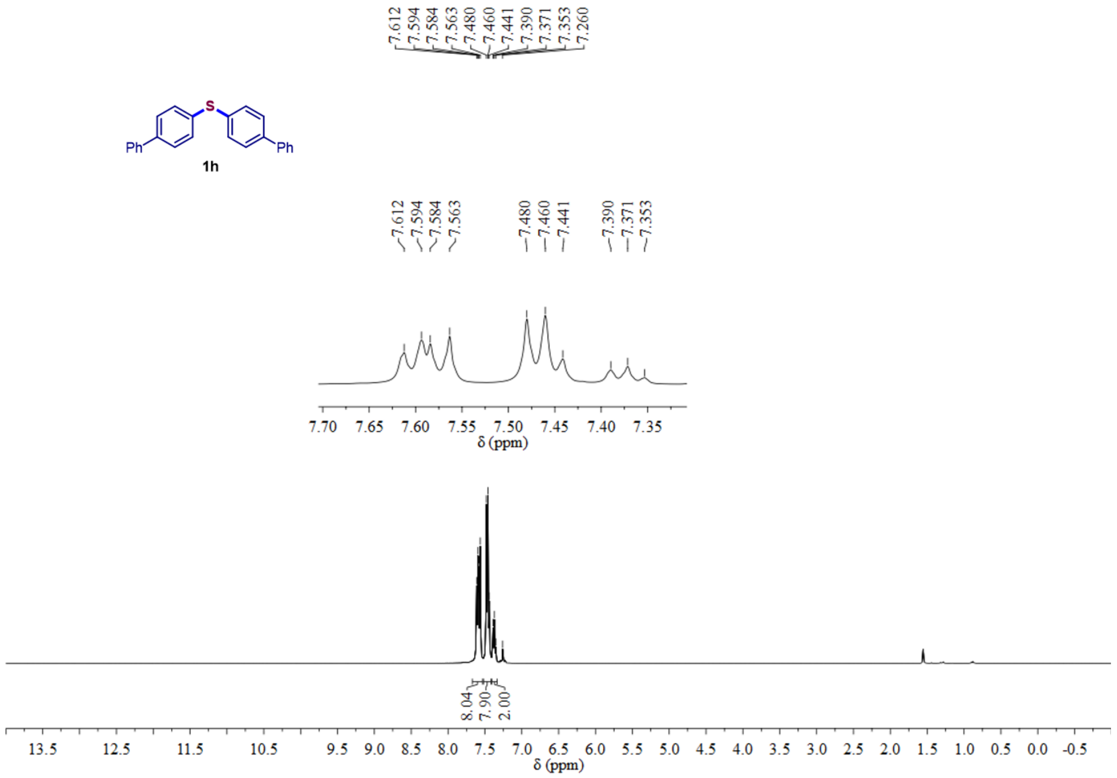

Figure S78. ^{1}H NMR spectra of **1h**

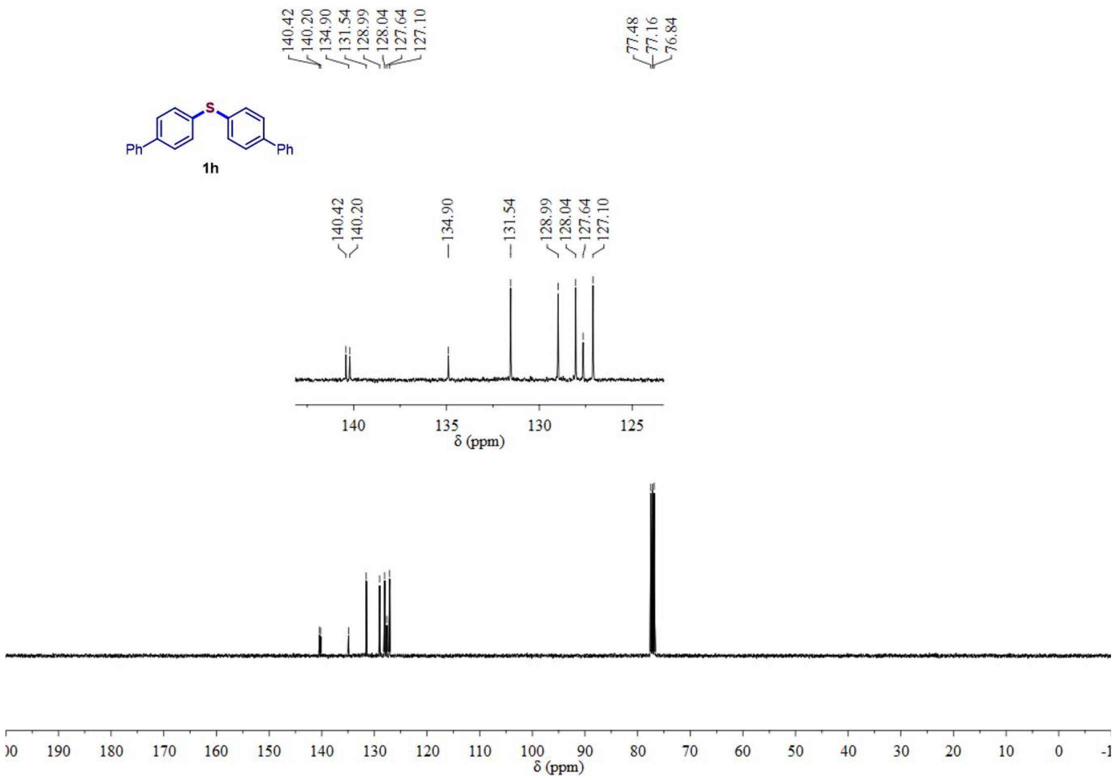

Figure S79. ^{13}C NMR spectra of **1h**

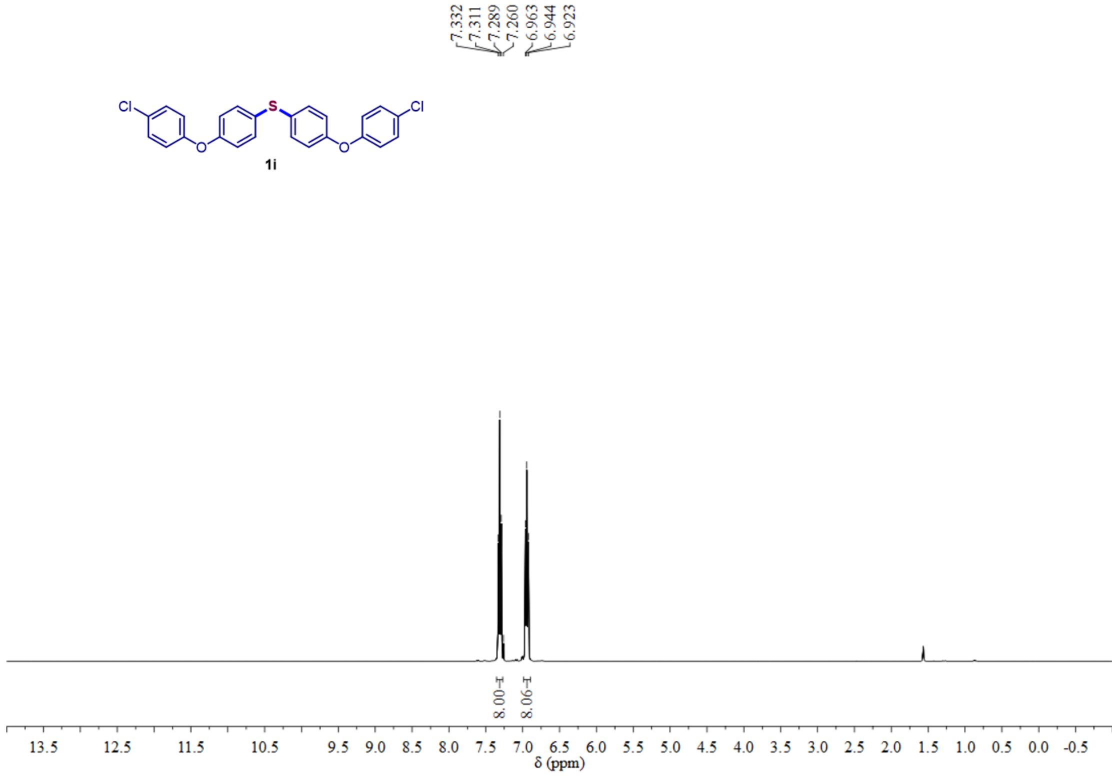

Figure S80. ^{1}H NMR spectra of **1i**

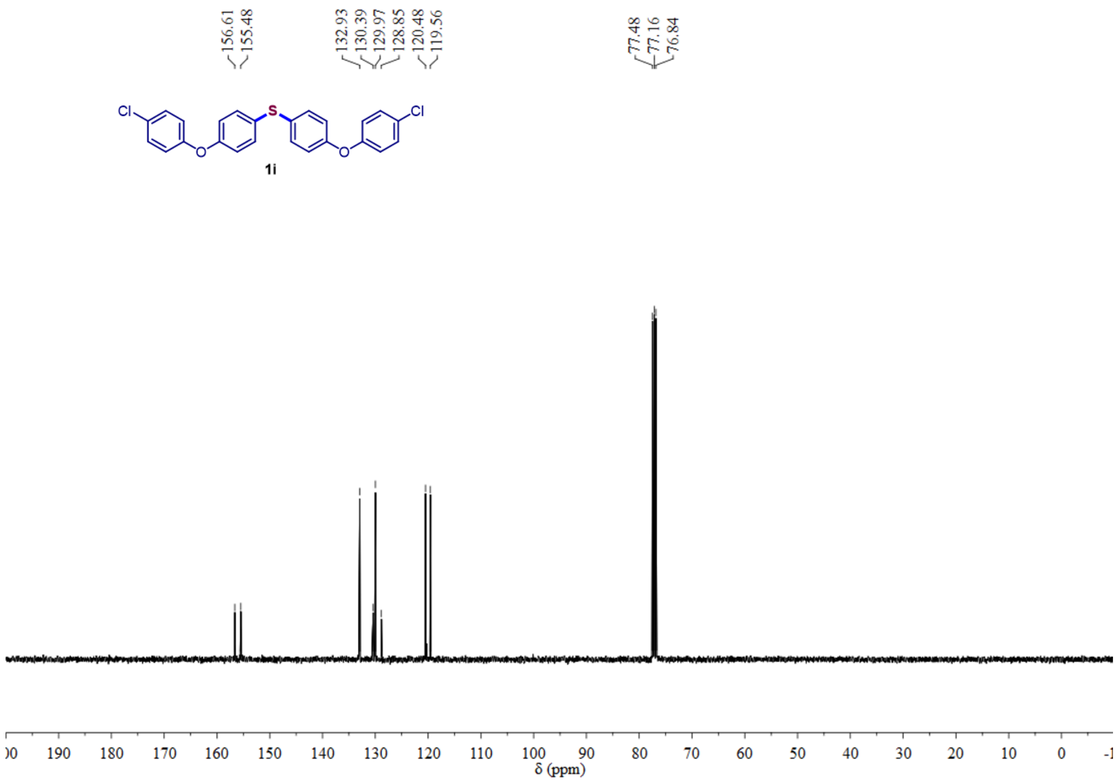

Figure S81. ^{13}C NMR spectra of **1i**

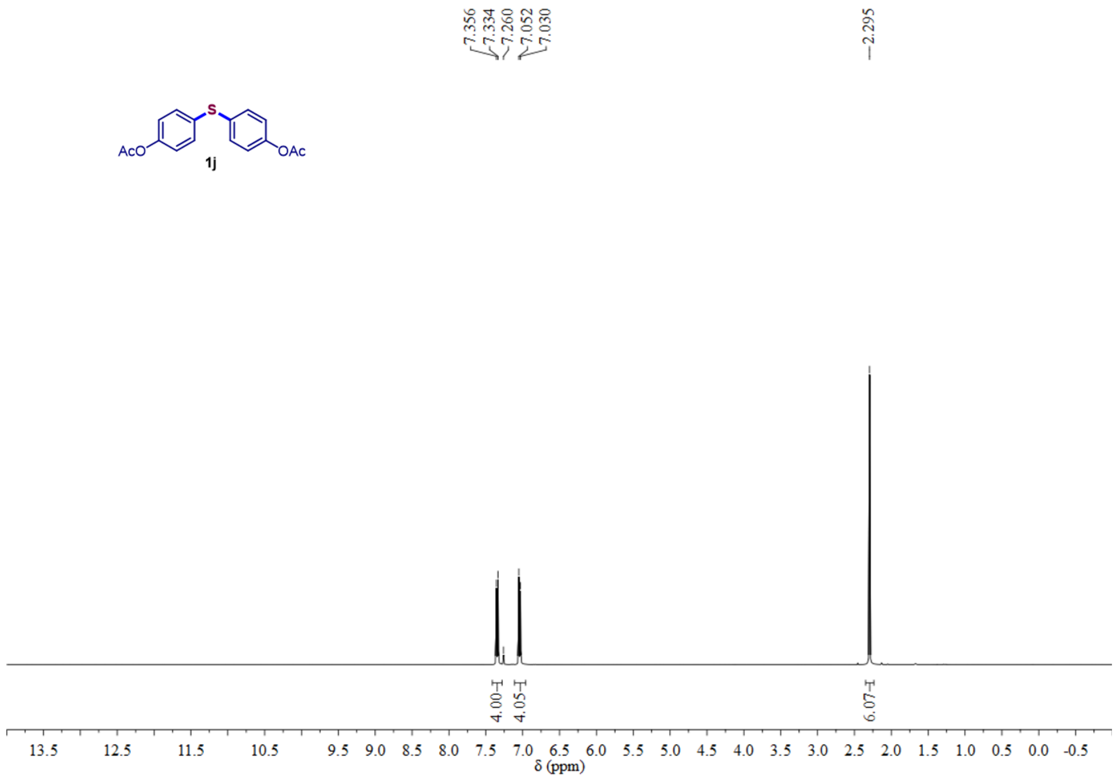

Figure S82. ^{1}H NMR spectra of **1j**

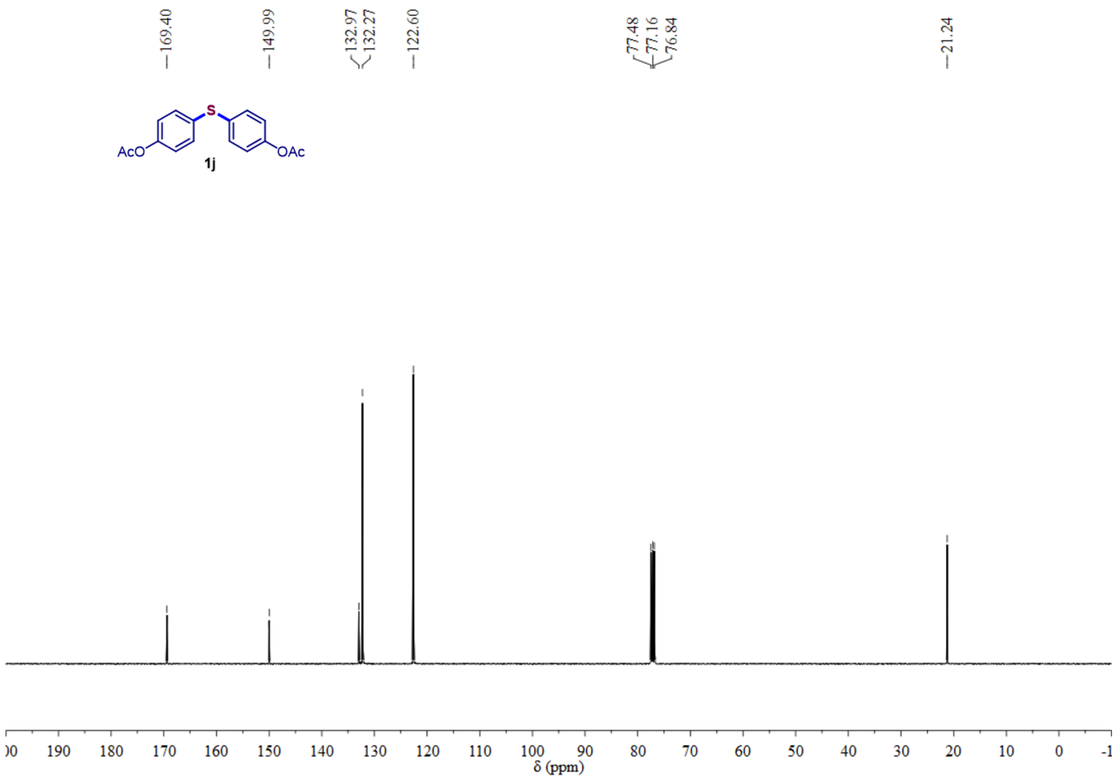

Figure S83. ^{13}C NMR spectra of **1j**

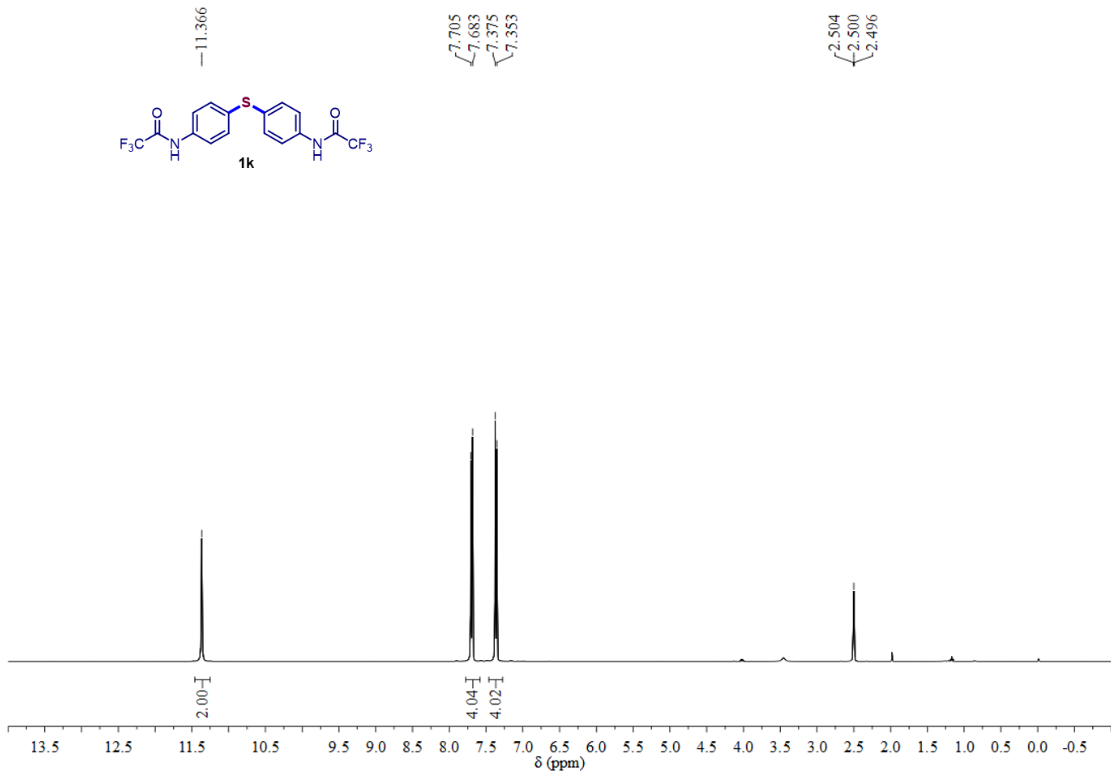

Figure S84. ^{1}H NMR spectra of **1k**

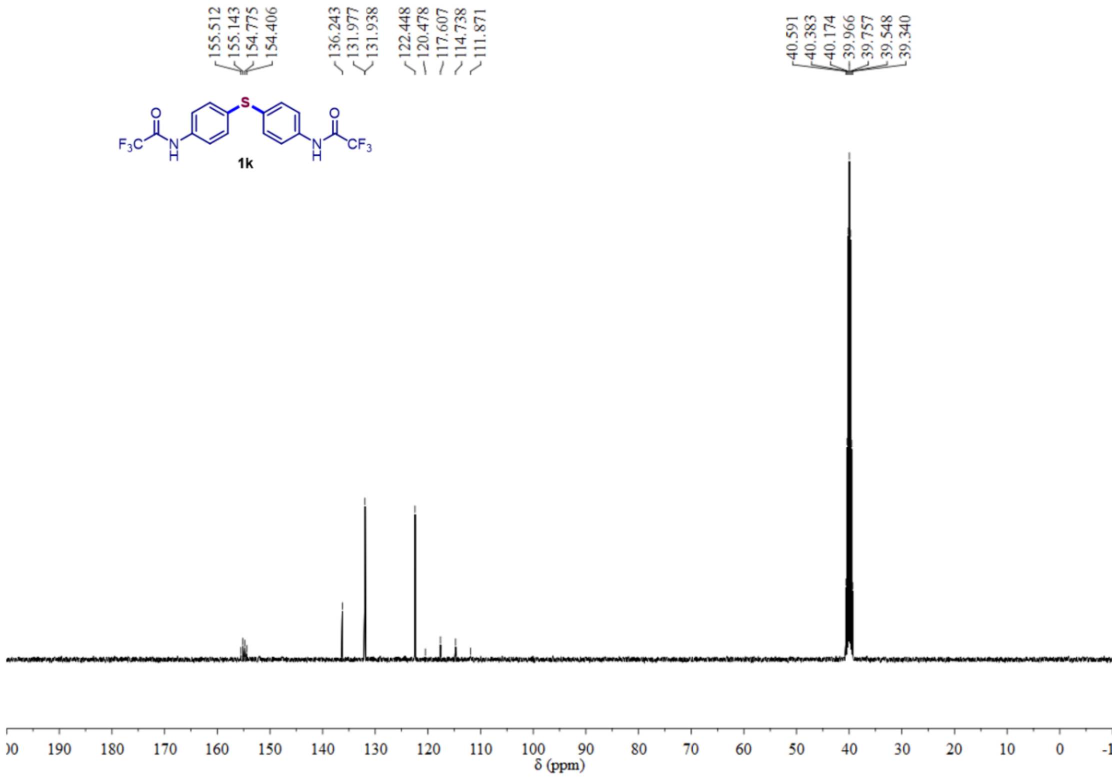

Figure S85. ^{13}C NMR spectra of **1k**

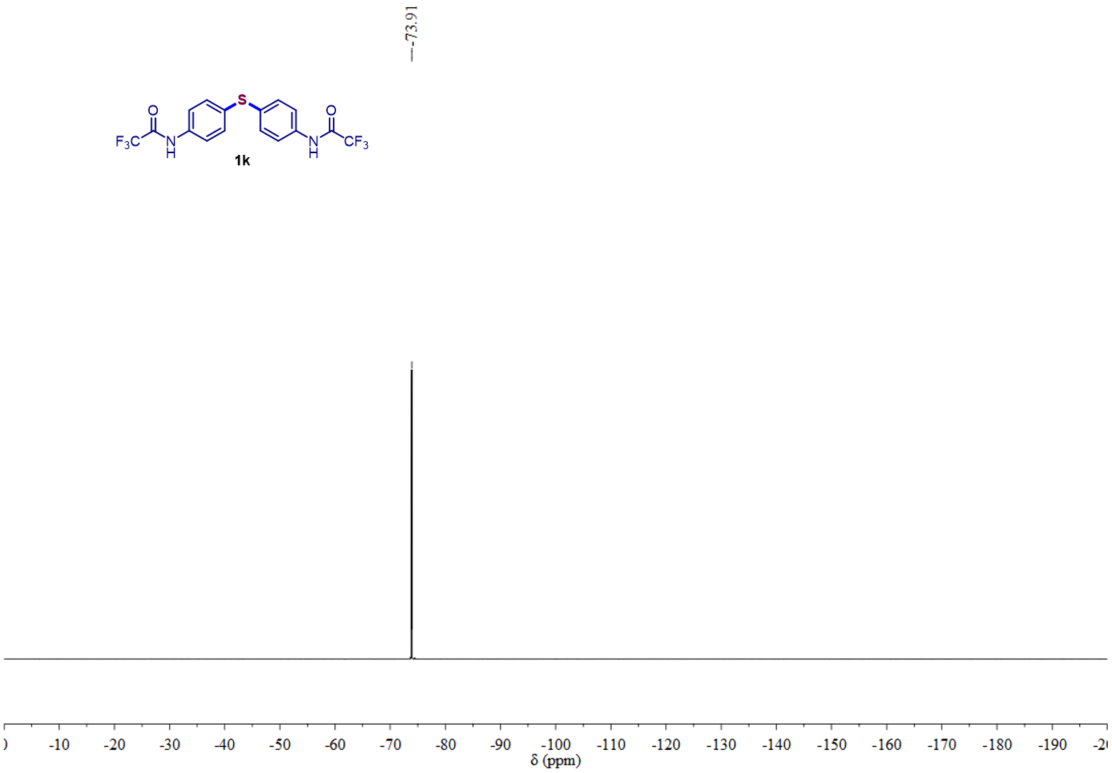

Figure S86. ^{19}F NMR spectra of **1k**

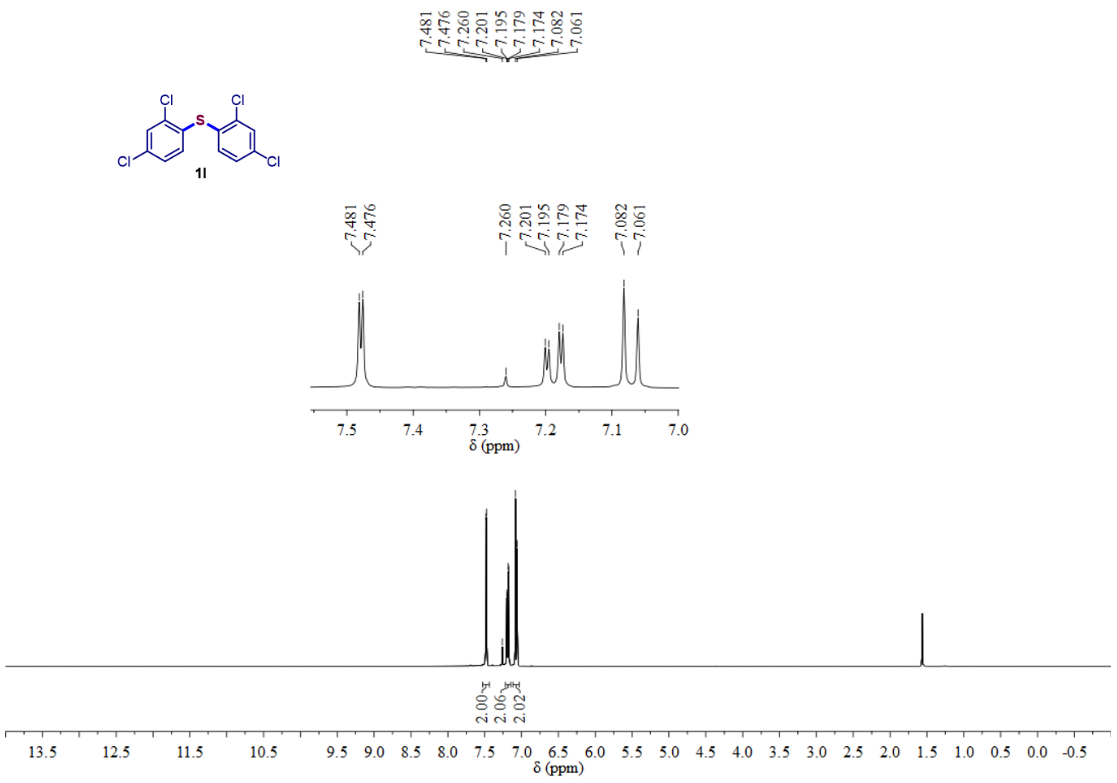

Figure S87. ^{1}H NMR spectra of **1l**

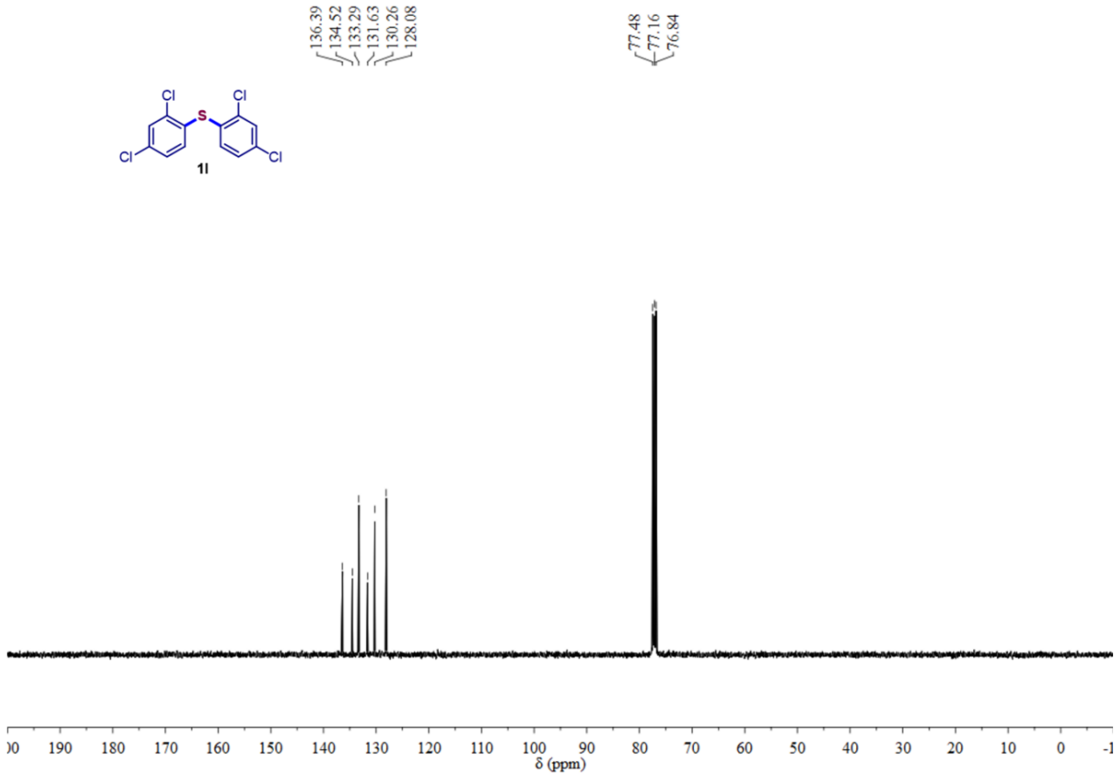

Figure S88. ^{13}C NMR spectra of **1l**

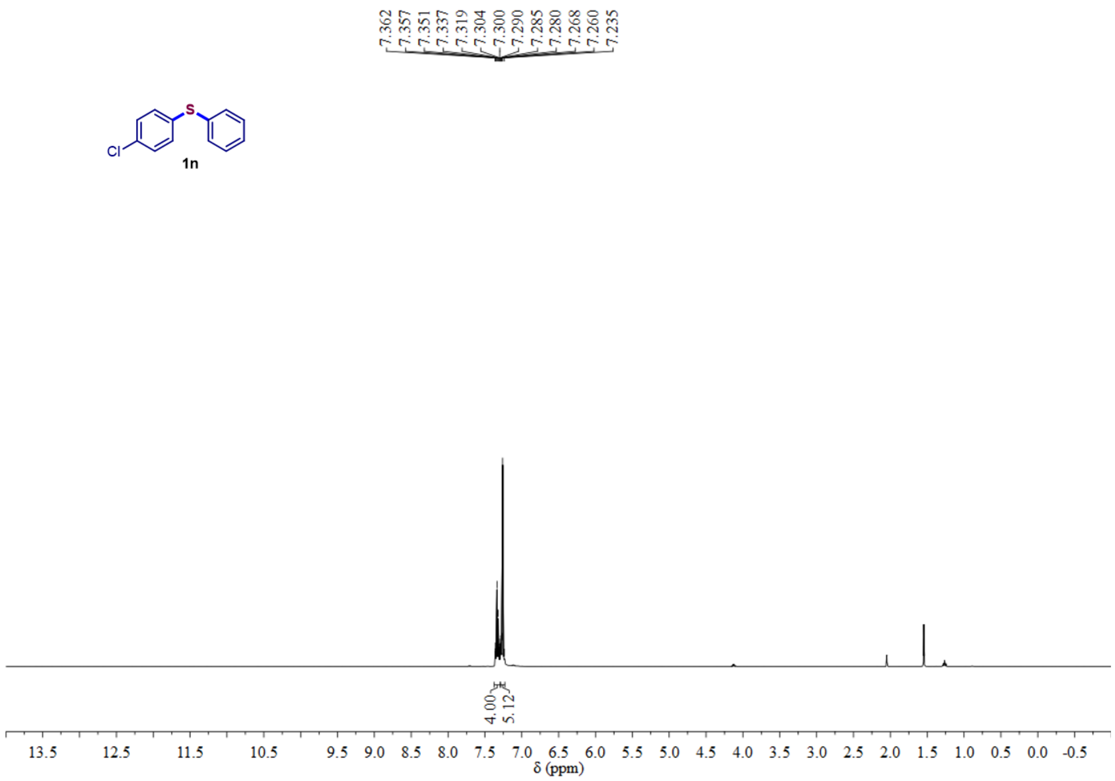

Figure S89. ^{1}H NMR spectra of **1n**

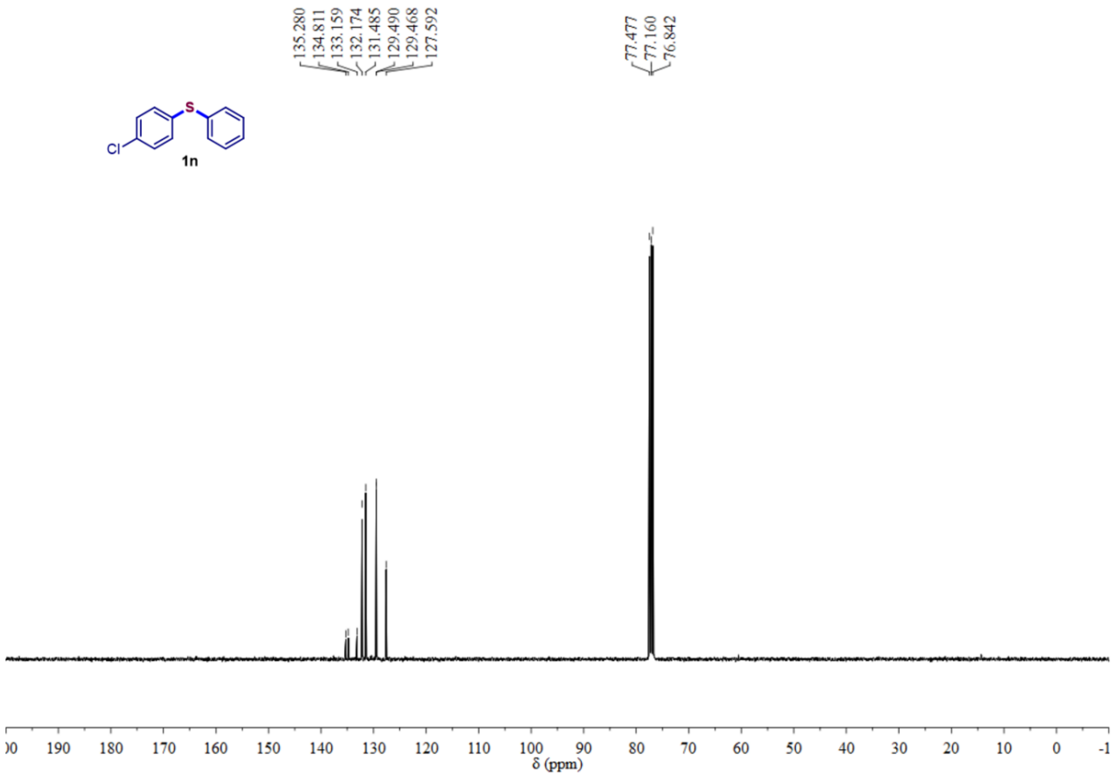

Figure S90. ^{13}C NMR spectra of **1n**

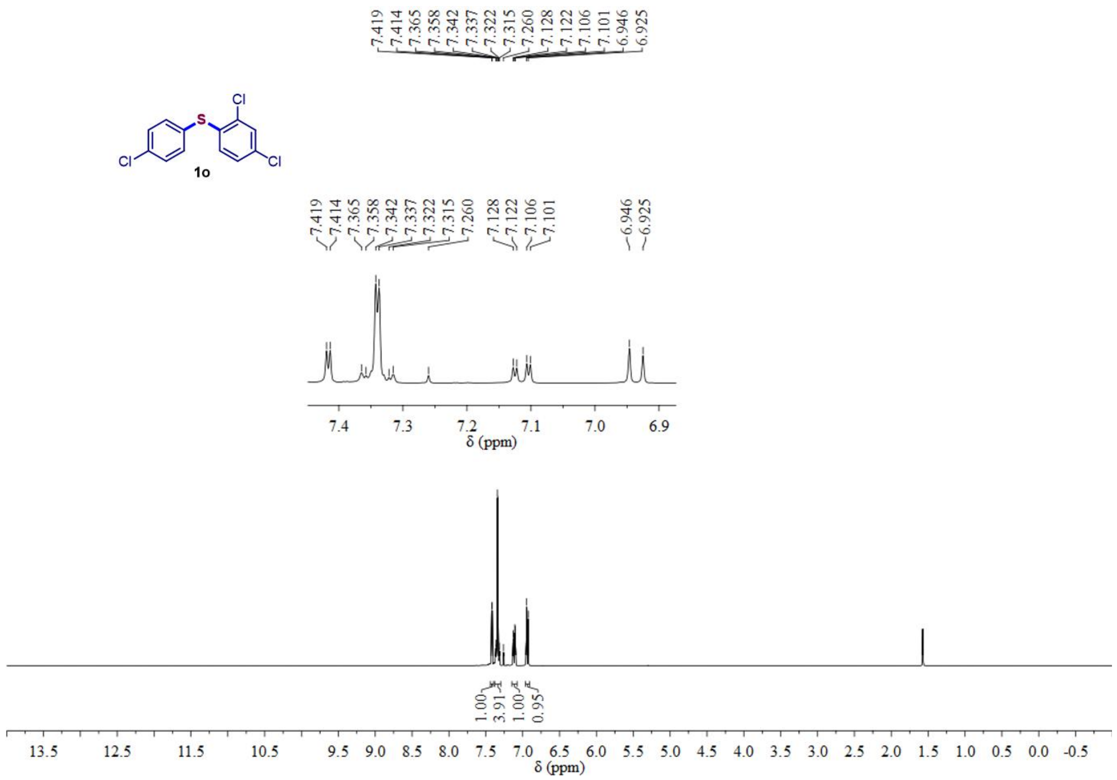

Figure S91. ^{1}H NMR spectra of **1o**

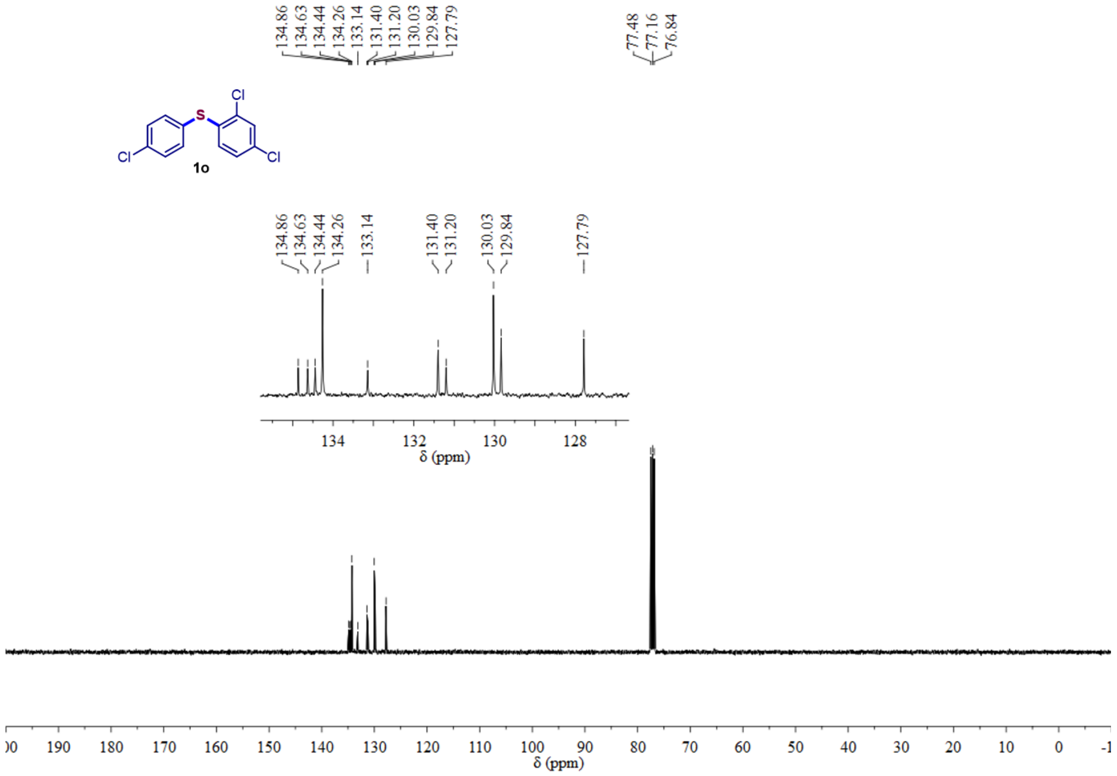

Figure S92. ^{13}C NMR spectra of **1o**

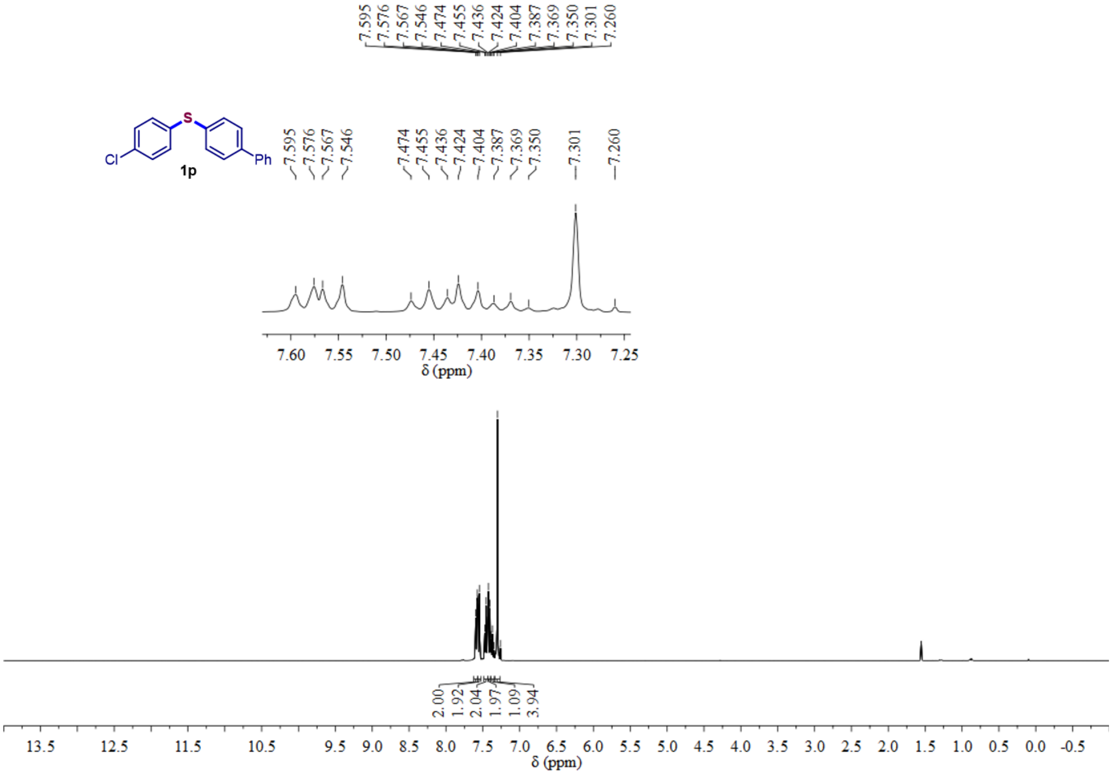

Figure S93. ^{1}H NMR spectra of **1p**

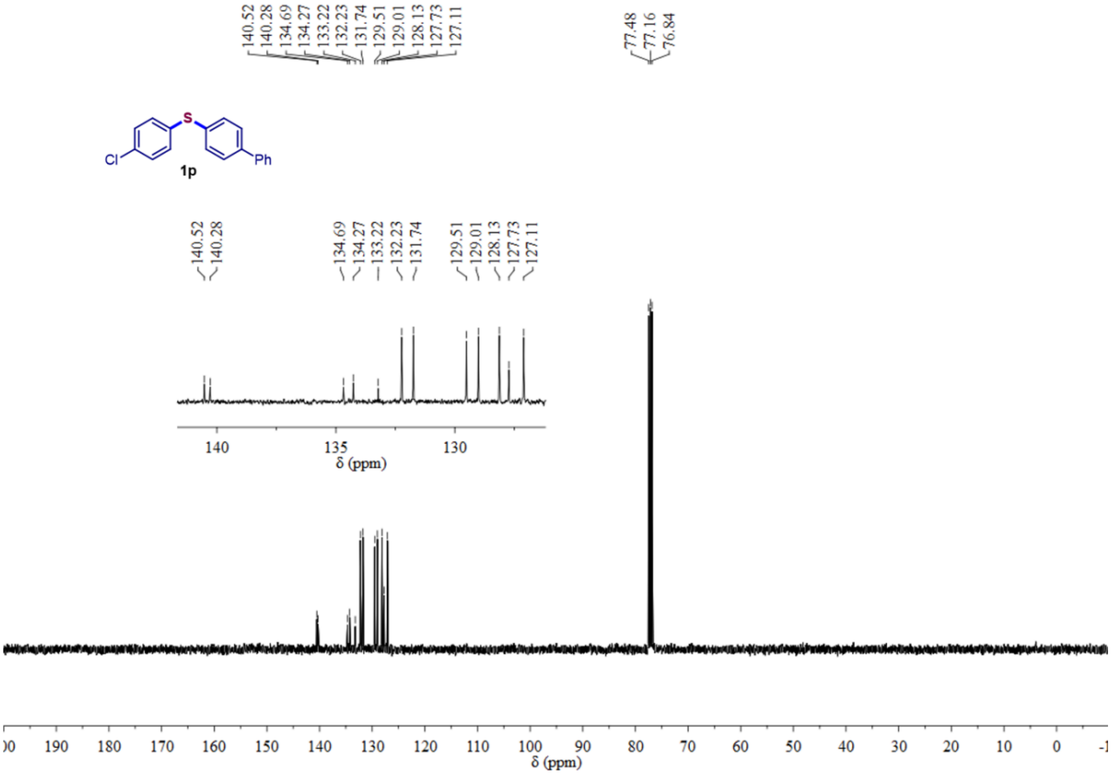

Figure S94. ^{13}C NMR spectra of **1p**

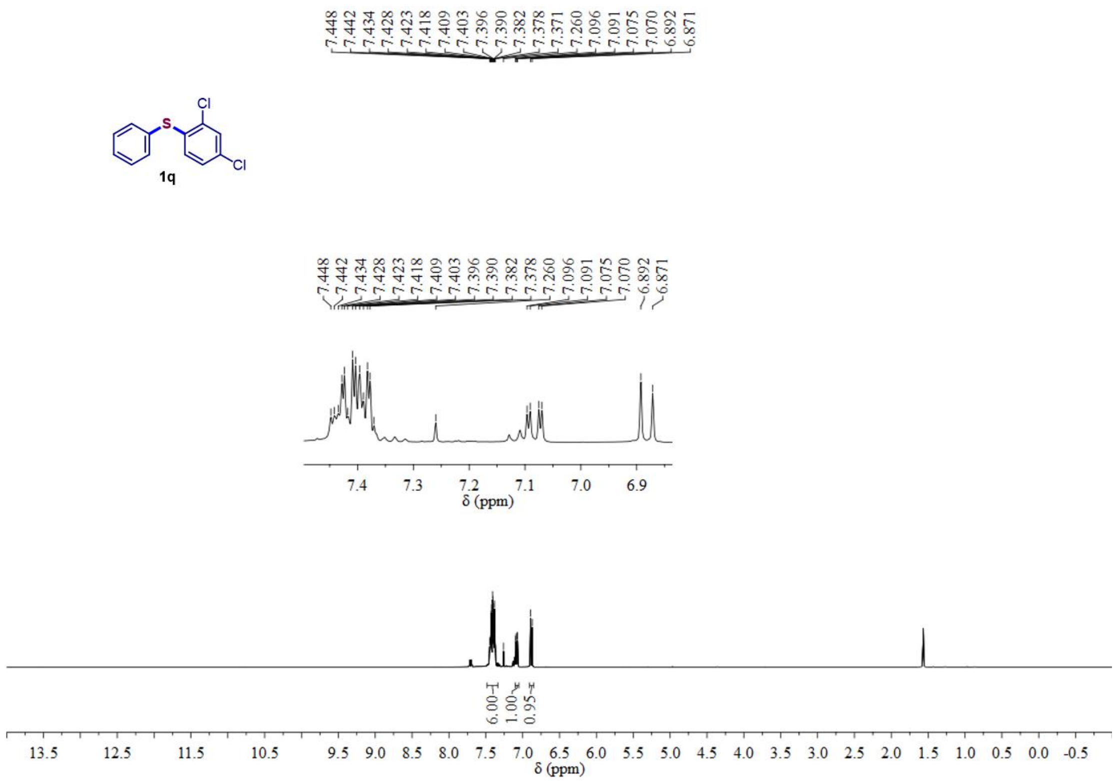

Figure S95. ^{1}H NMR spectra of **1q**

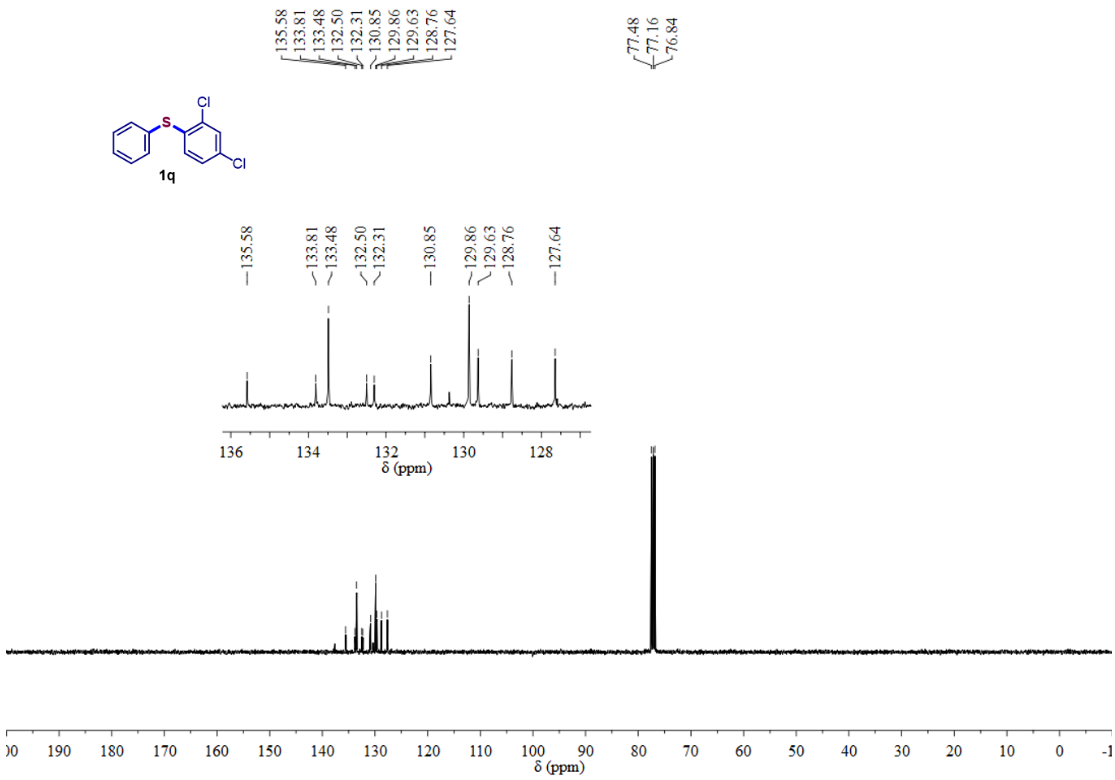

Figure S96. ^{13}C NMR spectra of **1q**

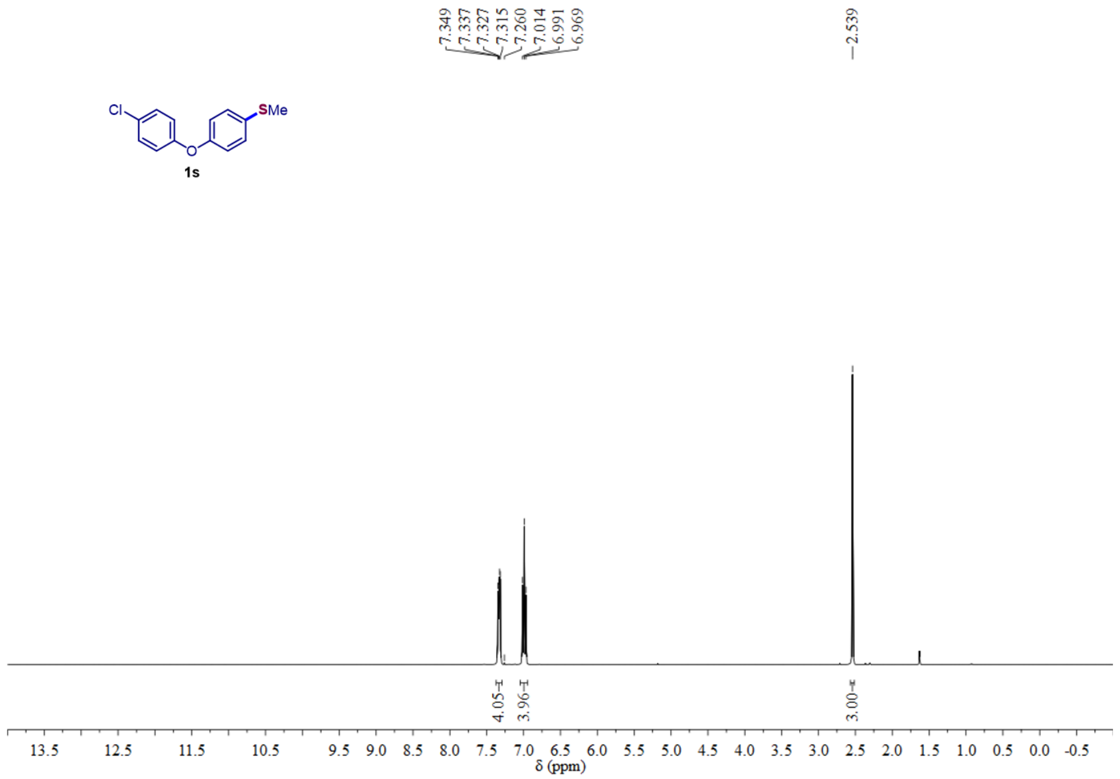

Figure S97. ^{1}H NMR spectra of **1s**

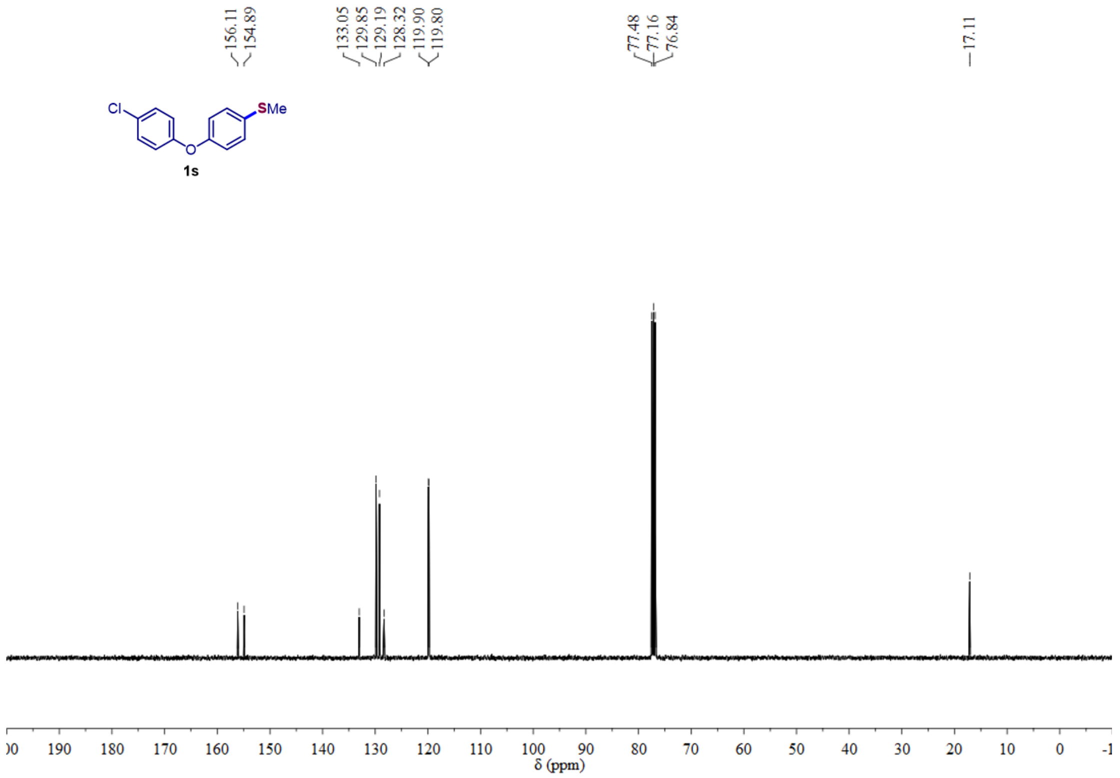

Figure S98. ^{13}C NMR spectra of **1s**

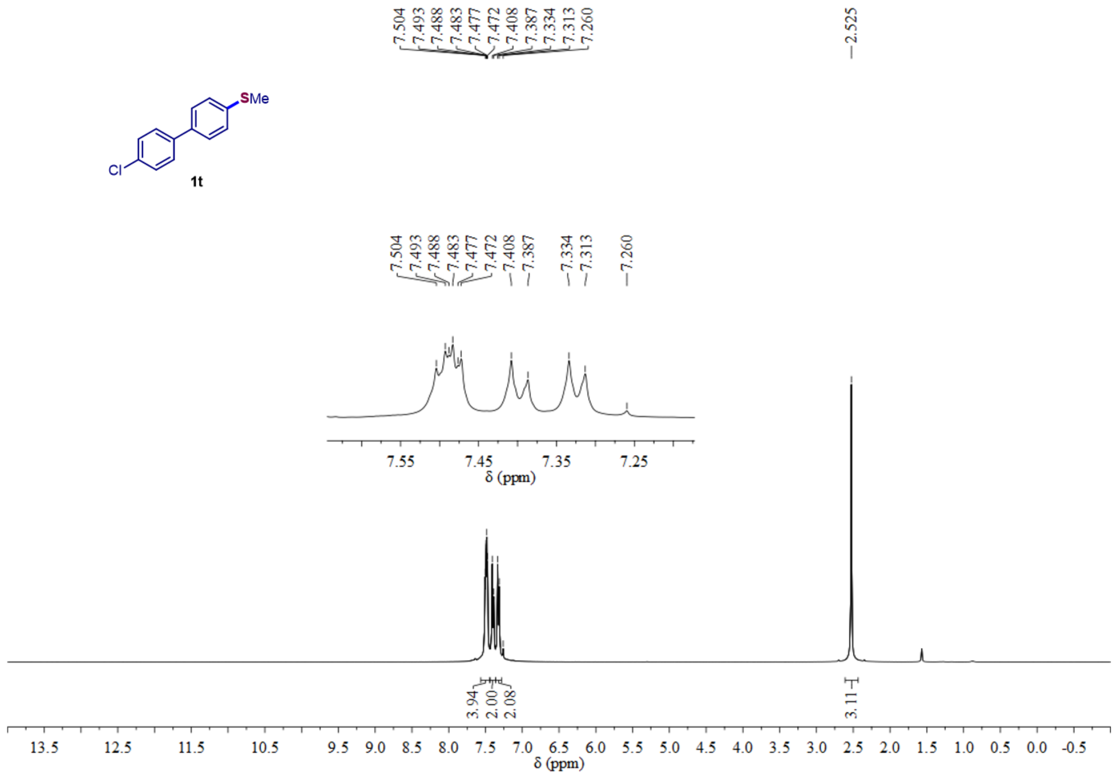

Figure S99. ^{1}H NMR spectra of **1t**

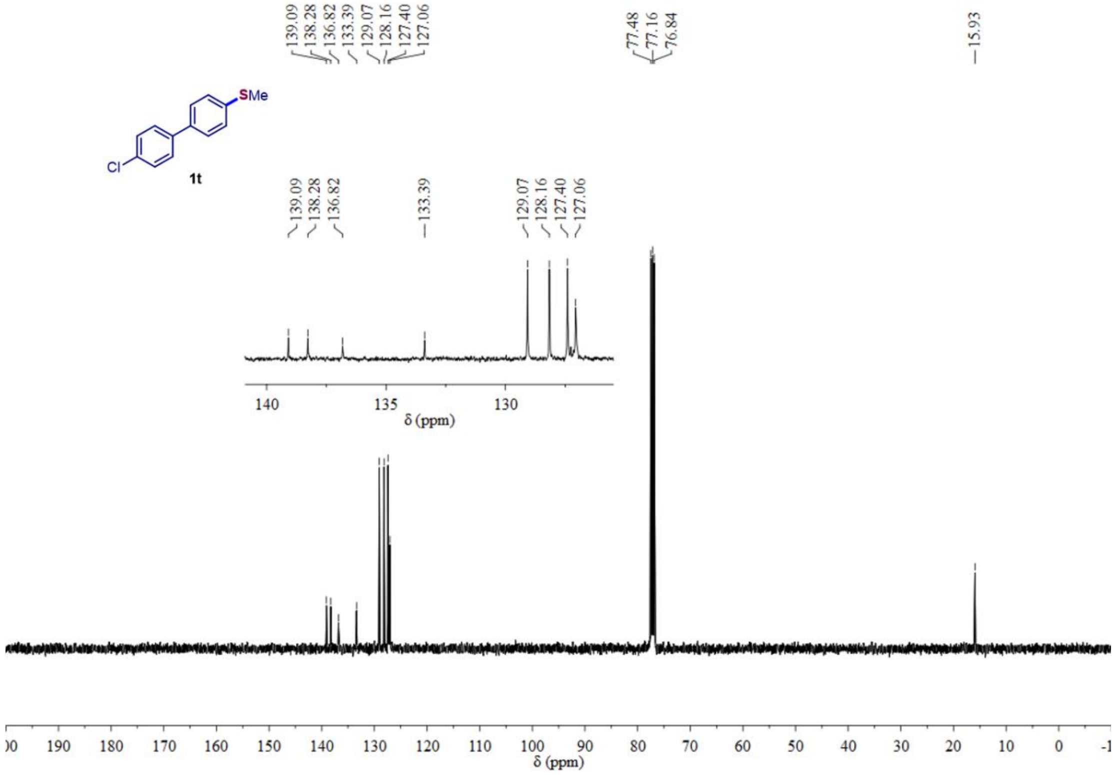

Figure S100. ^{13}C NMR spectra of **1t**

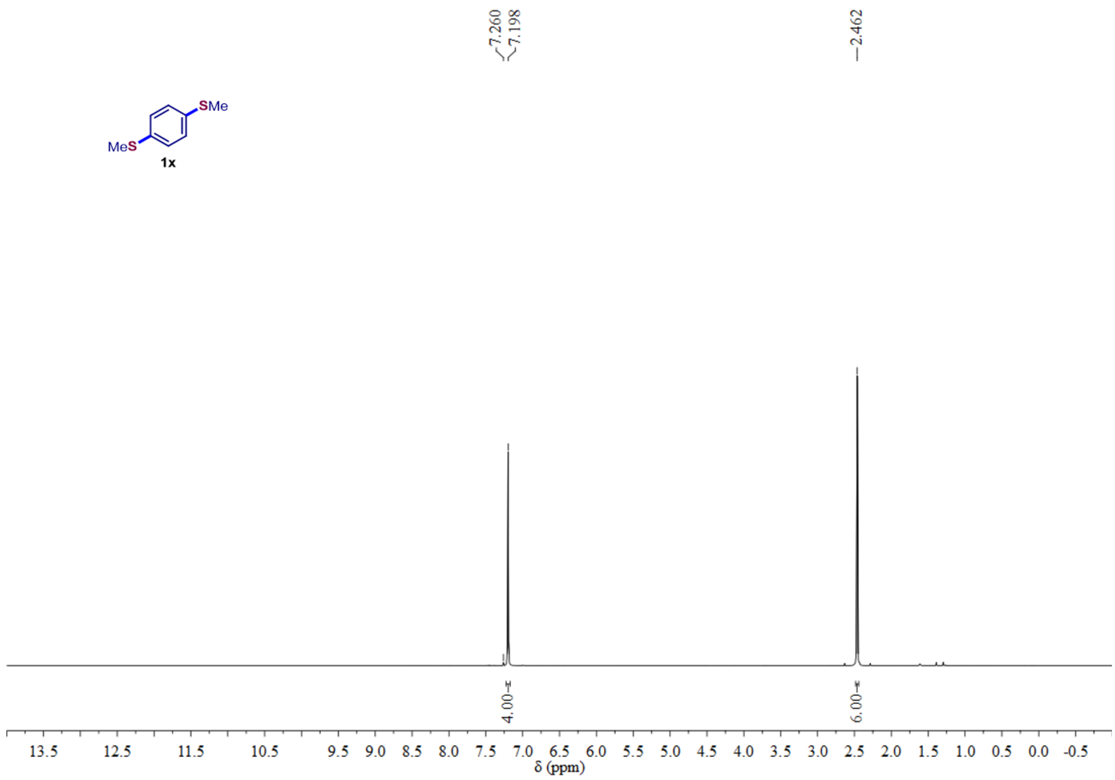

Figure S101. ^{1}H NMR spectra of **1x**

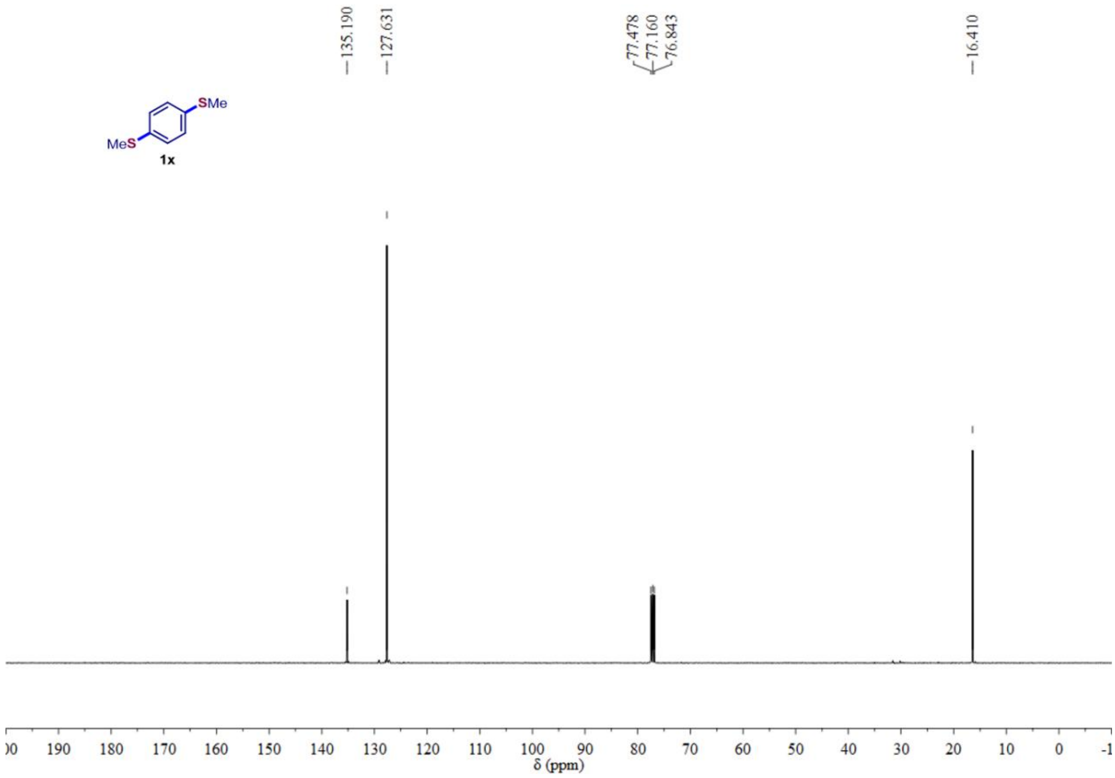

Figure S102. ^{13}C NMR spectra of **1x**

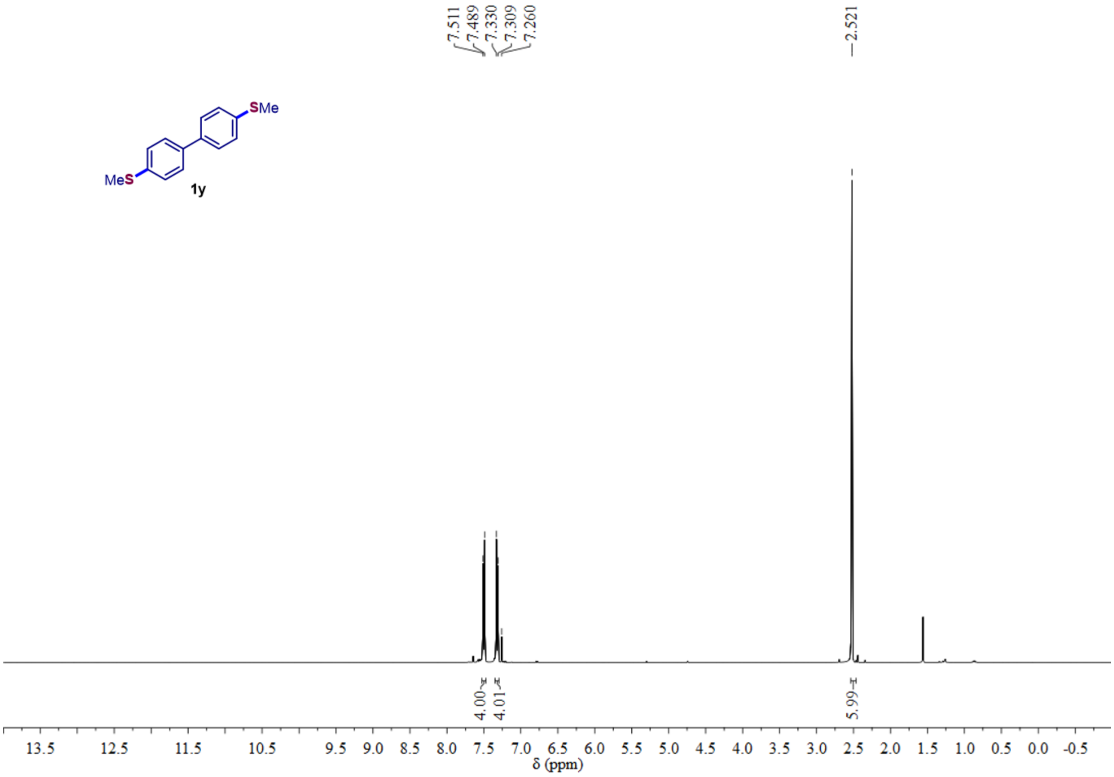

Figure S103. ^{1}H NMR spectra of **1y**

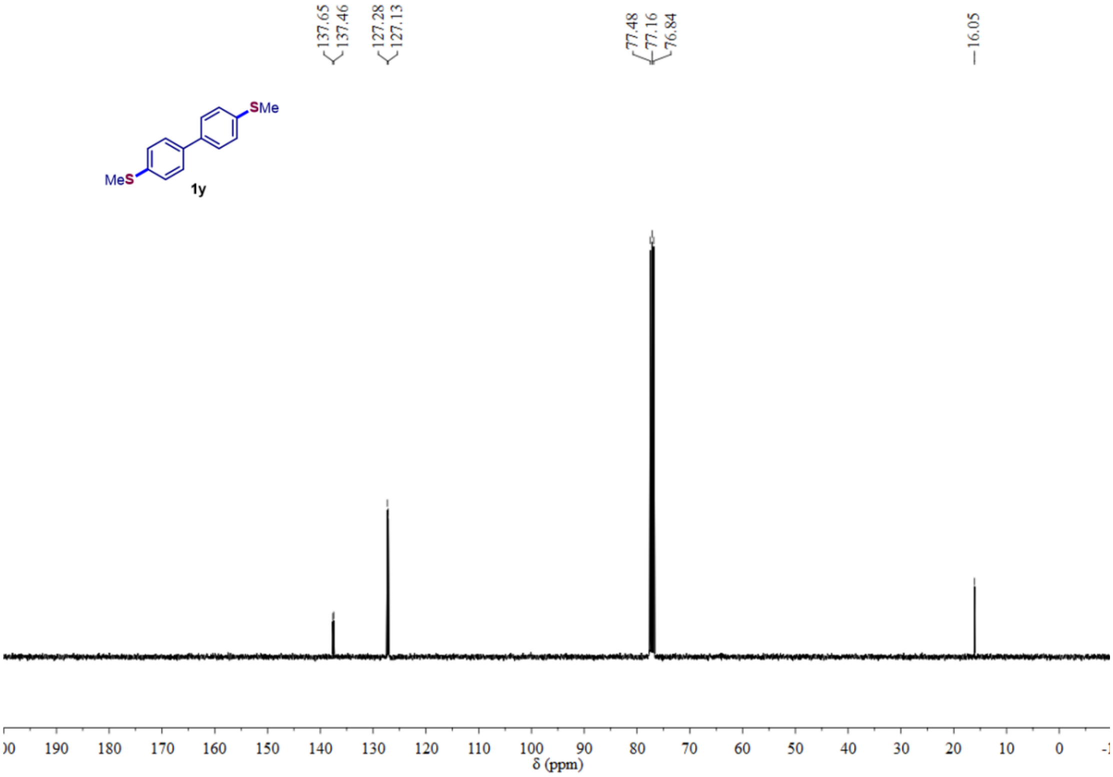

Figure S104. ^{13}C NMR spectra of **1y**

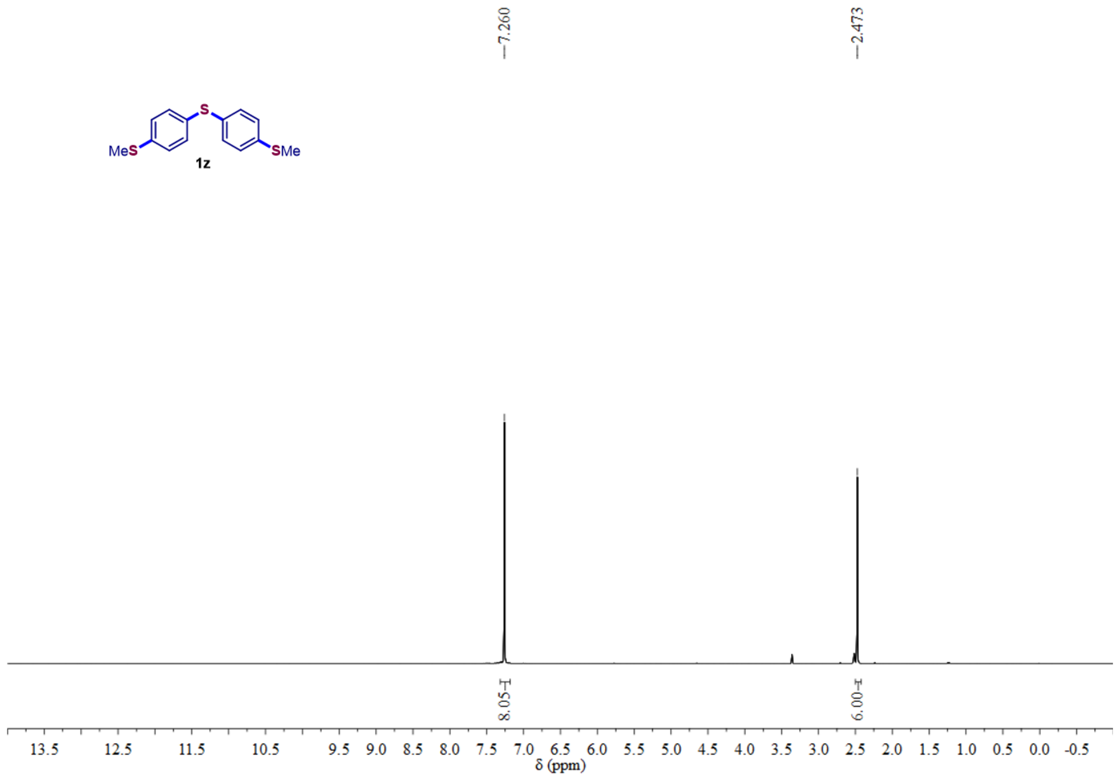

Figure S105. ^{1}H NMR spectra of **1z**

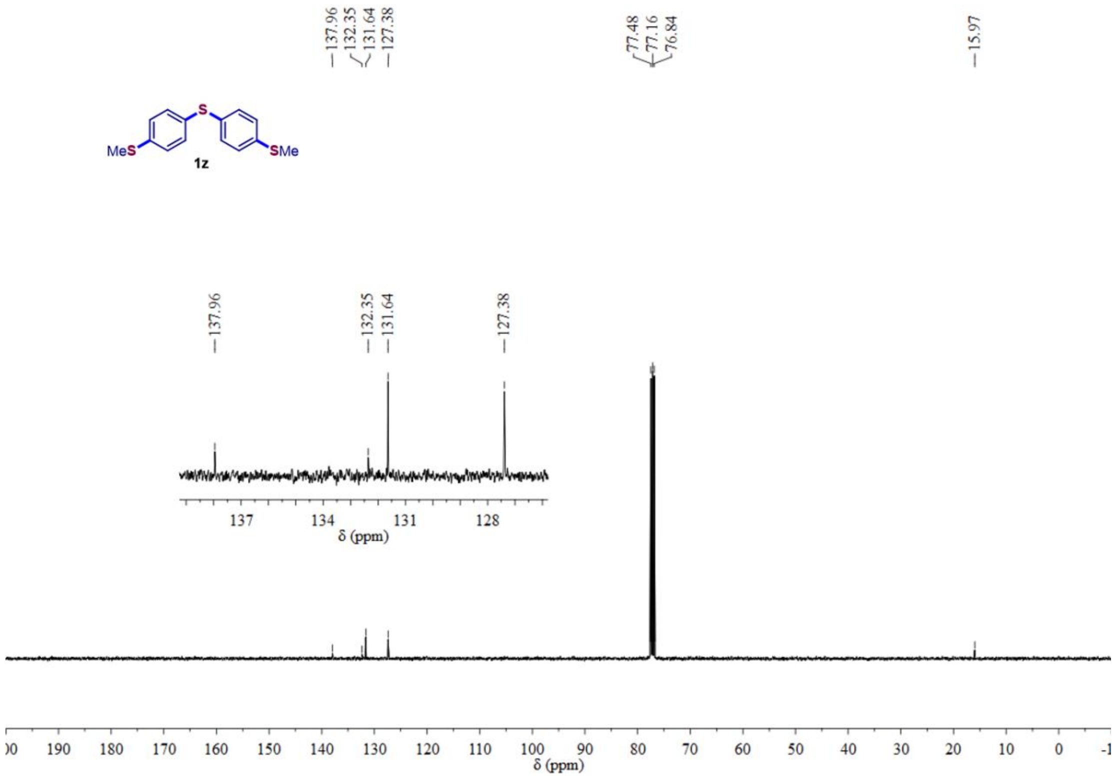

Figure S106. ^{13}C NMR spectra of **1z**

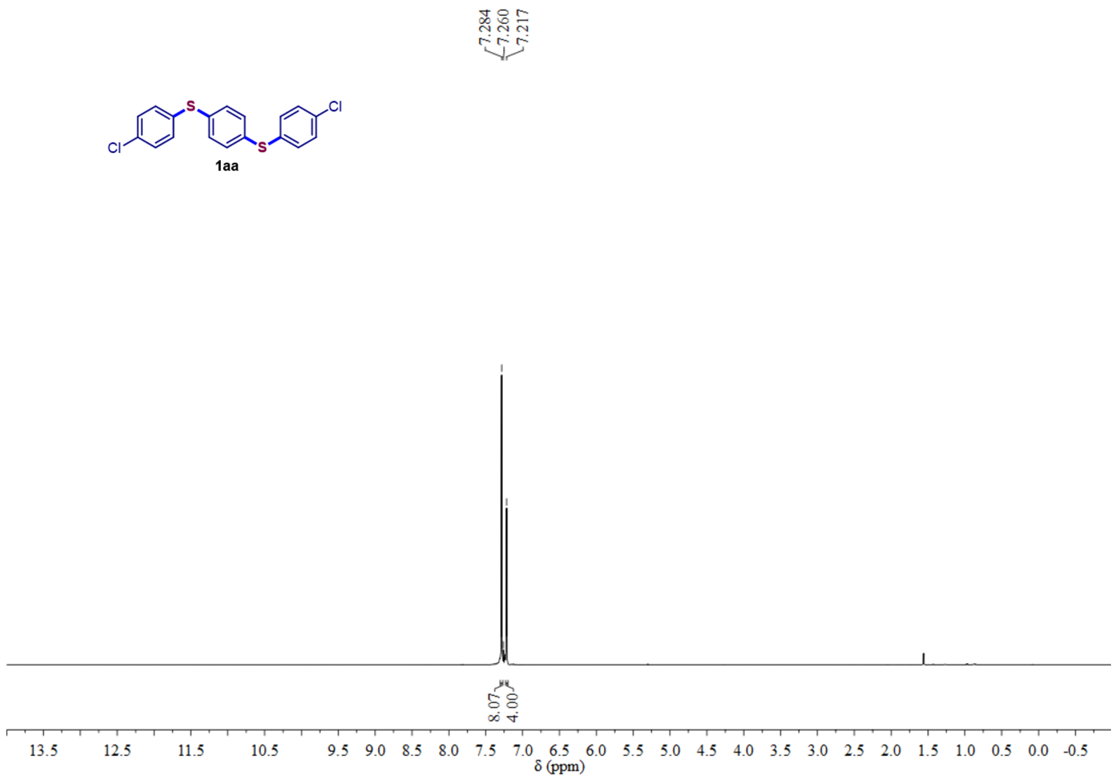

Figure S107. ^{1}H NMR spectra of **1aa**

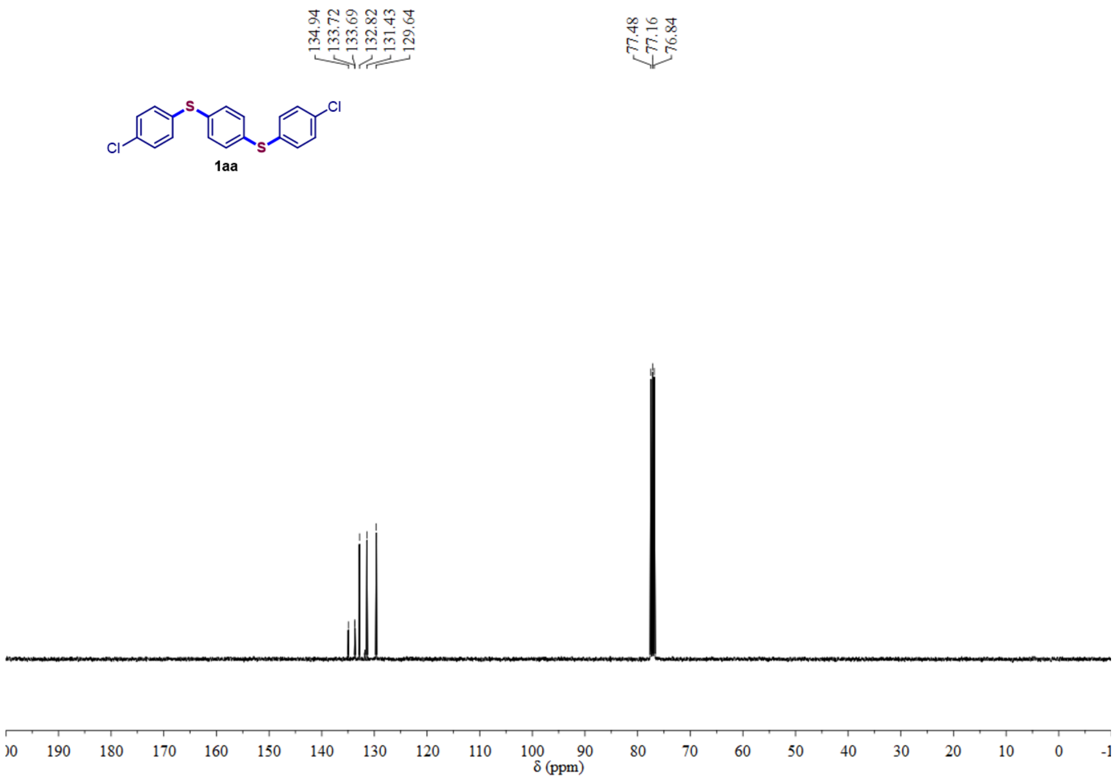

Figure S108. ^{13}C NMR spectra of **1aa**

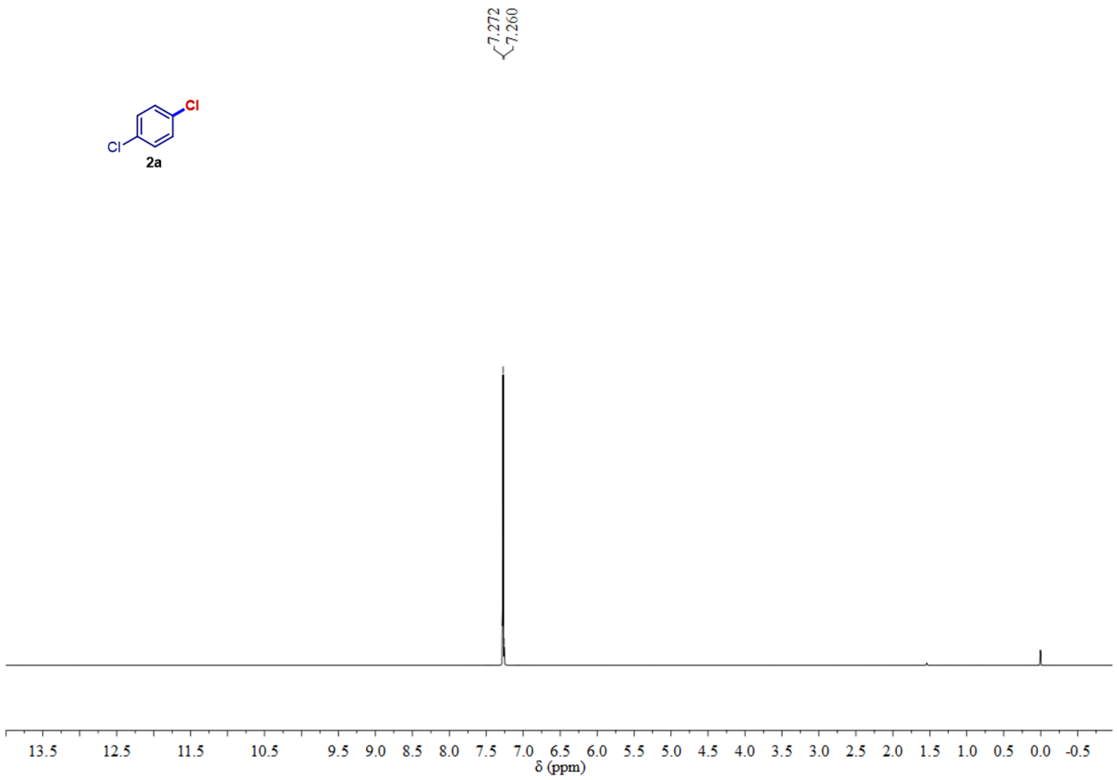

Figure S109. ^{1}H NMR spectra of **2a**

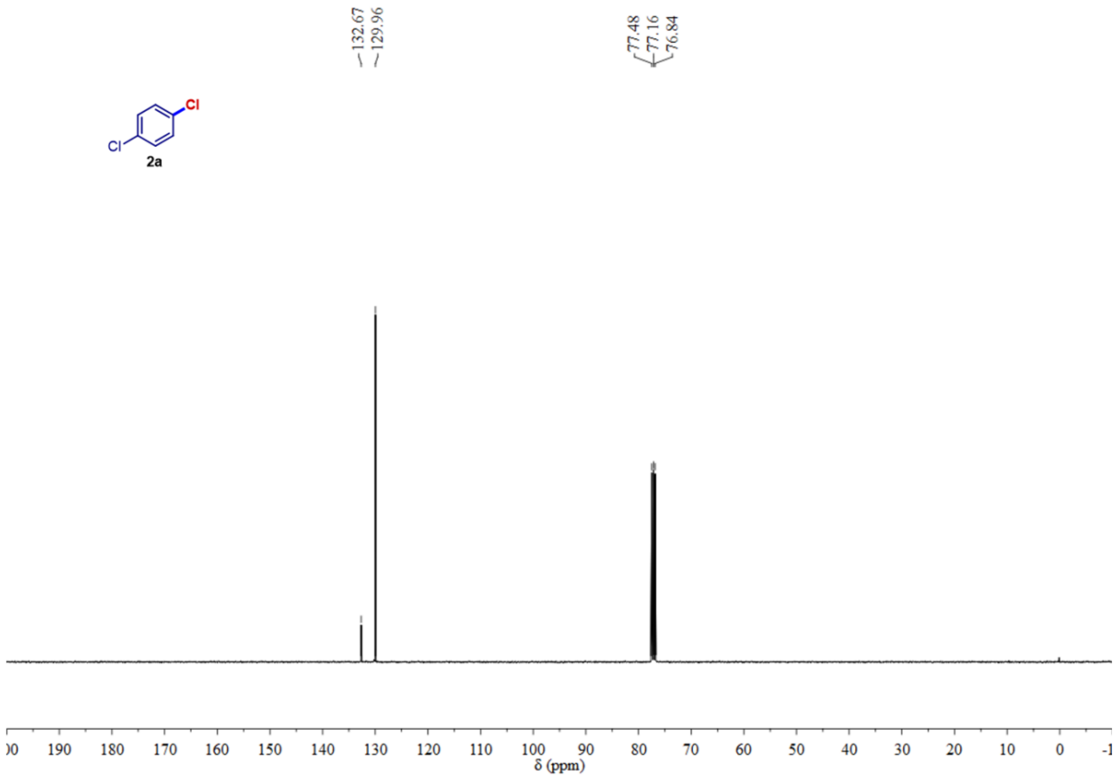

Figure S110. ^{13}C NMR spectra of **2a**

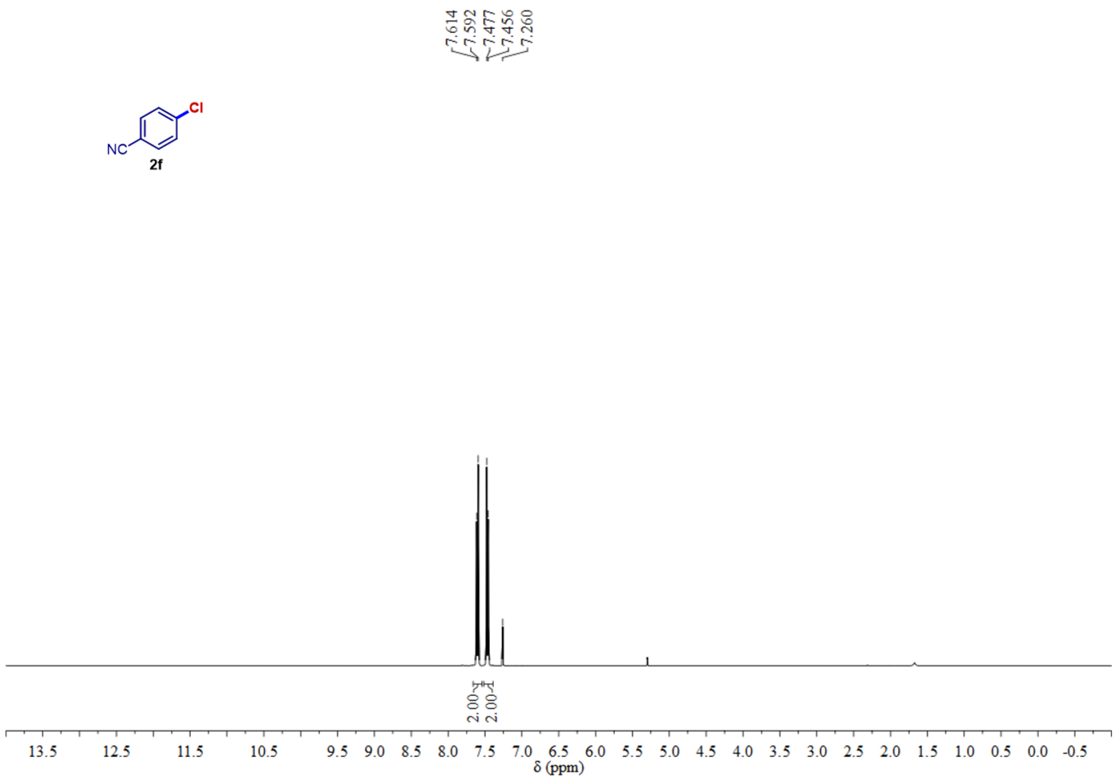

Figure S111. ^{1}H NMR spectra of **2f**

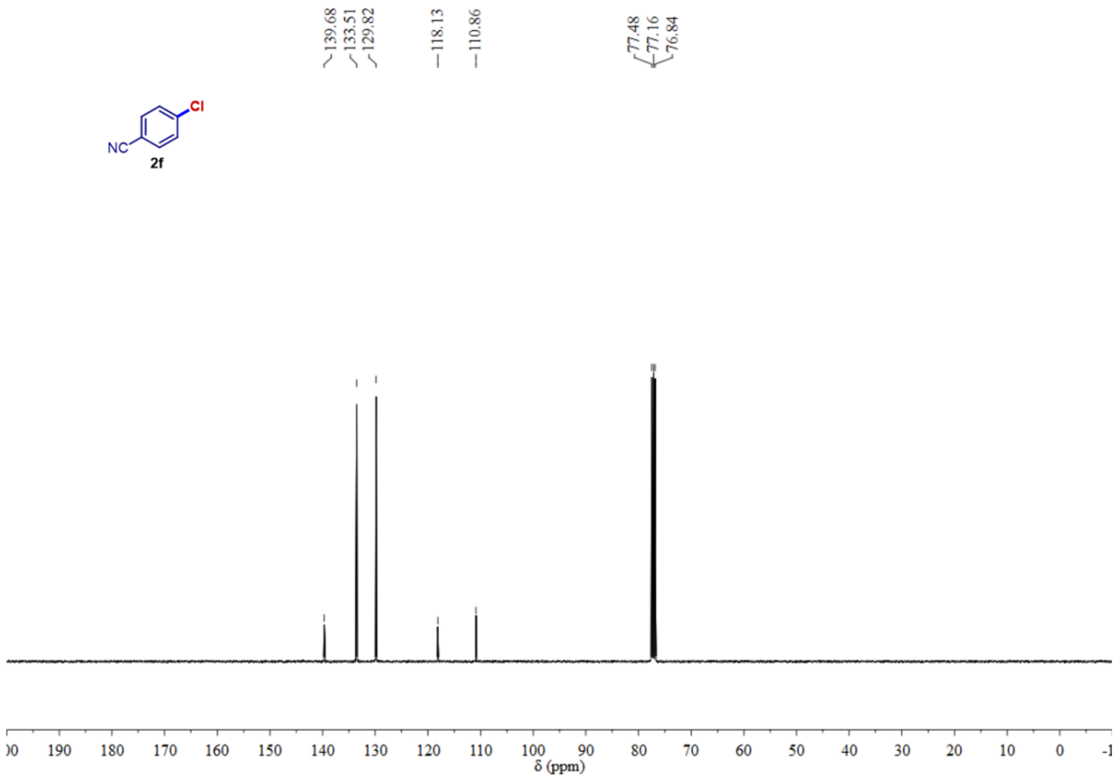

Figure S112. ^{13}C NMR spectra of **2f**

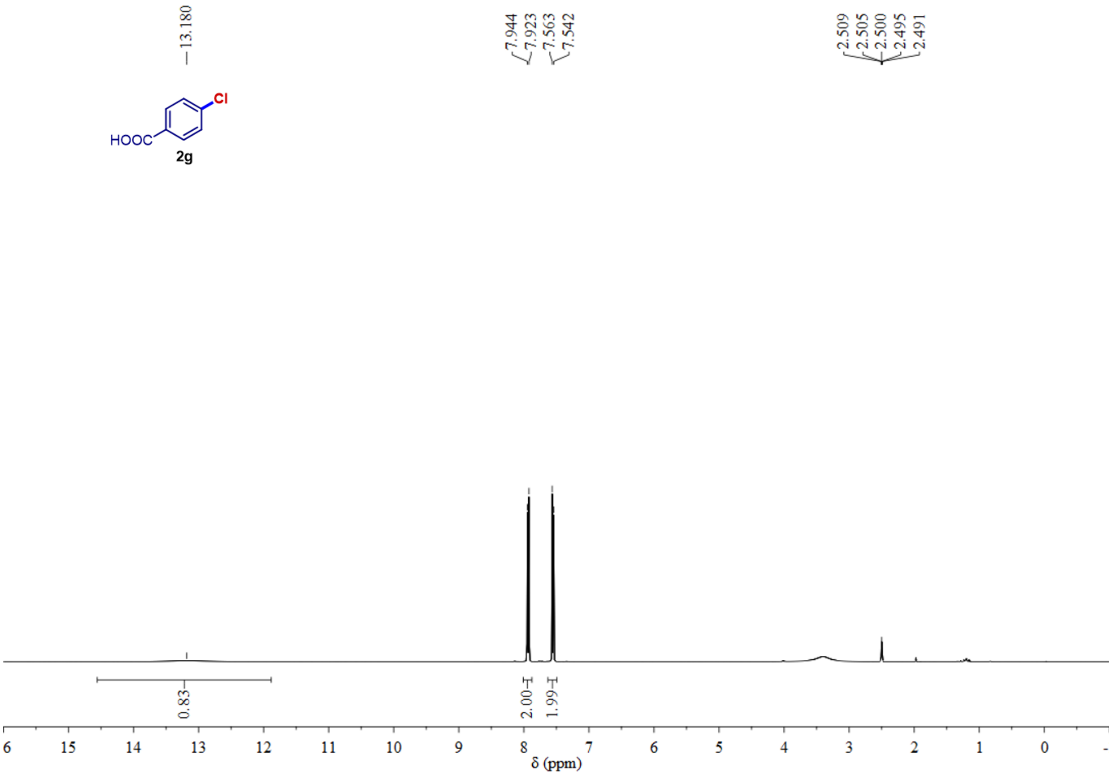

Figure S113. ^{1}H NMR spectra of **2g**

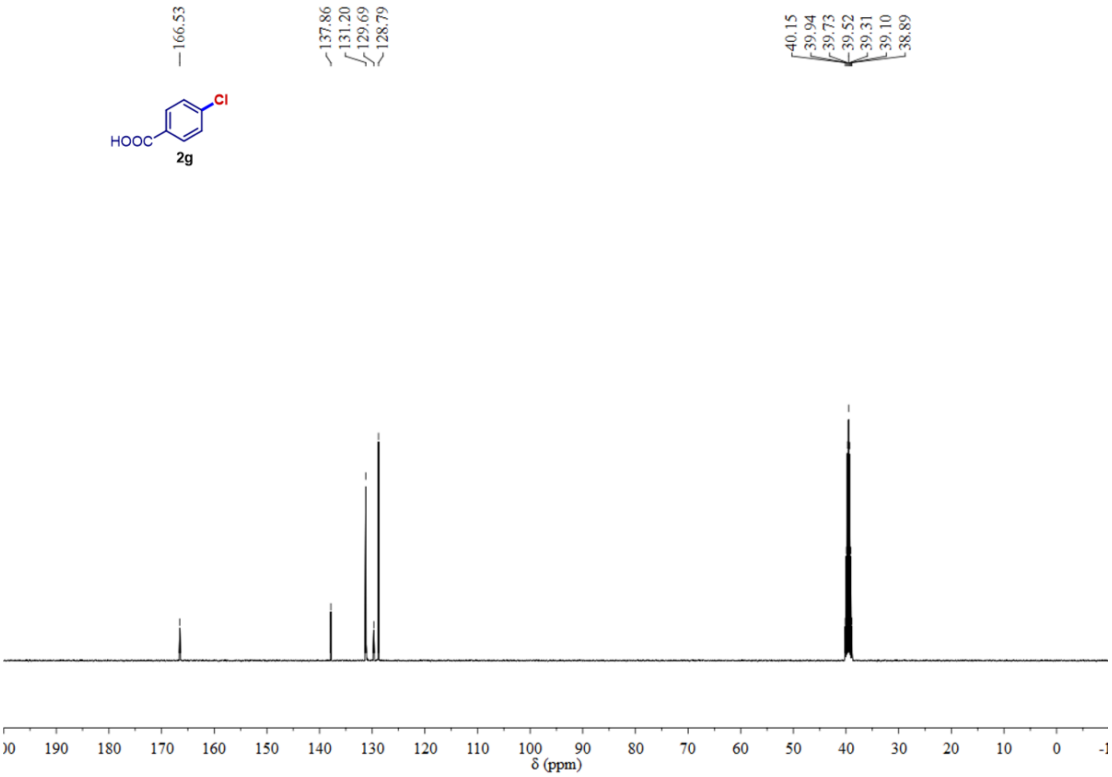

Figure S114. ^{13}C NMR spectra of **2g**

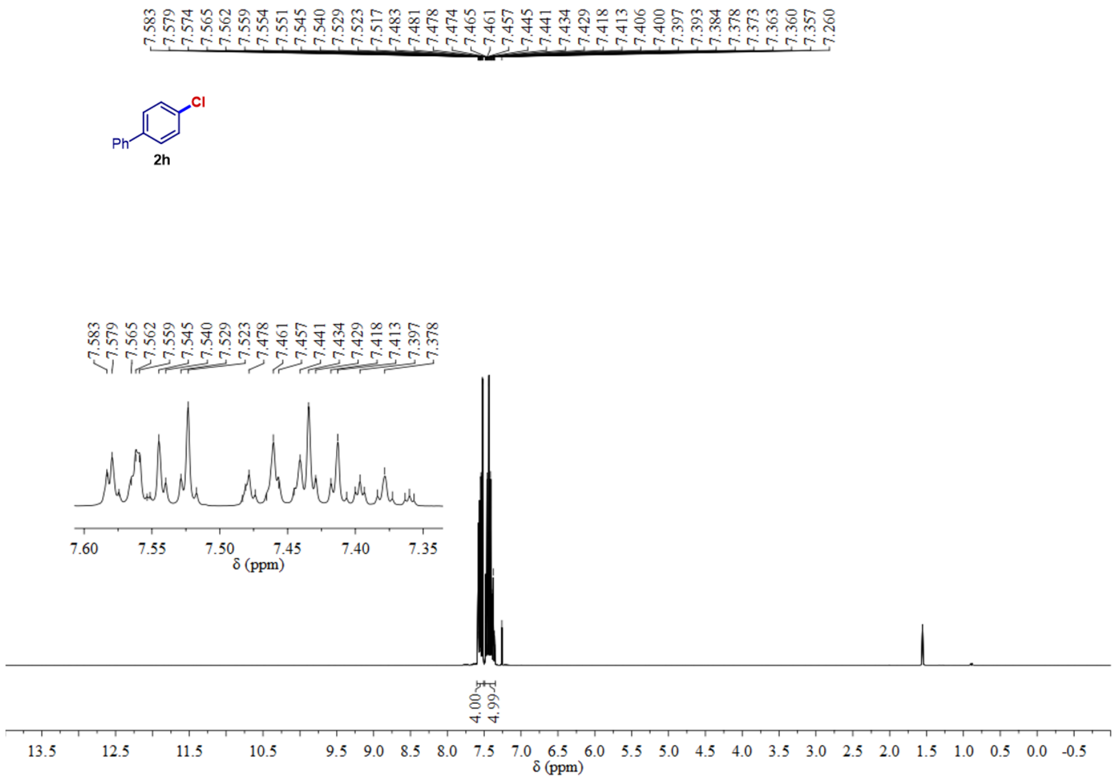

Figure S115. ^{1}H NMR spectra of **2h**

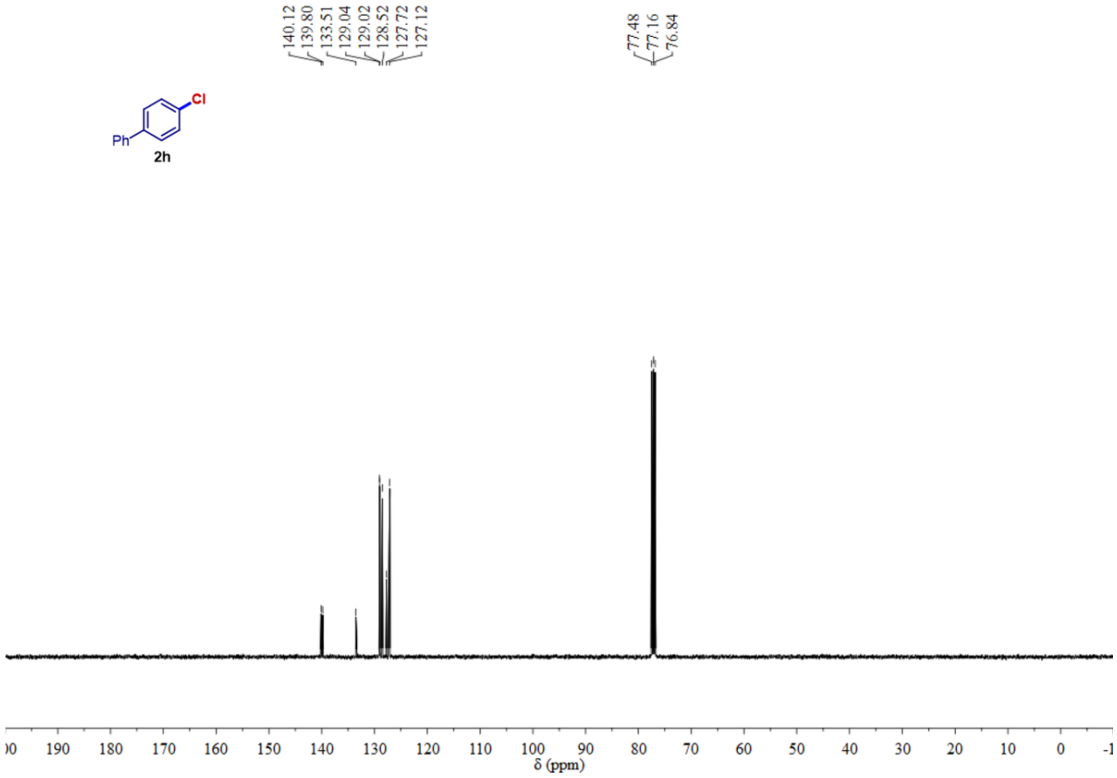

Figure S116. ^{13}C NMR spectra of **2h**

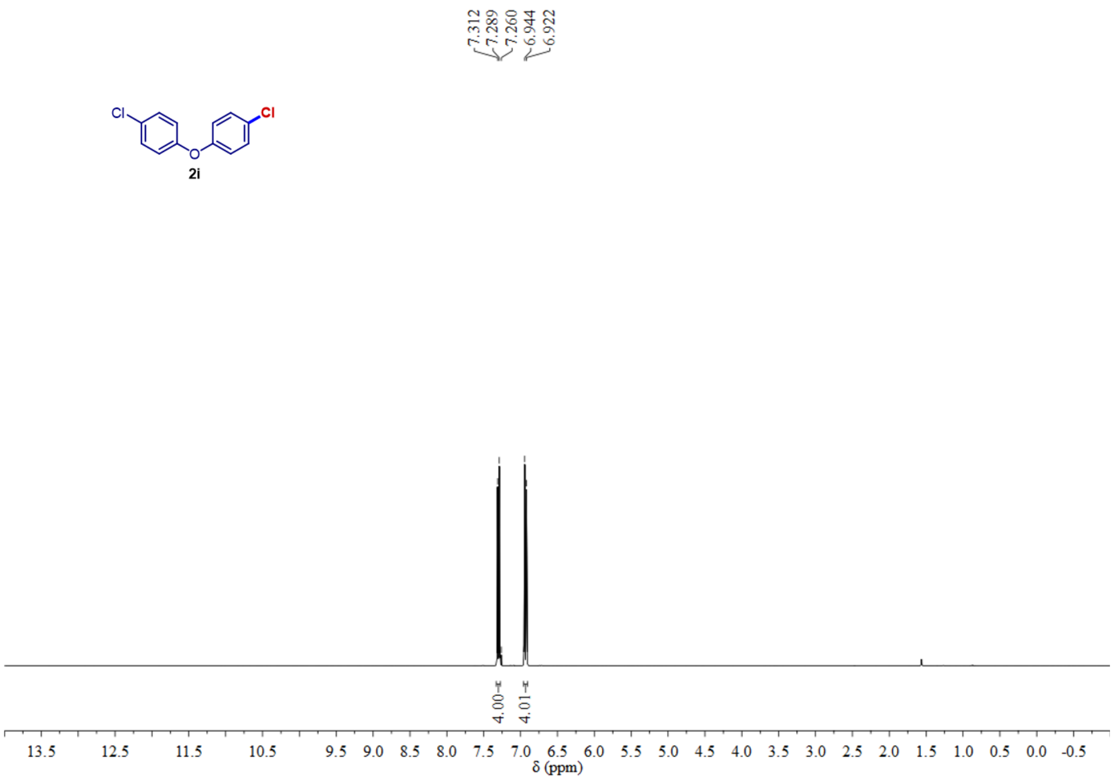

Figure S117. ^{1}H NMR spectra of **2i**

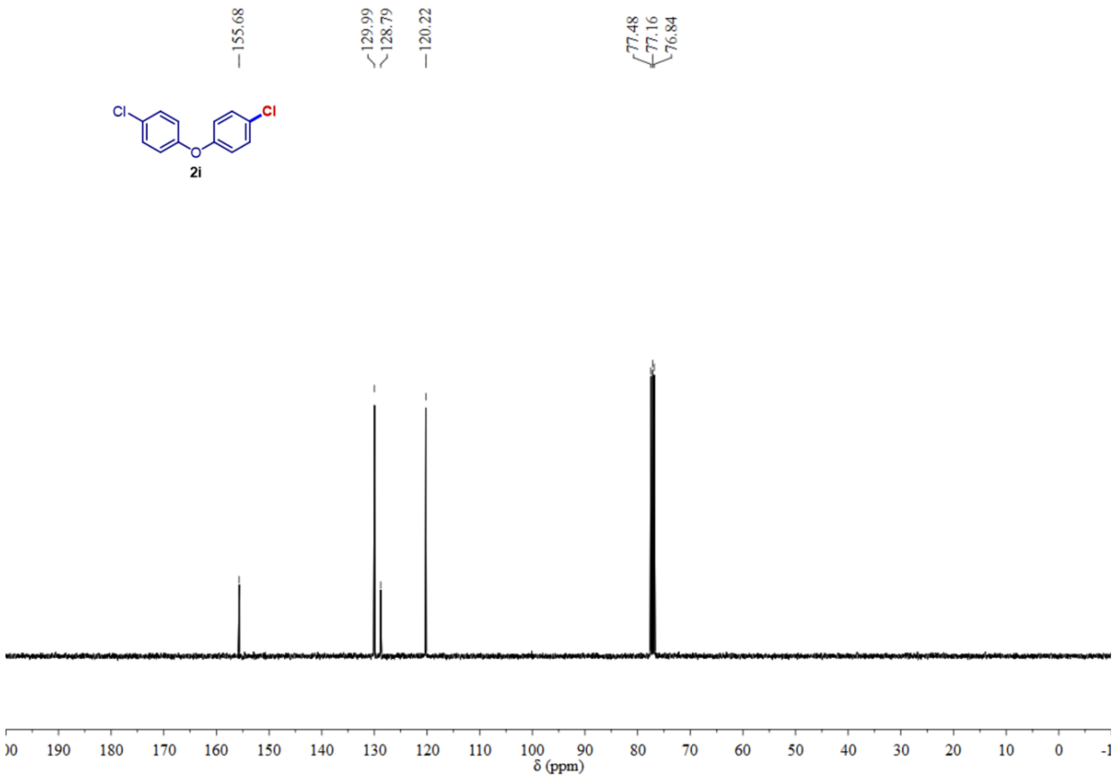

Figure S118. ^{13}C NMR spectra of **2i**

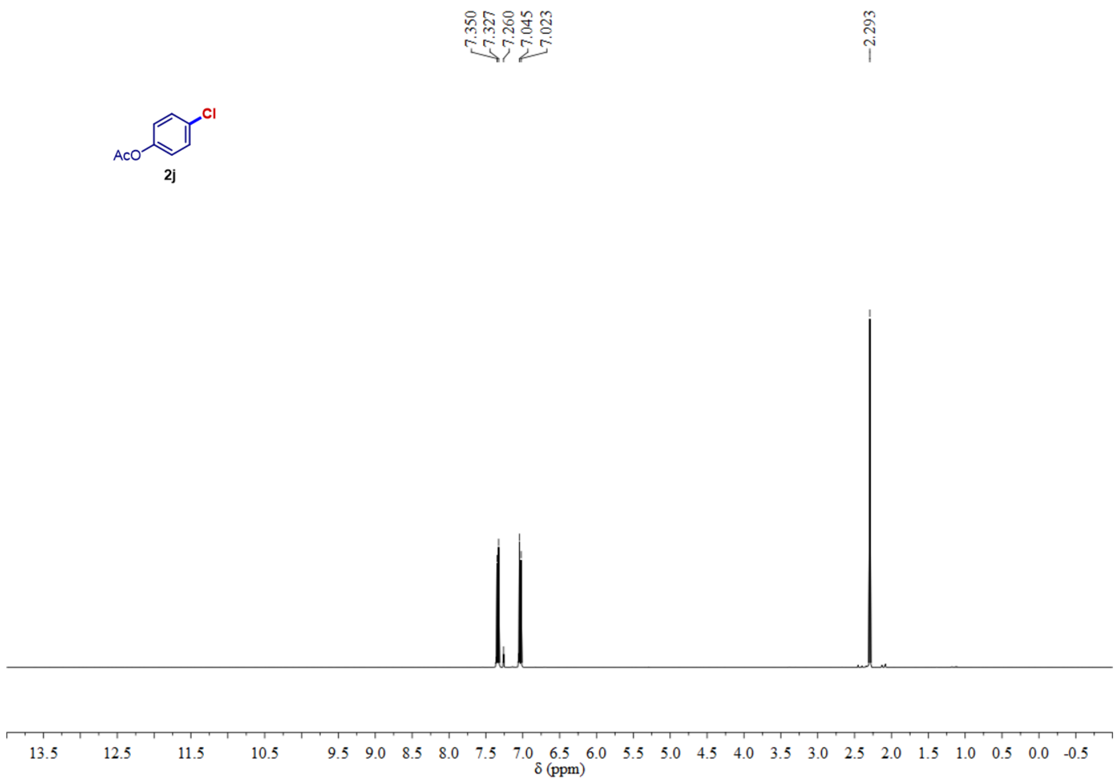

Figure S119. ^{1}H NMR spectra of **2j**

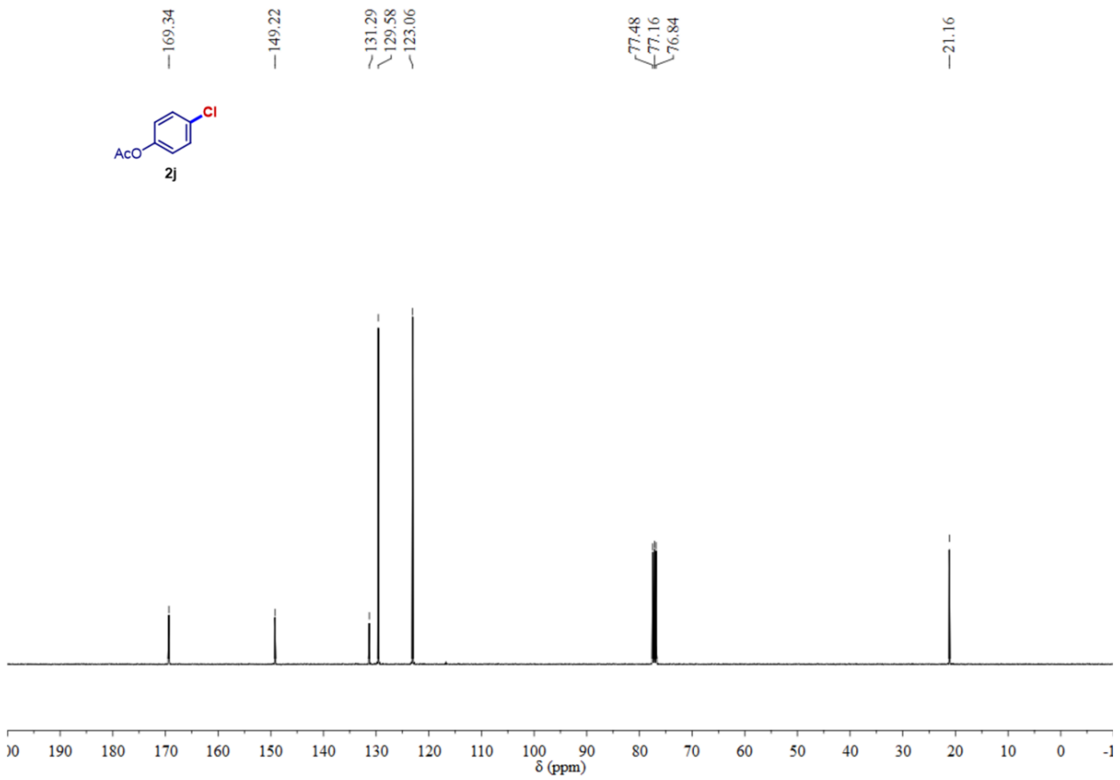

Figure S120. ^{13}C NMR spectra of **2j**

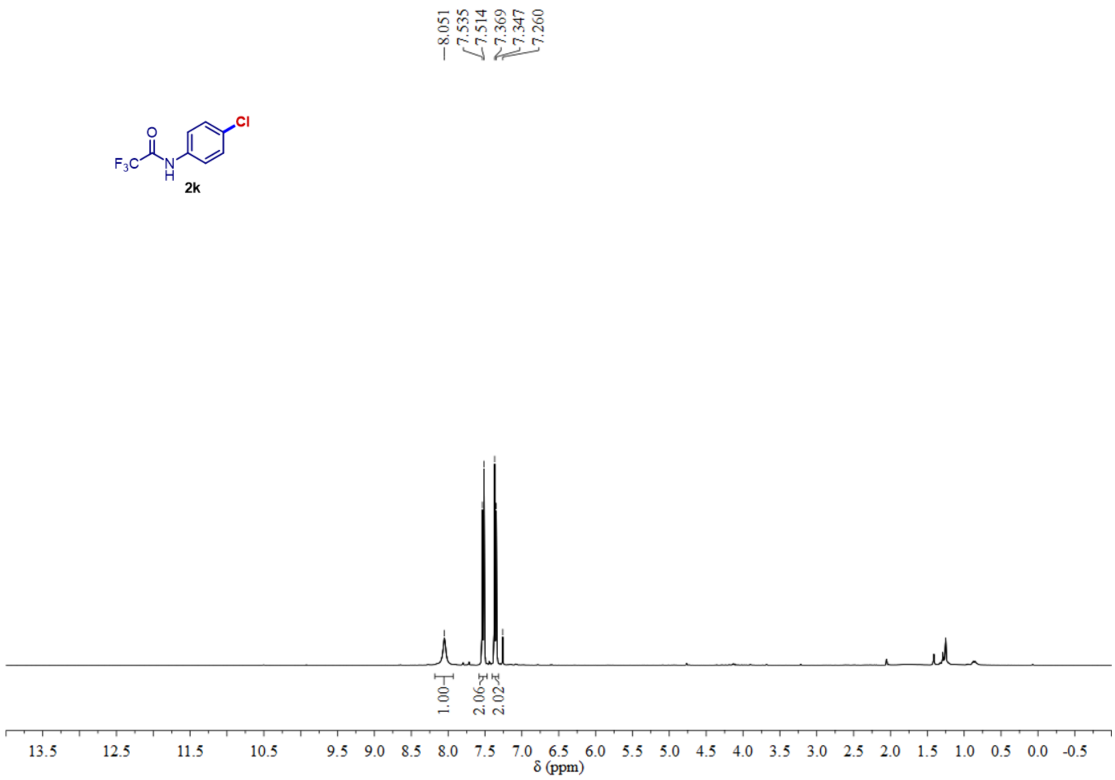

Figure S121. ^{1}H NMR spectra of **2k**

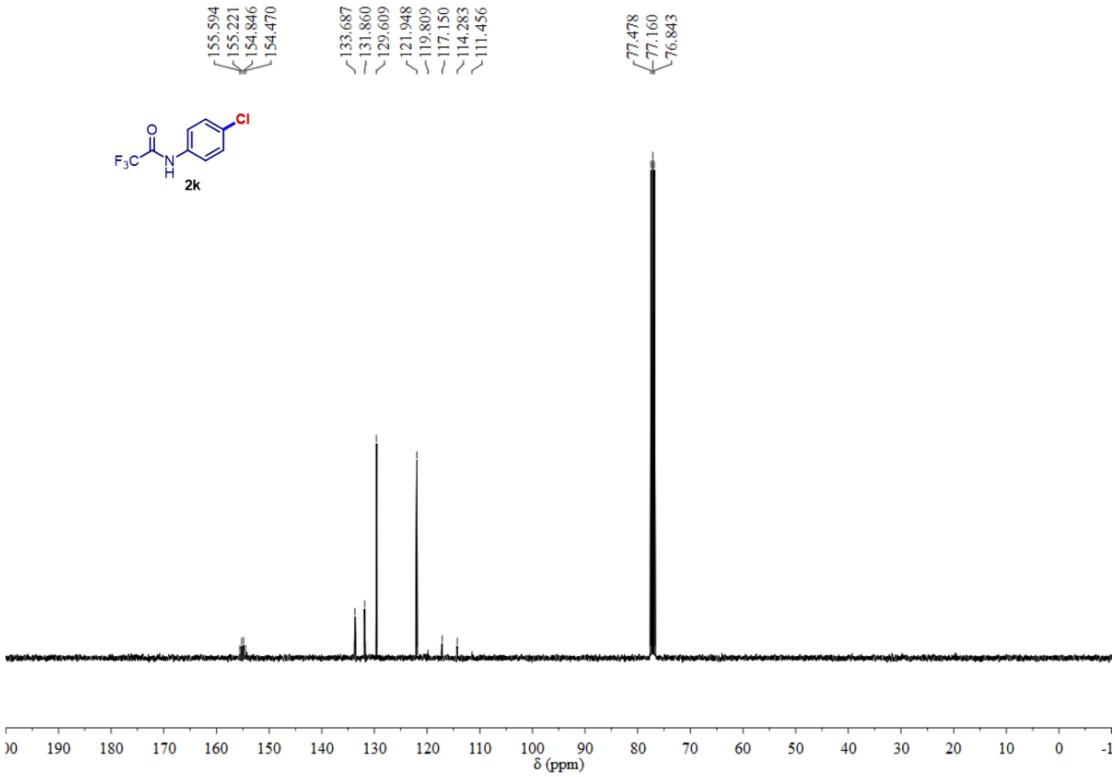

Figure S122. ^{13}C NMR spectra of **2k**

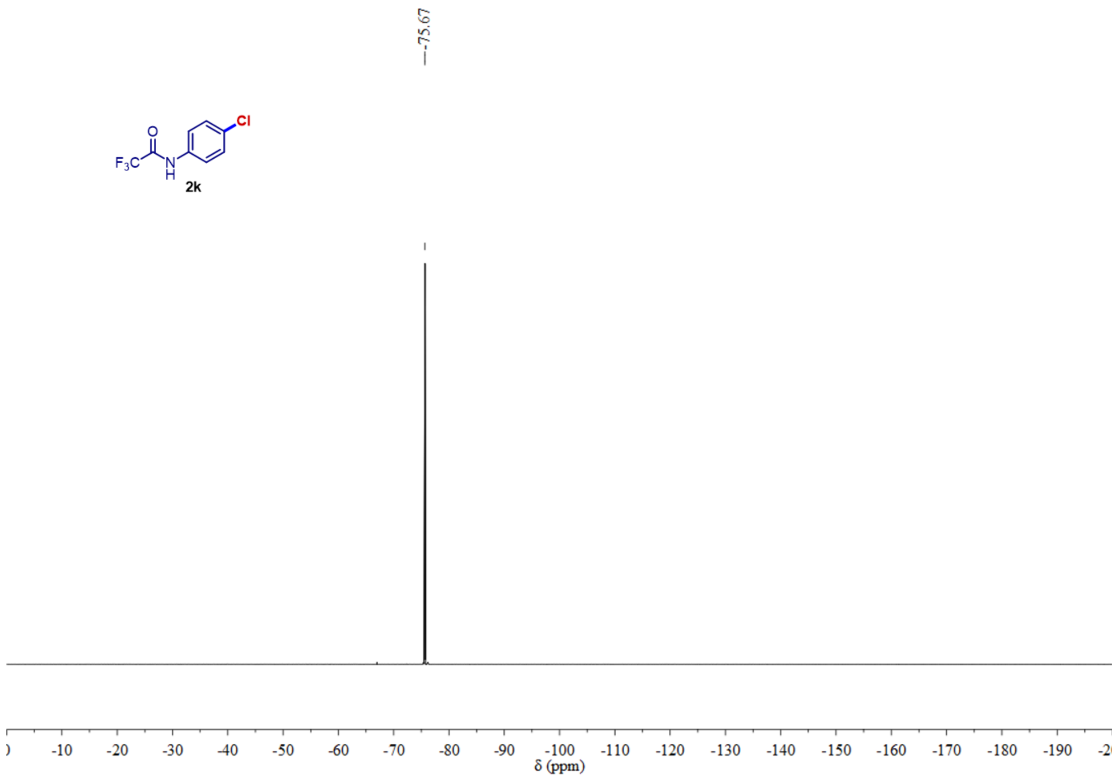

Figure S123. ^{19}F NMR spectra of **2k**

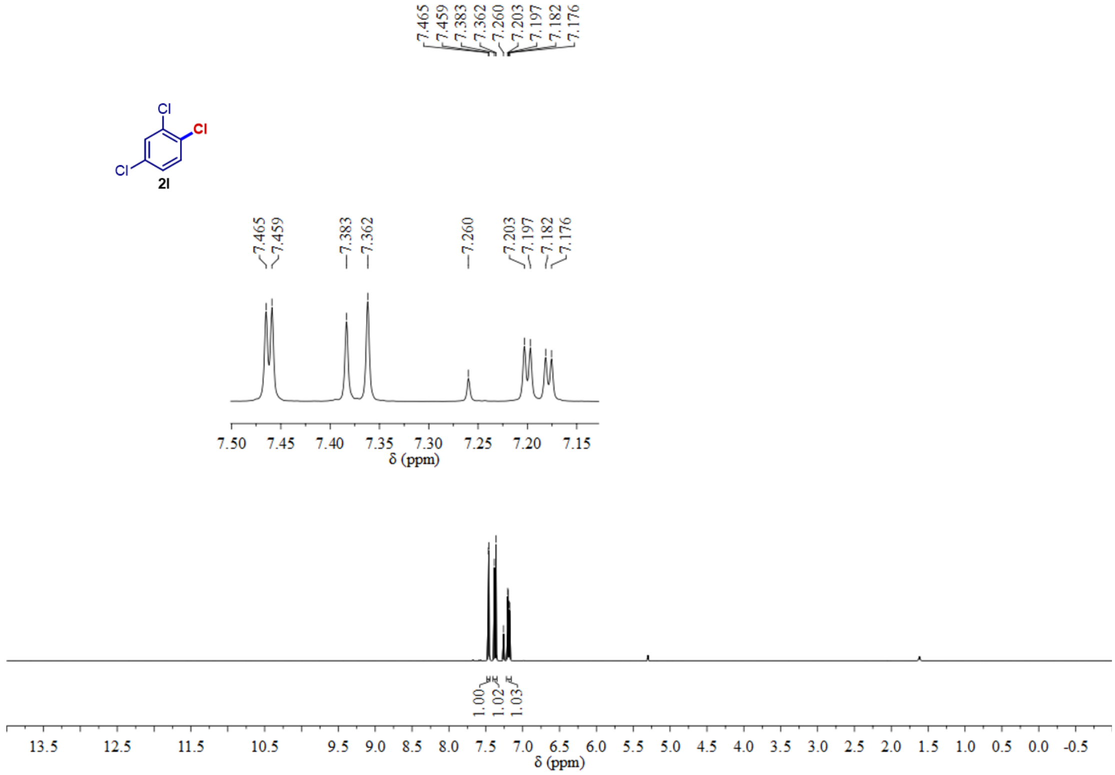

Figure S124. ^{1}H NMR spectra of **2l**

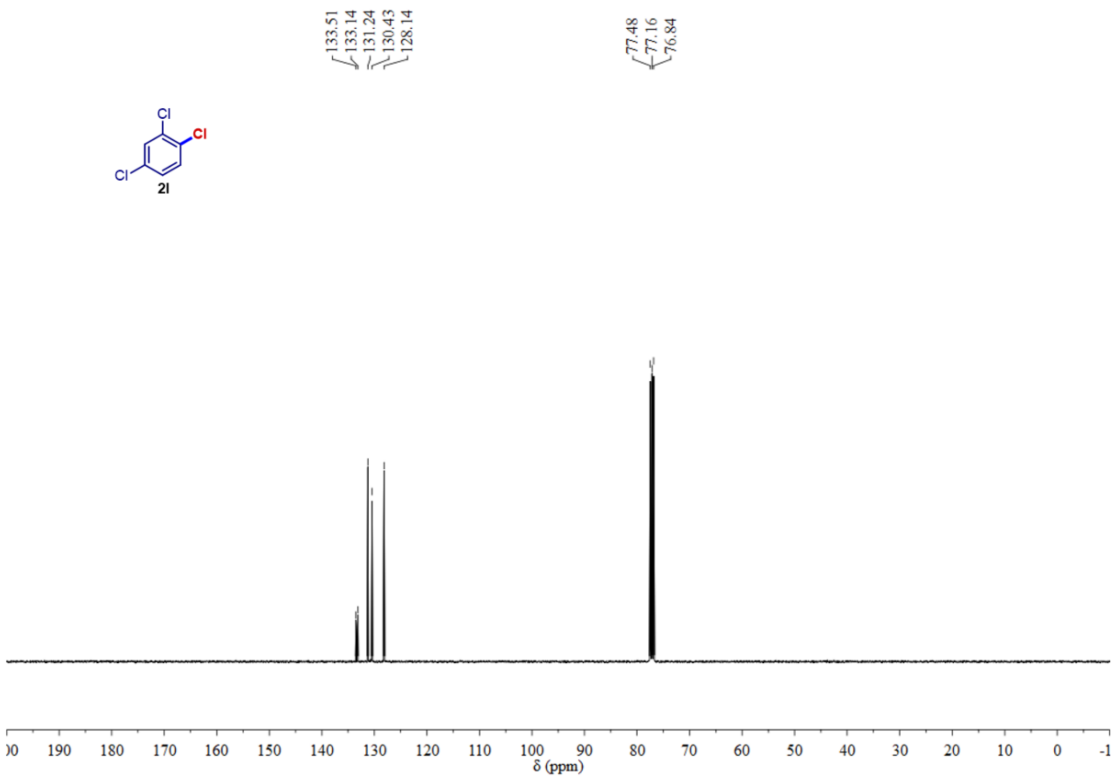

Figure S125. ^{13}C NMR spectra of **2l**

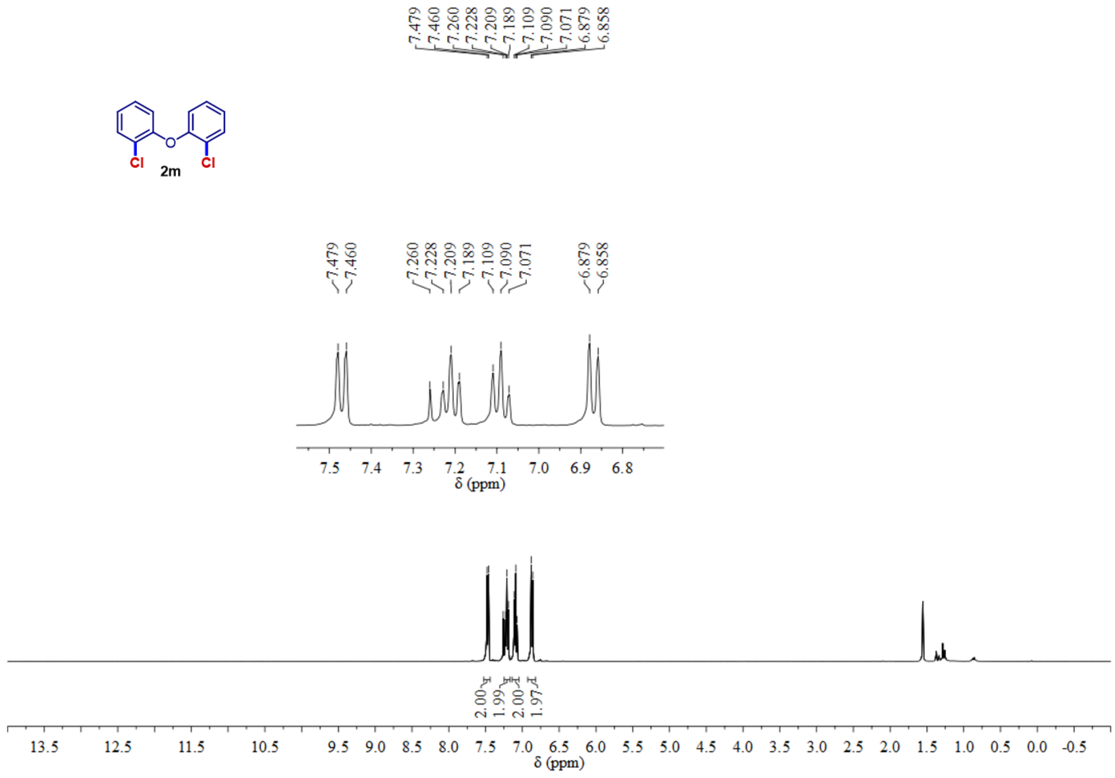

Figure S126. ^{1}H NMR spectra of **2m**

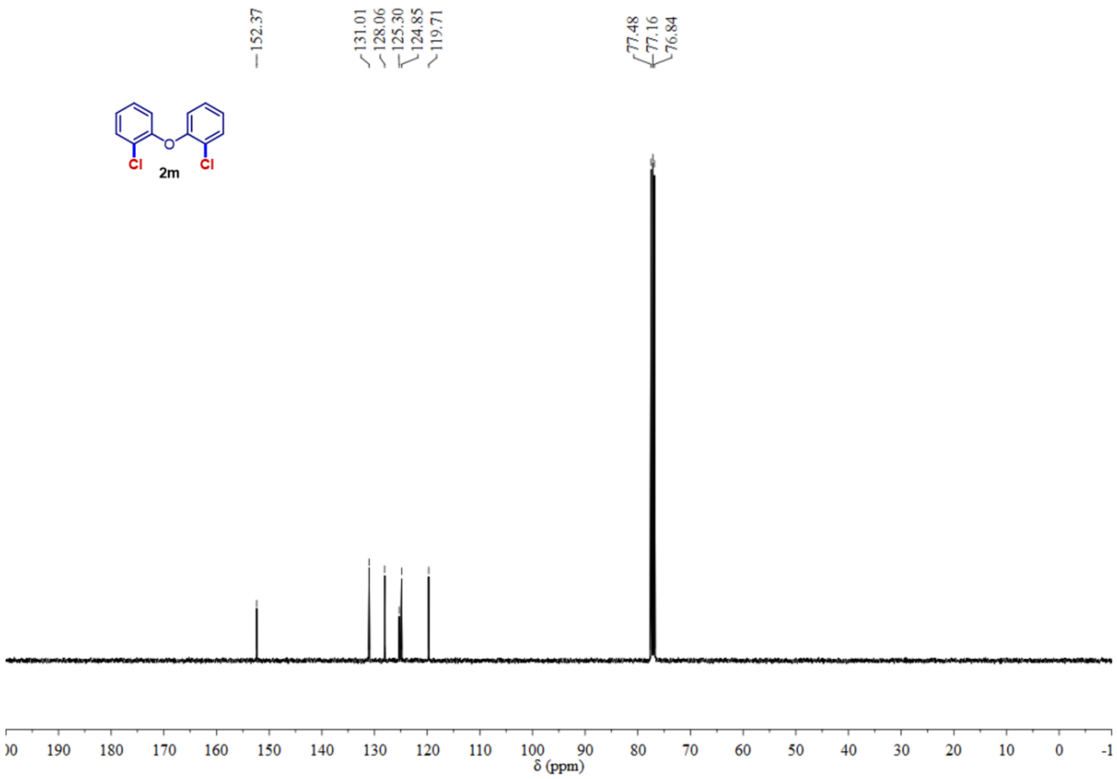

Figure S127. ^1H NMR spectra of **2m**

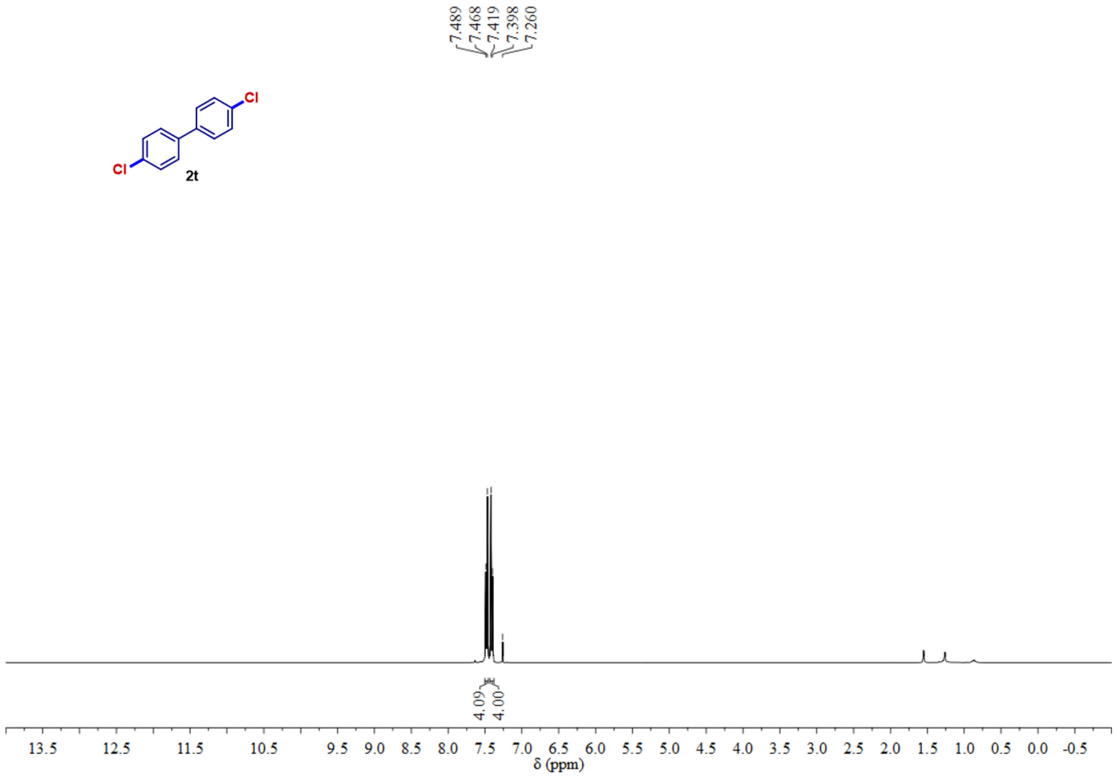

Figure S128. ^1H NMR spectra of **2t**

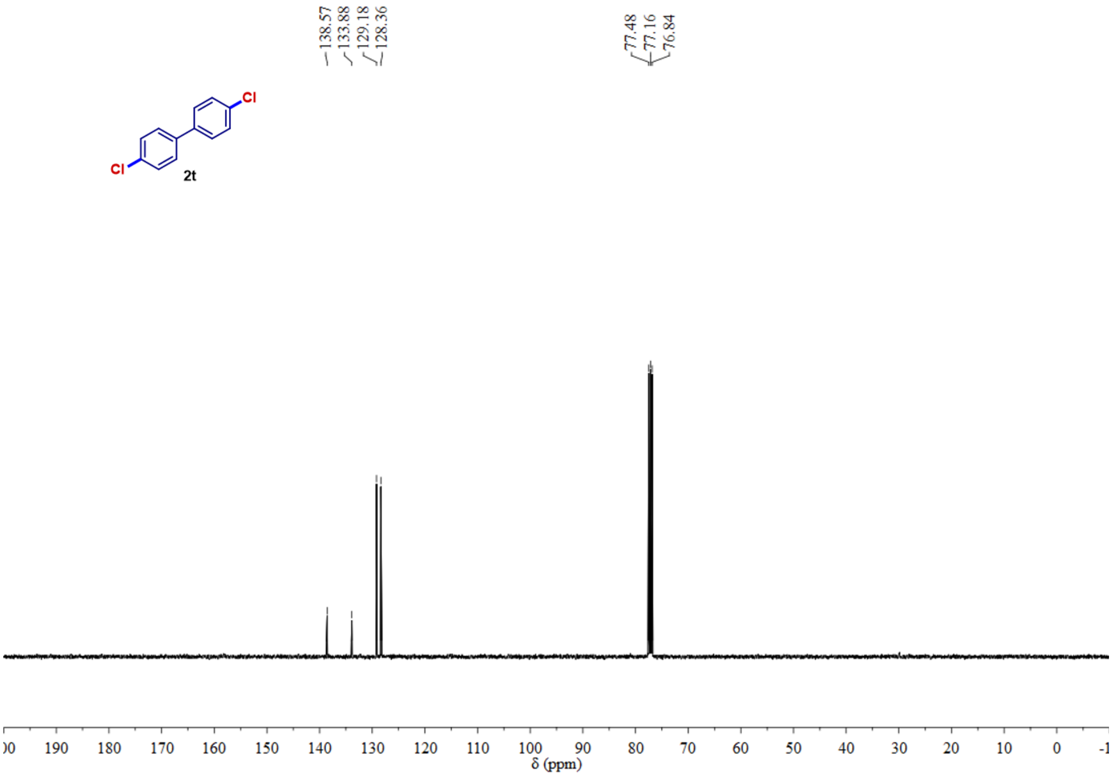

Figure S129. ^{13}C NMR spectra of **2t**

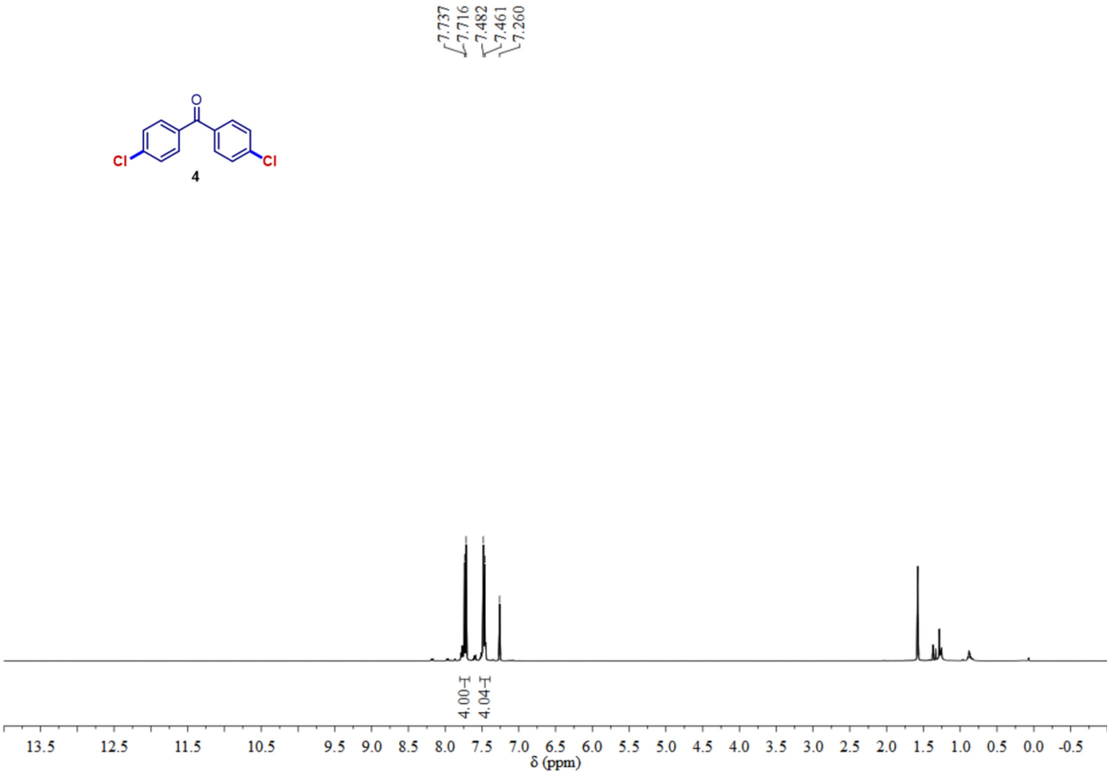

Figure S130. ^{1}H NMR spectra of **4**

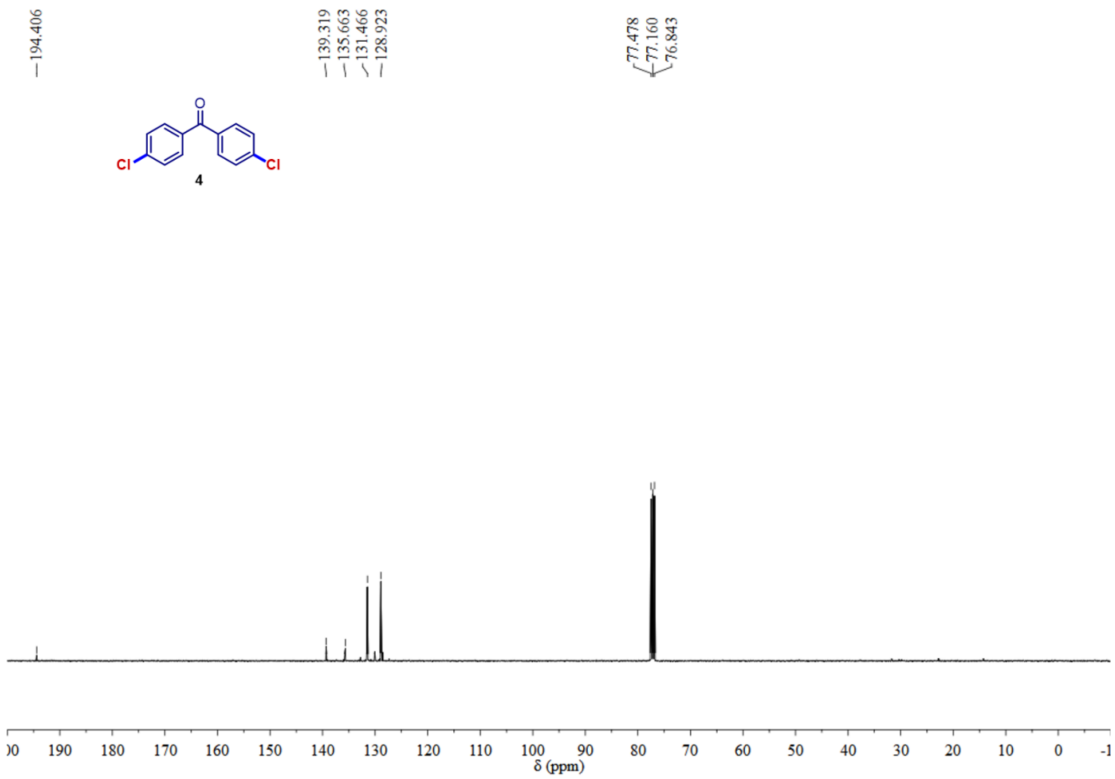

Figure S131. ^{1}H NMR spectra of **4**

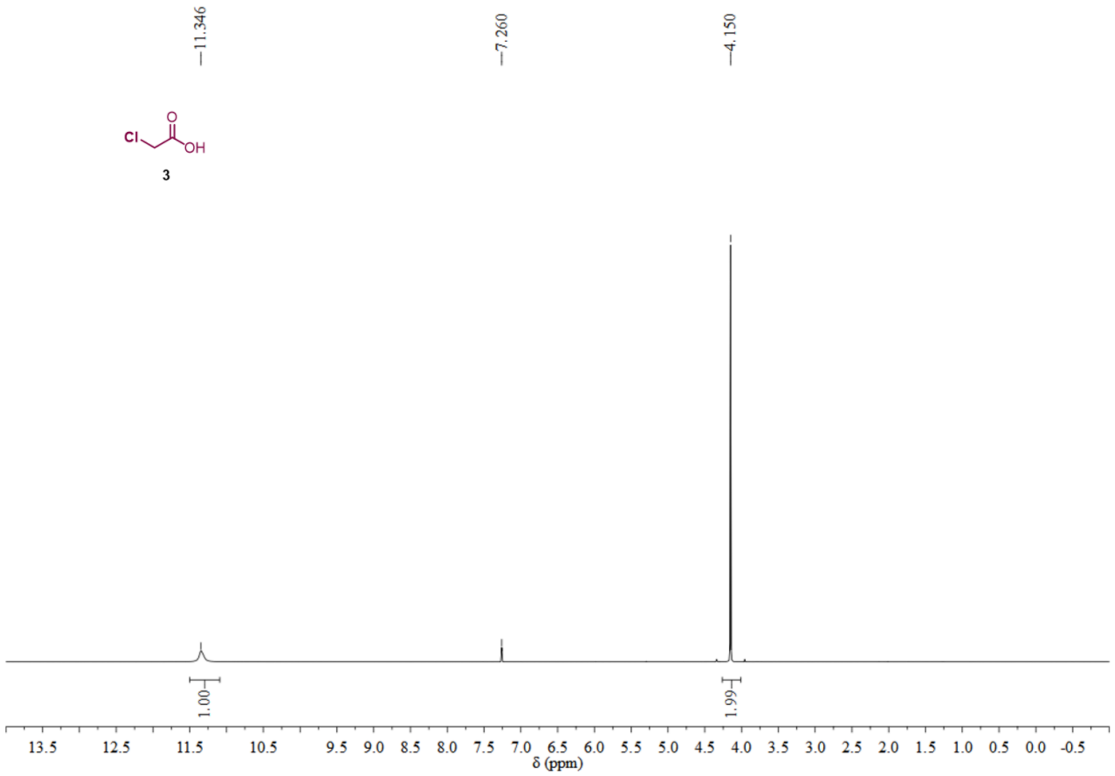

Figure S132. ^{1}H NMR spectra of **3**

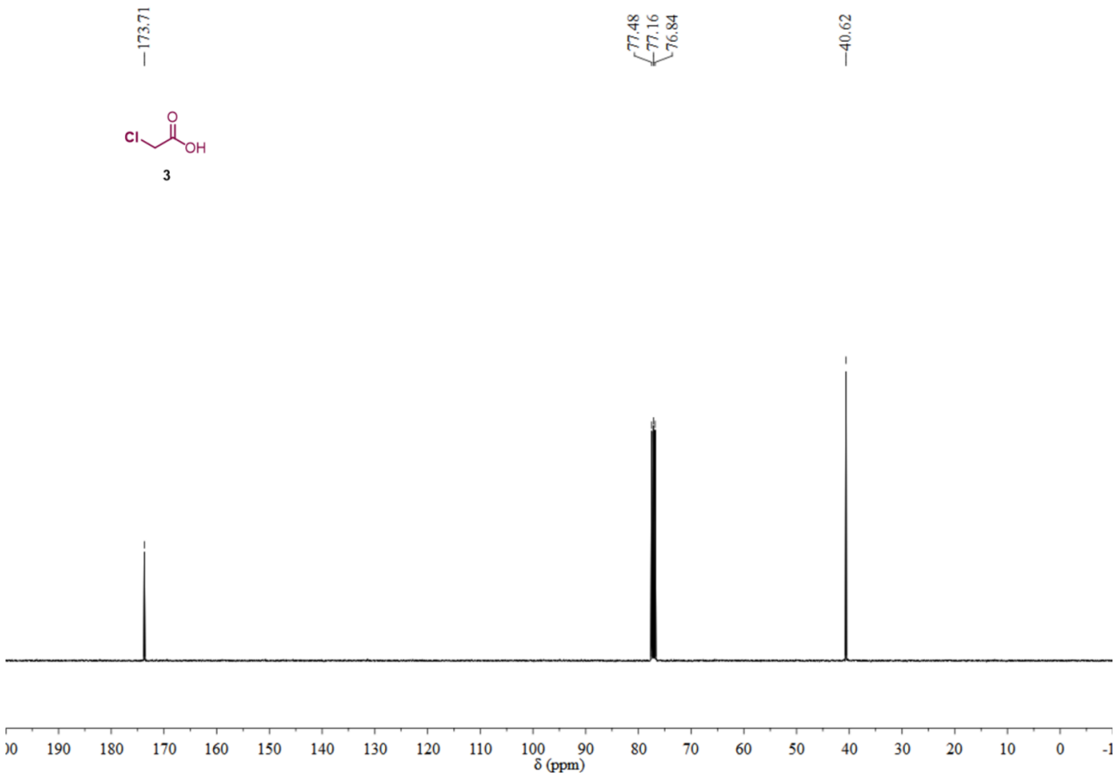

Figure S133. ^{1}H NMR spectra of **3**
